# Analogue-Sensitive Inhibition of Histone Demethylases Uncovers Member-Specific Function in Ribosomal Protein Synthesis

**DOI:** 10.1021/jacs.4c13870

**Published:** 2025-01-14

**Authors:** Jordan Kuwik, Valerie Scott, Sara Chedid, Stephanie Stransky, Kathryn Hinkelman, Sam Kavoosi, Michael Calderon, Simon Watkins, Simone Sidoli, Kabirul Islam

**Affiliations:** † Department of Chemistry, University of Pittsburgh, Pittsburgh, Pennsylvania 15260, United States; ‡ Department of Biochemistry, Albert Einstein College of Medicine, Bronx, New York 10461, United States; § Department of Cell Biology, University of Pittsburgh, Pittsburgh, Pennsylvania 15260, United States

## Abstract

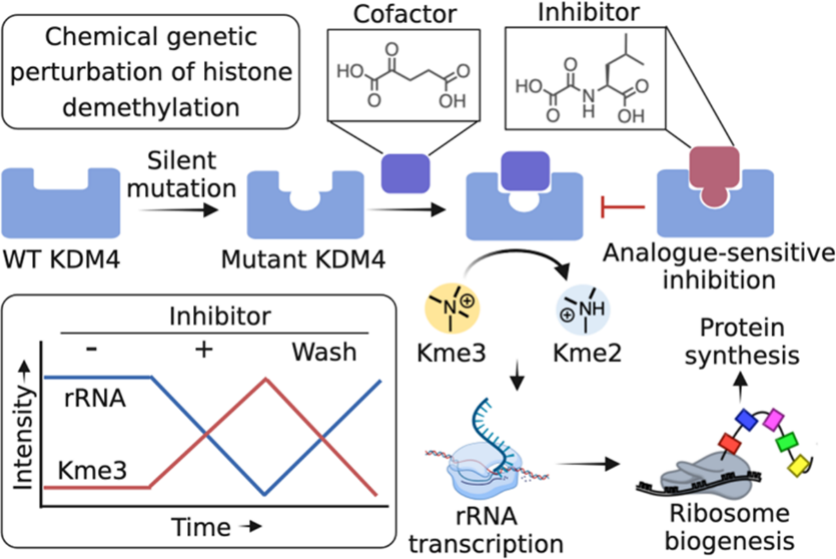

Lysine demethylases (KDMs) catalyze the oxidative removal of the methyl group from histones using earth-abundant iron and the metabolite 2-oxoglutarate (2OG). KDMs have emerged as master regulators of eukaryotic gene expression and are novel drug targets; small-molecule inhibitors of KDMs are in the clinical pipeline for the treatment of human cancer. Yet, mechanistic insights into the functional heterogeneity of human KDMs are limited, necessitating the development of chemical probes for precision targeting. Herein, we identify analogue-sensitive (*as*) mutants of the KDM4 subfamily to elucidate member-specific biological functions in a temporally defined manner. By replacing the highly conserved phenylalanine residue in the active site of KDM4 members with alanine, we develop mutants with intact catalytic activity and substrate specificity indistinguishable from those of the wild type congener. Unlike the wild type demethylases, mutants were sensitized toward cofactor-competitive N-oxalyl glycine (NOG) analogues carrying complementary steric appendage. Particularly notable is N-oxalyl leucine (NOL) which inhibited the KDM4 mutants reversibly with submicromolar efficacy. Cell-permeable NOL prodrugs inhibited *as* enzymes in cultured human cells to modulate lysine methylation on nucleosomal histones. Through conditional perturbation of the orthogonal enzymes, we uncover a KDM4A-specific role in ribosomal protein synthesis and map a remarkably dynamic signaling cascade involving locus-specific histone demethylation leading to fast rRNA expression, enhanced ribosome assembly, and protein synthesis. The results provide a mechanistic clue into KDM4A’s role in cancers that rely on heightened ribosomal activity to support uncontrolled cellular proliferation.

## Introduction

**Figure 1:**
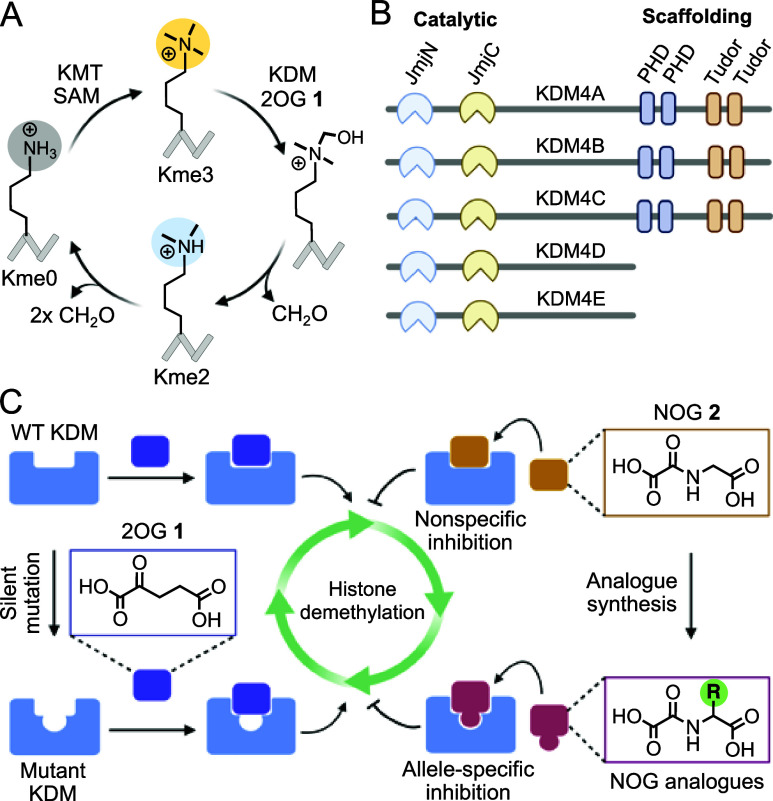
Analogue-sensitive (*as*) chemical genetic approach to probe lysine demethylases (KDMs). (A) Reversible lysine methylation on histone is maintained by lysine methyltransferases (KMTs) and KDMs. Complete demethylation is achieved by KDM-mediated sequential oxidative formation of hemiaminal, followed by release of formaldehydes. (B) KDM4 domain arrangements. JmjN and JmjC together form the catalytic domain. Tudor domains recognize methyllysine residues in histones. (C) Schematic of the *as* inhibition strategy. Both wild type and an engineered KDM carrying silent mutation perform histone demethylation using cofactor 2OG (**1**). While the wild type 2OG enzymes are inhibited nonspecifically by cofactor-competitive NOG (**2**), engineered protein is specifically sensitized to shape-complemented NOG analogues resulting in allele-specific perturbation. SAM: S-adenosyl methionine, 2OG: 2-oxoglutarate, PHD: plant homeodomain, NOG: N-oxalyl glycine.

Reversible histone methylation has emerged as a key mechanism of eukaryotic gene expression.^1,2^ The forward step is catalyzed by lysine and arginine methyltransferases (KMTs and RMTs, respectively) using S-adenosyl methionine (SAM) as the electrophilic methyl donor (Figure 1A).^3^ The demethylation step involves oxidative C–H hydroxylation orchestrated by flavin-dependent lysine-specific demethylase 1 (LSD1) or Fe^2+^ and 2-oxoglutarate (2OG, **1**)-dependent JmjC-domain-containing lysine demethylases (KDMs);^4,5^ a subset of such demethylases are known to catalyze arginine demethylation as well.^6^ The turnover of lysine methylation within histones is much slower, with *t*_1/2_ for trimethylation on histone H3 at lysine 9 (H3K9me3) being about 1.3 days, comparable to that of histone itself.^7,8^ In contrast, KDM-mediated histone demethylation is highly dynamic at individual promoters, leading to rapid transcriptional activity within minutes of receiving an external stimulus.^9,10^ Such data argue for a key role of demethylases in the spatiotemporal regulation of lysine methylation.

Members of the KDM family, >30 in humans, harbor multiple domains for member-specific genome localization and histone demethylation, which contribute to their nonoverlapping activities for regulating chromatin structure, DNA repair, cell cycle progression, germ cell development, and tumorigenesis, all occurring over a wide range of time scales (Figure 1B).^11−15^ These enzymes have been in the focus for developing pharmacological and genetic probes, with several small-molecule inhibitors of KDMs already in clinical trial for human cancer.^16−22^ Yet, mechanistic insights into the functional heterogeneity of KDMs in cellular processes are limited.

Pharmacological and genetic approaches pose certain limitations despite their respective merits in elucidating the functions of biological macromolecules. Conserved active site fold and shared cosubstrates (2OG, Fe^2+^, O_2_) across the KDM family have precluded the development of subtype-selective small-molecule modulators, which frequently exhibit off-target activity toward the broader 2OG-dependent enzymes. The specificity and robustness of genetic manipulations are suitable for addressing selectivity issues, but such techniques suffer from the lack of temporal control and are potentially lethal for developmentally important genes such as the KDMs with compensatory functions; also, with the physical loss of an entire KDM protein, all of the encoded functional information is lost, making it challenging to decipher whether an observed phenotypic change results from a missing enzymatic or scaffolding function of the protein, further underscoring the need for the development of new perturbational approaches to study this class of enzymes in the spatiotemporally variable cellular environment.

Analogue-sensitive (*as*) chemical genetics has emerged as an effective alternate tactic for elucidating the member-specific function of a protein superfamily.^23−25^ In this approach, a highly specific *neo* enzyme–inhibitor pair is developed by complementary engineering of the wild type protein–ligand interface (Figure 1C). Recently, we have identified a silent mutation of KDM4A, F185A, with intact demethylase activity and reasoned that such a variant with expanded catalytic pocket could be selectively perturbed by sterically complemented molecules to afford analogue-sensitive KDM4A (*as*KDM4A).^26,27^ The ability of the engineered KDM to accept the natural cofactor 2OG eliminates issues related to the dominant negative effect observed for catalytically dead variants. We report here the discovery of N-oxalyl leucine (NOL) as an allele-specific inhibitor of the KDM4 subfamily members KDM4A-E engineered to carry an expanded catalytic pocket. The compound reversibly inhibited *as*KDM4A-mediated histone demethylation in cultured human cells, while remaining inert toward the endogenous demethylase machinery. Using the enzyme–inhibitor pairs coupled with bioorthogonal noncanonical amino acid tagging (BONCAT),^28^ we identify a KDM4A-specific role in ribosomal protein synthesis. Further mechanistic studies reveal that *as*KDM4A-mediated H3K9me3 demethylation at the promoter of rDNA (rDNA) enhances the expression of rRNA. Using temporally defined perturbation of *as*KDM4A by its cognate inhibitor, we uncover a remarkably dynamic and coordinated pathway linking locus-specific histone demethylation, rRNA expression, and ribosomal protein synthesis.

## Results and Discussion

### Identification of N-Oxalyl Leucine (NOL) as Allele-Specific Inhibitor of KDM4A

**Figure 2:**
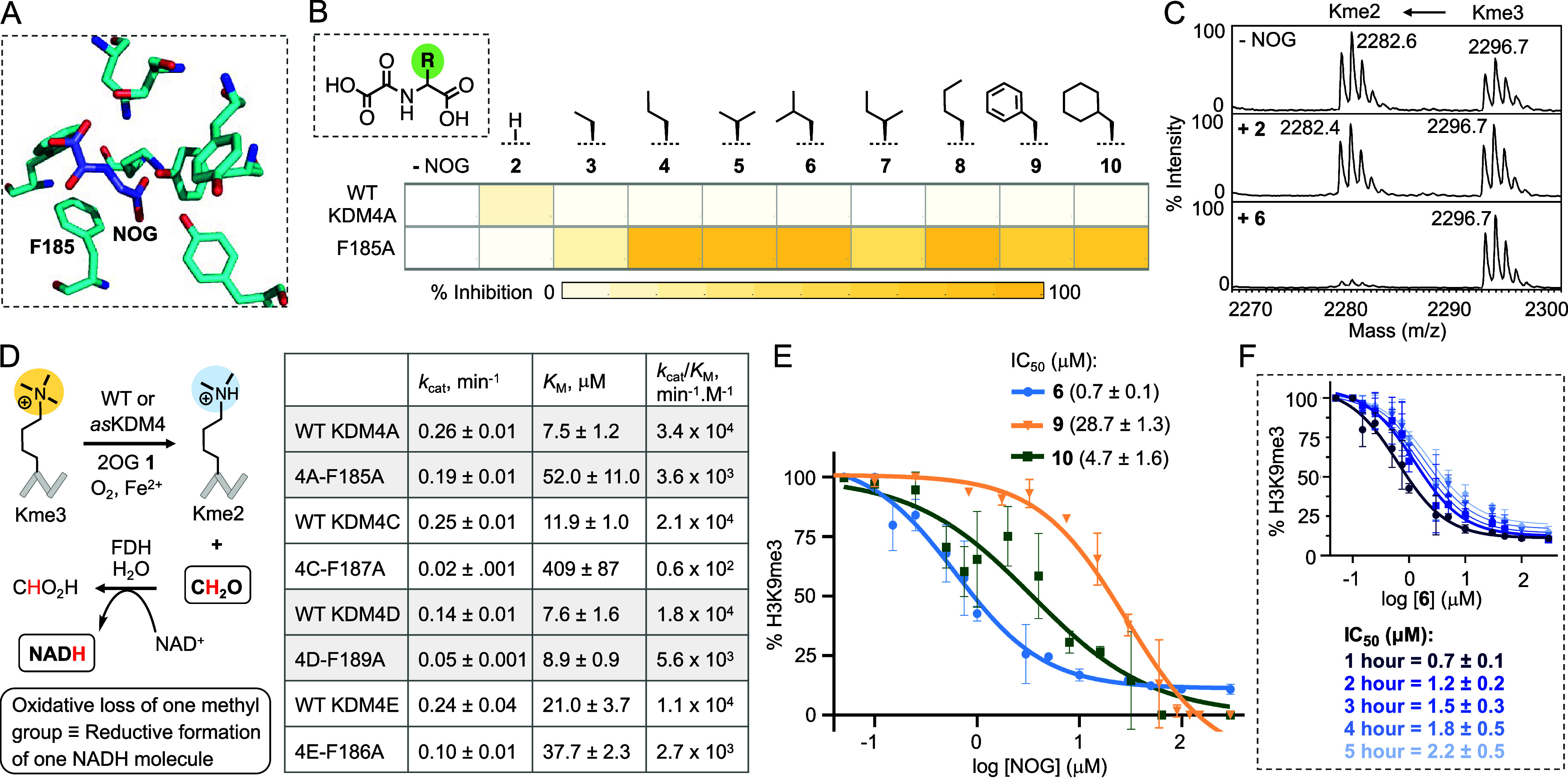
Development of analogue-sensitive (*as*) KDM4A and inhibitor pairs. (A) Crystal structure of NOG-bound KDM4A (PDB entry 6HGT) showing proximity of F185 to NOG. (B) Heat map representation of % inhibition of wild type KDM4A and its F185A mutant by a set of NOG analogues as judged by MALDI-MS. (C) Representative MALDI-MS spectra showing the activity of F185A with and without NOG analogues **2** and **6**. (D) Schematic of coupled fluorescence assay: Formaldehyde generated during demethylation of H3K9me3 peptide is oxidized to formic acid by FDH which, in turn, reduces NAD^+^ to NADH. Table of kinetic parameters for wild type and the *as* variants of KDM4 subfamily. (E) Dose-dependent inhibition of the F185A complex by **6**, **9**, and **10**. (F) Time-dependent inhibition of F185A by **6**.

To develop an appropriate inhibitor, we selected N-oxalyl glycine (NOG, **2**) scaffold because of its structural similarity with 2OG and the ability to inhibit 2OG-utilizing enzymes including KDMs (Figure 1C).^29−31^ Substituting −CH_2_– at C3 of 2OG with −NH– in NOG prevents its oxidative decarboxylation by KDMs, making the inhibitor cofactor-competitive. The crystal structure of NOG-bound KDM4A (PDB entry 6HGT) reveals the F185 residue to be positioned in close proximity to C4 of NOG (Figure 2A). We synthesized a panel of eight NOG analogues (**3**–**10**), derived from L-amino acids, carrying varied steric substituents at C4, with the reasoning that a bulky substituent at C4 would selectively fit into the expanded pocket of F185A over wild type KDM4A (Figures 2B and S1–S6, Schemes S1 and S2). The inhibitory effect of each compound was determined based on the extent of enzyme-catalyzed demethylation of a canonical peptide substrate corresponding to the N-terminus of histone 3 carrying trimethylated lysine at position 9 (H3K9me3). Both proteins demonstrated demethylase activity, as evident from the loss of methyl groups in MALDI-MS, and were weakly inhibited by the parent NOG **2** (Figures 2B, S7, and S8). The bulky analogues (**4**–**10**), although refractory to wild type KDM4A, strongly inhibited the mutant protein, illustrating how steric complementarity can be employed to achieve allele-specific perturbation (Figures 2B,C, S7, and S8). Of particular interest was NOL **6** which showed complete inhibition of the mutant without affecting wild type KDM4A. Such orthogonal inhibition of the engineered demethylase constitutes a powerful chemical probe to study the functions of a specific member of an enzyme family without issues of off-target activity.

### Quantitative Characterization of *as*KDM4A-NOL Pair

We sought to quantify the catalytic and inhibitory parameters of the enzyme–inhibitor pair since the MALDI-TOF-based end-point measurements were performed at fixed concentrations of the cofactor and inhibitors. To measure the kinetic constants for F185A and 2OG, we employed a coupled fluorescence assay involving formaldehyde dehydrogenase (FDH) to oxidize formaldehyde, the enzymatic byproduct of KDM-mediated demethylation of methyllysine, while reducing NAD+ to NADH, whose intrinsic fluorescence enables the quantification of H3K9me3 demethylation (Figure 2D).^32^ Under saturating concentration of the substrate peptide, time-dependent increase of NADH fluorescence at 340 nm as a function of 2OG concentration generated the kinetic constants (*k*_cat_, *K*_M_) and catalytic efficiency (*k*_cat_/*K*_M_) of *as*KDM4A (Figures 2D and S9). The rate constants (*k*_cat_) at which the wild type and the *as* variant catalyze H3K9me3 demethylation are comparable (0.26 ± 0.01 vs 0.19 ± 0.01 min^–1^); the affinity for 2OG, as measured by Michaelis constant (*K*_M_) is, however, about 7-fold weaker for F185A (52 ± 11 μM) compared to that for KDM4A (7.52 ± 1.2 μM), presumably due to compromised interactions between the mutant with enlarged pocket and 2OG (Figures 2D and S9), making the mutant 10-fold less catalytically efficient (*k*_cat_/*K*_M_) than wild type KDM4A; the activity is, however, comparable to several other analogue-sensitive enzymes including kinases, motor proteins, and methyltransferases.^24^ Furthermore, given that the concentration of 2OG in mammalian tissues is in the range of hundreds of micromolar,^33^ the increase in *K*_M_ is expected to have a marginal effect on the cellular activity of full-length *as*KDM4A, as observed in cell-based studies (*vide infra*).

To probe the efficacy of bulky NOGs against the mutant, the inhibitory constant at half-maximal (IC_50_) was determined by the coupled enzymatic assay using increasing amounts of the NOG analogue at fixed concentrations of cofactor and substrate. NOL **6**, phenylalanine NOG **9**, and cyclohexyl NOG 1**0** inhibited F185A with IC_50_ of 0.7 ± 0.1, 28.7 ± 1.3, and 4.7 ± 1.7 μM, respectively (Figures 2E, S10, and S11). In contrast, **6** had no inhibitory effect on wild type KDM4A up to 500 μM (Figure S12). In time-dependent measurements, **6** maintained robust inhibitory activity against *as*KDM4A with IC_50_ value slightly increased to 2.2 ± 0.5 μM when the assay was monitored over more than 5 h (Figures 2F and S13). We expect the mutant-inhibitor pair of F185A-**6** with significant catalytic efficiency, submicromolar potency, and prolonged activity to be suitable for context-dependent functional analysis of KDM4A in a complex cellular environment.

### NOL as a General Allele-Specific Inhibitor of KDM4 Subfamily

**Figure 3:**
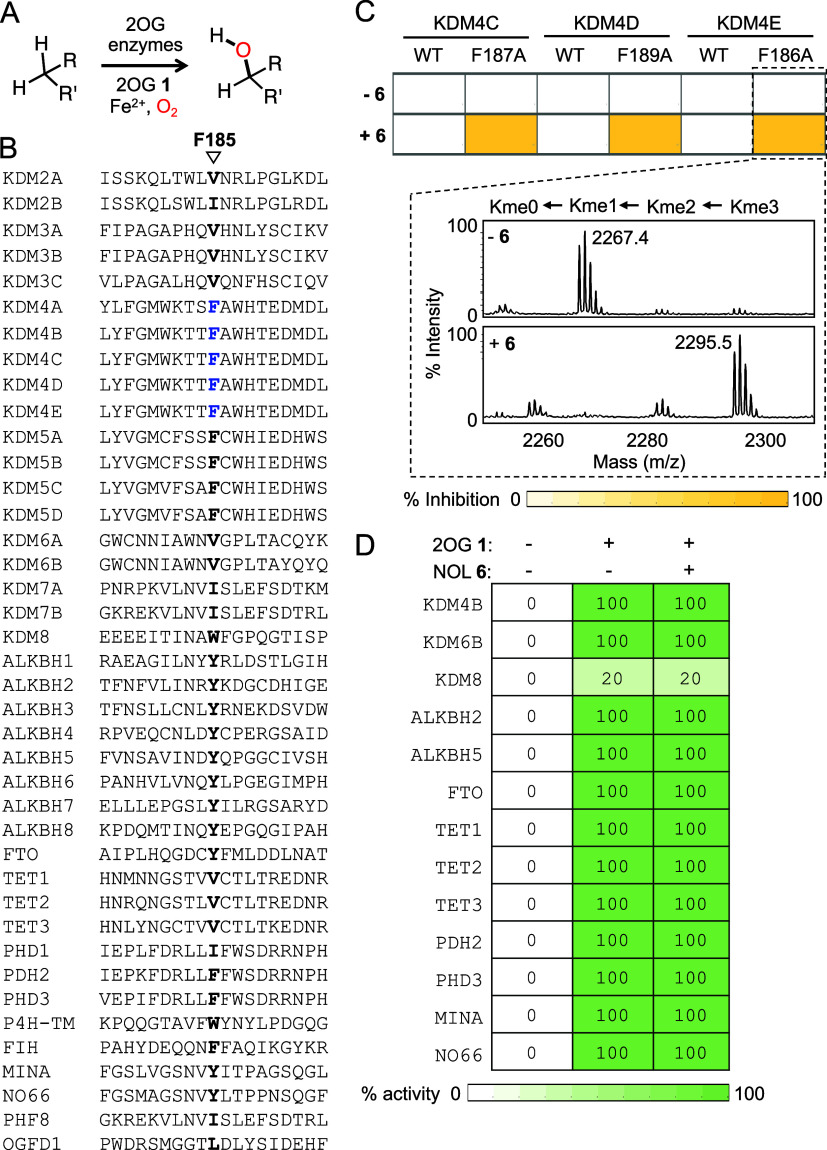
Development of *as* variants for KDM4 subfamily. (A) Scheme depicting 2OG enzyme-catalyzed C–H hydroxylation on a general substrate. (B) A bulky hydrophobic residue corresponding to KDM4A-F185 is conserved in 2OG enzyme family as revealed by structure and sequence-based analyses. (C) Heat map representation of % inhibition of wild type KDM4C, D, E and their *as* variants (F187A, F189A, and F186A, respectively) by **6**. Representative MALDI-MS spectra of the enzymatic activity of KDM4E-F186A with and without **6**. (D) Heat map representation of % activity of representative wild type 2OG enzymes with and without **6** as judged by activity assays (Figures S21–S26).

The KDM4 subfamily has 5 catalytically active members (KDM4A-E) with distinct as well as overlapping functions in transcription, DNA repair, and cancer (Figure 1B).^34−37^ More than 50% sequence similarity along with the highly conserved active site of KDM4s makes the analogue-sensitive technology particularly suitable for elucidating their member-specific activity.^38−40^ Indeed, F185 in KDM4A is highly conserved among the members and can be targeted for mutagenesis to develop homologous *as* variants (Figure 3A,B). To test the generality of our approach and versatility of NOL as an allele-specific chemical probe across the enzyme family, we recombinantly expressed F186A, F187A, F189A, and F186A mutants of KDM4B-E, respectively. Expression and purity of KDM4B–F186A were suboptimal and excluded from further biochemical analysis; we, however, demonstrated allele-specific inhibition of full-length KDM4B–F186A in mammalian cells (*vide infra*). In the *in vitro* end-point assay described above, mutant proteins, except KDM4C–F187A, showed robust H3K9me3 demethylation activity with 2OG as cofactor, as judged by MALDI-MS data (Figures 3C, S14, and S15). We also determined the catalytic parameters (*k*_cat_, *K*_M_) of wild type KDM4C-E, and their *as* variants by employing the NADH coupled assay as described above. The enzymatic proficiencies of KDM4D and E mutants are comparable to those of their wild type counterparts (Figures 2D and S16–S18). Although KDM4C–F187A showed only marginal turnover toward the peptide substrate; the corresponding full-length mutant efficiently demethylated H3 in human cells (*vide infra*), further suggesting the importance of physiological context in assessing the *as* variants.

We next tested the inhibitory potential of **6** against these proteins using H3K9me3 peptide substrate and analyzed it by MALDI-MS. The activity of each *as*KDM4, but not wild type KDM4C-E, was significantly inhibited by **6** (Figures 3C, S14, and S15). We subsequently examined the ability of *as*KDM4 variants to demethylate full-length histones extracted from mammalian cells. HEK293T cells were cultured in the presence of 10 μM n-octyl IOX1, a broad-spectrum inhibitor of demethylases,^41,42^ to generate a hypermethylated proteome (Figure S19). The extracted histone was incubated with the *as* variants individually, each with or without **6**, and the extent of demethylation was analyzed by Western blot using the H3K9me3-specific antibody. Each mutant protein indeed demethylated the full-length substrate, and this activity was noticeably inhibited by **6** (Figure S19). These results show that the mutation of a highly conserved phenylalanine residue to alanine in the active site of KDM4s does not ablate their demethylase activity with the canonical cofactor to a significant degree but sensitizes the variants to a sterically complemented inhibitor which is fully refractory to the wild type enzymes.

We sought to examine the orthogonality of compound **6** across the human 2OG enzyme family. Analyses of protein sequences and reported crystal structures revealed that the amino acids corresponding to F185 in KDM4A are highly conserved among the 2OG-dependent dioxygenases and occupy an analogous position with respect to 2OG or NOG in the active sites (Figures 3A,B and S20). These amino acids, bulky and hydrophobic in nature, likely act as the gatekeeper and preclude binding of the NOG analogues to wild type enzymes. To test the off-target activity of **6**, we recombinantly expressed the catalytic domains of 12 wild type dioxygenases, in addition to KDM4A-E, thus covering 30–40% of human 2OG enzymes known to have well-characterized substrates (Figure 3D). The set of enzymes possess diverse catalytic activity (e.g., histone, DNA and RNA demethylation, and prolyl, histidyl, and arginine hydroxylation) with distinct cellular localization and biological functions. The enzymes showed robust activity on their cognate substrates, such as peptides, short oligonucleotides, or genomic DNA isolated from human cells, as evident from MALDI-MS spectra or dot blots with appropriate antibodies (Figures 3D and S21–S26). Importantly, **6** did not inhibit any of the wild type enzymes at concentrations up to 200 μM (Figures 3D and S21–S26).^43−45^ These results convincingly demonstrate that wild type 2OG enzymes are not inhibited by **6** due to the presence of a bulky gatekeeper amino acid, and the *as* perturbation is achieved primarily through a space-creating mutation at the conserved residue.

### Assessing the Activity of Orthogonal Mutant-Inhibitor Pairs in Mammalian Cells

**Figure 4:**
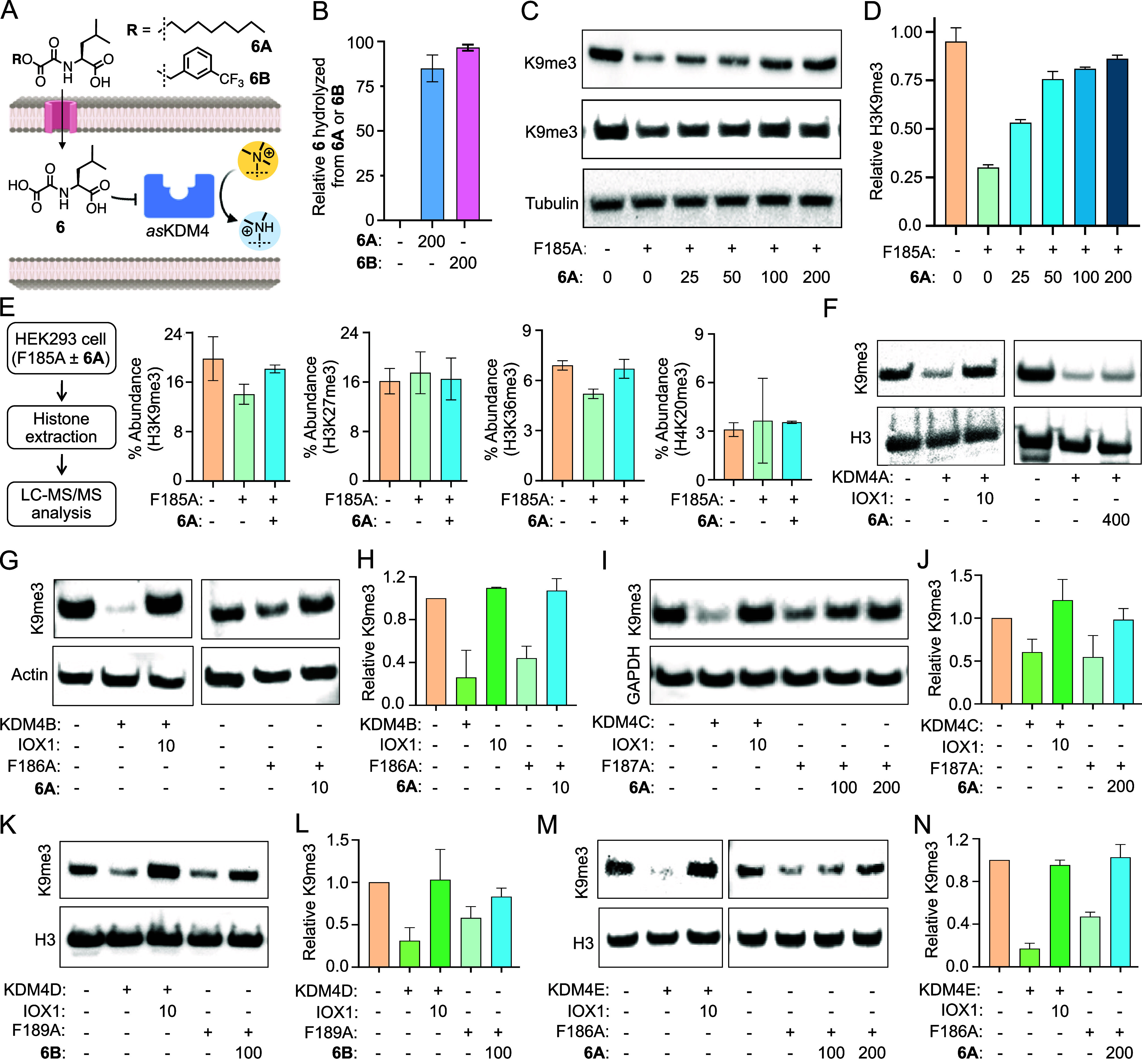
*In cellulo* activity of *as*KDM4-inhibitor pairs. (A) Chemical structures of cell-permeable octyl (**6A**) and trifluoromethyl benzyl (**6B**) ester prodrugs of **6**. Both the compounds are hydrolyzed to active compound **6** in cells resulting in inhibition of *as*KDM4. (B) Quantitative representation of the relative amount of **6** hydrolyzed from **6A/B** in HEK293T cells as determined by LC-HRMS analysis of cell extracts. (C) Dose-dependent inhibition of F185A by 6A in HEK293T cells as judged by Western blot analysis. (D) Bar diagram representation of F185A inhibition by 6A based on the Western blot data in Figures 4C and S28 (*n* = 4). (E) Flowchart showing steps involved in histone extraction from HEK293T cells expressing the F185A ± 6A pair followed by tandem mass spectrometry. Bar diagram showing quantitative changes in H3K9me3 and H3K36me3 based on the LC-MS/MS results provided in Table S1 (*n* = 2). (F) Inhibition of wild type KDM4A by 10 μM n-octyl IOX1, but not by 400 μM 6A, in HEK293T cells. (G) Inhibition of wild type KDM4B and its F186A mutant by n-octyl IOX1 and 6A, respectively, each at 10 μM concentration, in HEK293T cells. (H) Bar diagram representing changes in H3K9me3 based on the Western blot data provided in (G) and Figure S32 (*n* = 4). (I) Inhibition of wild type KDM4C and its F187A mutant in HEK293T cells by n-octyl IOX1 and 6A, respectively. (J) Bar diagram showing changes in H3K9me3 based on the Western blot data provided in (I) and Figure S33 (*n* = 3). (K) Inhibition of wild type KDM4D and its F189A mutant by n-octyl IOX1 and 6B, respectively, in HEK293T cells. (L) Bar diagram showing changes in H3K9me3 based on the Western blot data provided in (K) and S34 (*n* = 4). (M) Inhibition of wild type KDM4E and its F186A mutant by n-octyl IOX1 and 6A, respectively, in HEK293T cells. (N) Bar diagram indicates changes in H3K9me3 based on the Western blot data provided in (M) and Figure S35 (*n* = 4). *n* indicates independent replicates. Inhibitor concentrations are in μM.

We next examined the orthogonal pairs for their ability to modulate lysine demethylation on the chromosomal histone in cultured human cells (Figure 4A). To this end, we generated the F185A mutation in full-length KDM4A carrying all of the functional domains. Furthermore, to enhance cell permeability, we synthesized two chemoselective ester prodrugs **6A** and **6B** carrying either an n-octyl or a trifluoromethyl benzyl ester as the lipophilic moiety, respectively (Figures 4A, S5, and S6, Scheme S3).^46,47^ We confirmed the cell permeability of **6A** and **6B** and their subsequent hydrolysis to **6** by analyzing extracts of compound-treated HEK293T cells using LC-HRMS. Both compounds displayed comparable membrane permeability based on total ion counts (Figures 4B and S27). We also performed MTT assay and observed that the compounds have marginal cytotoxicity in HEK293T cells at near millimolar concentrations (Figure S27).

HEK293T cells were transiently transfected with *as*KDM4A and treated with varying concentrations of **6A** or **6B**. Western blot analysis of nuclear extracts showed the robust demethylation of H3K9me3 in *as*KDM4A transfected cells, confirming that the mutant is enzymatically active in cells with endogenous 2OG (Figure 4C). Importantly, *as*KDM4A was inhibited by both prodrugs in a dose-dependent manner, leading to H3K9me3 levels comparable to that of cells not expressing the mutant demethylase (Figures 4C,D, S28, and S29). To further confirm the activity of the enzyme–inhibitor pair, we performed tandem mass spectrometry of tryptic histones isolated from HEK293T cells expressing *as*KDM4A in the presence or absence of 100 μM **6A**. Analysis of the LC-MS/MS data of two independent biological replicates indeed showed a moderate decrease in the relative abundance of H3K9me3 and H3K36me3 marks compared to nontransfecting cells (Figure 4E, Table S1). Both modifications, however, recovered to the level of the control vector upon treatment with **6A**, confirming that the *as*KDM4A-**6** pair is functional in cells. It is important to note that the relative abundance of other key histone methylation and acetylation sites remained unaffected (Figures 4E and S30, Table S1), confirming the *in cellulo* substrate specificity of the enzyme–inhibitor pair.

To test the orthogonality of NOL toward wild type KDM4A, HEK293T cells, either nontransfected or transfected with KDM4A, were individually treated with DMSO, 400 μM **6A** or 10 μM n-octyl IOX1, a nonspecific inhibitor of 2OG enzymes. The wild type enzyme efficiently demethylated cellular histone H3 and was strongly inhibited by IOX1, but not **6A**, as seen in Western blot using H3K9me3 antibody (Figure 4F). To further probe for off-target effects of **6** on endogenous 2OG enzymes, HEK293T cells were cultured with DMSO or 400 μM **6A** and examined for several postsynthetic modifications catalyzed by these dioxygenases. Key trimethylated lysine marks on extracted histone were analyzed by Western blot using corresponding antibodies (Figure S31). No change was observed in trimethylated K4, K9, K27, and K36 levels in histone H3, consistent with our tandem mass spectrometric results described above. Similarly, no change in prolyl hydroxylation was detected when cellular extracts were subjected to the *pan* hydroxyproline antibody. The bulky inhibitor also had no effect on 5mC, 5hmC, and m6A modifications on DNA and RNA, as revealed by dot blot assays (Figure S31). These results confirm that NOL is specific for the *as* variant and does not interfere with the activity of endogenous machinery regulating histone, RNA and DNA methylation/demethylation, and prolyl hydroxylation, further corroborating the *in vitro* selectivity profile of **6**.

To demonstrate that NOL can inhibit the other *as* variants of KDM4 subfamily in human cells, we generated the full-length *as* mutants of the remaining KDM4s (KDM4B-F186A, KDM4C-F187A, KDM4D-F189A, and KDM4E-F186A). KDM4B and C are structurally homologous to KDM4A, and KDM4D and E have a catalytic pocket near-identical to that of KDM4A but lack the Tudor and PHD domains known to bind trimethylated lysine in histones (Figure 1B).^11^ HEK293T cells were transfected with full-length wild type KDM4B-E or their respective mutants. Western blot analysis of nuclear preparations using the H3K9me3 antibody confirmed all of the proteins to be catalytically active (Figures 4G–N and S32–S35). We noted that the activity of wild type proteins and that of the mutants were significantly reduced when cells were treated with n-octyl IOX1 or **6A**/**B**, respectively, in a dose-dependent manner (Figures 4G–N and S32–S35); particularly notable is the robust inhibition of *as*KDM4B by **6A**/**B** at 10 μM concentration (Figures 4G,H and S32). Such results suggest that NOL will be effective as a general inhibitor in perturbing *as* variants of the KDM4 subfamily in mammalian cells.

### Probing Member-Specific Role of KDM4 in Ribosomal Protein Synthesis

**Figure 5:**
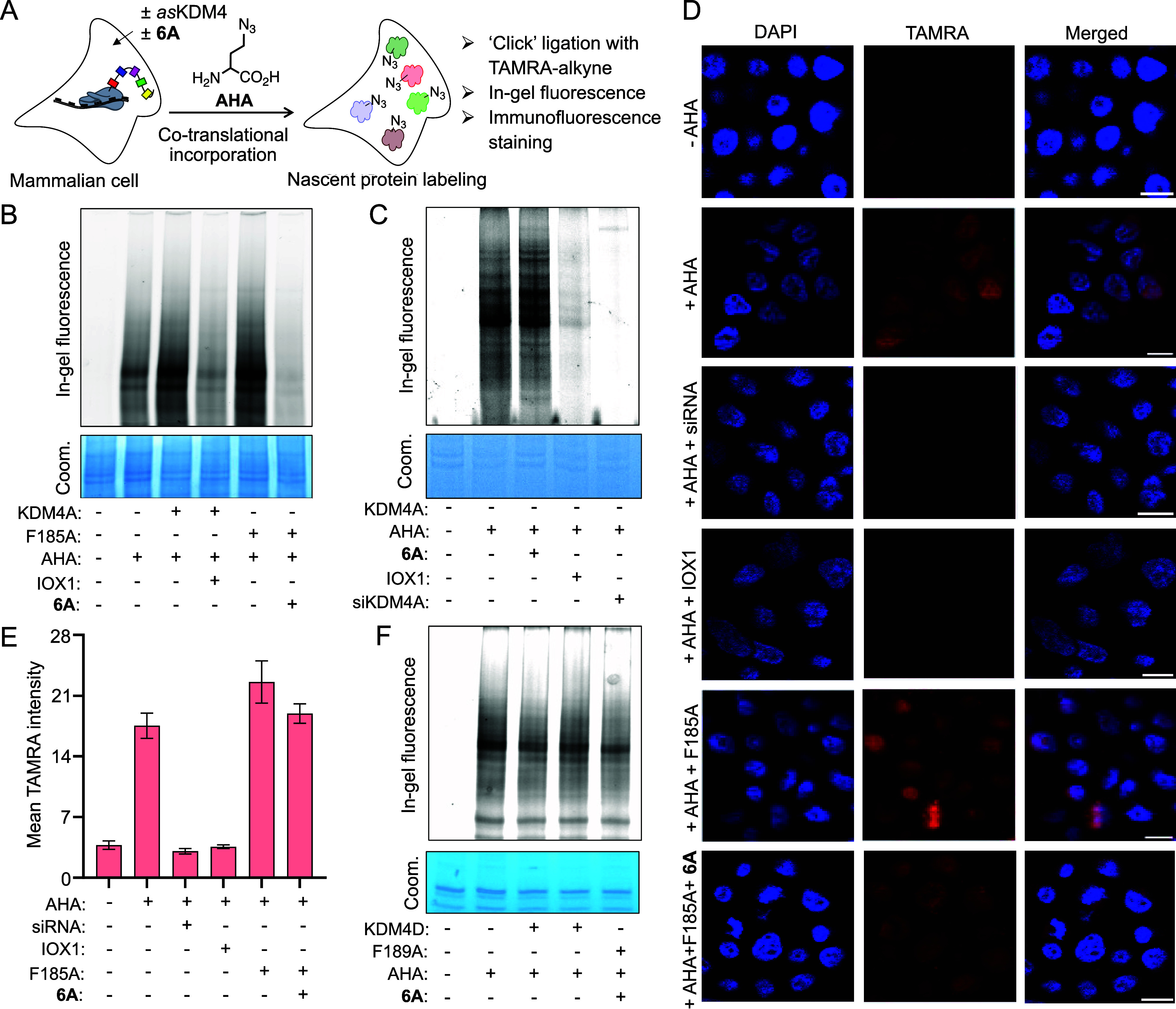
KDM4 member-specific role in ribosomal protein synthesis. (A) Schematic of BONCAT approach to label nascent proteins by pulsing cells with azidohomoalanine (AHA), followed by “clicking” cell extracts with TAMRA alkyne. (B) In-gel fluorescence data showing increase in protein synthesis by exogenously expressed wild type KDM4A and F185A, which are subsequently inhibited by n-octyl IOX1 and **6A**, respectively. Coomassie staining to ensure equal protein loading. (C) Inhibition and silencing of endogenous KDM4A in HEK293T cells by n-octyl IOX1 and siRNA, respectively, led to protein synthesis inhibition, as revealed by in-gel fluorescence. **6A** had no effect on protein synthesis. (D) Confocal imaging of TAMRA alkyne ligated nascent proteins in fixed HEK293T cells (scale = 10 μm). (E) Mean TAMRA fluorescence intensity as a measure of nascent protein synthesis based on the fixed cell imaging provided in (D) (average number of cells: 20). (F) Wild type KDM4D and F189A mutant failed to upregulate protein synthesis as judged by in-gel fluorescence.

Biochemical integrity and robust orthogonality of *as*KDM4-NOL pairs in cells strongly argue for their applicability in elucidating the regulatory functions of KDM4 members in cellular processes. For example, KDM4A has been shown to partake in global protein synthesis;^48,49^ however, mechanistic insight into how histone demethylation mediated by a specific KDM4 isoform regulates ribosomal processes is lacking. To investigate the role of KDM4A in protein synthesis, we adapted the bioorthogonal noncanonical amino acid tagging (BONCAT) tactic which relies on the translational incorporation of an unnatural amino acid probe into newly synthesized proteins in cells (Figure 5A).^28^ HEK293T cells expressing wild type KDM4A or its F185A mutant were treated with n-octyl IOX1 or **6A**, and the synthesis of nascent proteins was tracked by pulsing the cells with azidohomoalanine (AHA). Isolated cellular extracts were subjected to copper-catalyzed click ligation with TAMRA alkyne,^50^ separated on polyacrylamide gel and visualized at 532 nm light (*l*_max_ for TAMRA) to assess AHA incorporation. Overexpression of wild type KDM4A and the *as* variant (F185A) increased the amount of newly synthesized proteins compared to control vector-transfected cells (Figure 5B). Furthermore, the respective inhibitors led to a sharp decline in protein synthesis, as evident from the significant loss of the TAMRA signal (Figure 5B).

We next examined the role of endogenous KDM4A in protein synthesis. siRNA-mediated knockdown of KDM4A in HEK293T cells led to a substantial decrease in protein synthesis; consistently, treatment with 10 μM of n-octyl IOX1 had a similar effect (Figure 5C). In contrast, treatment of HEK293T cells with 200 μM of **6A** failed to inhibit protein synthesis mediated by endogenous wild type KDM4A, further confirming its specificity toward the *as* variant (Figure 5C). To visualize the nascent proteins in intact cells, we performed click ligation on fixed cells with TAMRA alkyne, followed by confocal imaging. Treatment with AHA led to a robust fluorescence signal primarily in the cytoplasm compared to untreated cells indicating cytoplasmic protein synthesis (Figure 5D,E). Silencing of endogenous KDM4A resulted in a significant reduction in TAMRA signal, which was phenocopied by n-octyl IOX1 treatment, consistent with in-gel fluorescence results. Furthermore, HEK293T cells overexpressing F185A mutant moderately increased protein synthesis which was subsequently inhibited by **6A** to the level of cells expressing control vector (Figure 5D,E). These results present strong evidence that the catalytic activity of KDM4A is important for ribosomal protein synthesis and that *as*KDM4A-NOL is suitable for interrogating KDM4 function in mammalian cells.

To examine if some of the other KDM4 members played any role in protein synthesis, we selected KDM4D because it differs from KDM4A in domain organization and substrate preference, despite sharing the highly conserved active site.^51^ HEK293T cells were transiently transduced with wild type KDM4D, or its *as* variant (F189A), and exposed to AHA followed by click ligation and in-gel fluorescence. Intriguingly, unlike KDM4A, both proteins, although catalytically active, failed to enhance ribosomal activity for protein synthesis (Figure 5F). The distinctive role of KDM4A in protein upregulation likely stems from its differential genome localization and promoter-specific activity mediated by the tandem Tudor and PHD domains, which are absent in KDM4D. Together, the results show that *as*KDM4 and the cognate inhibitor pairs are well suited to elucidate KDM4 member-specific functions in cellular processes as illustrated here for KDM4A versus KDM4D in the context of ribosomal protein synthesis.

### *as*KDM4A-NOL Regulates Promoter Demethylation for Expression of rRNA

**Figure 6:**
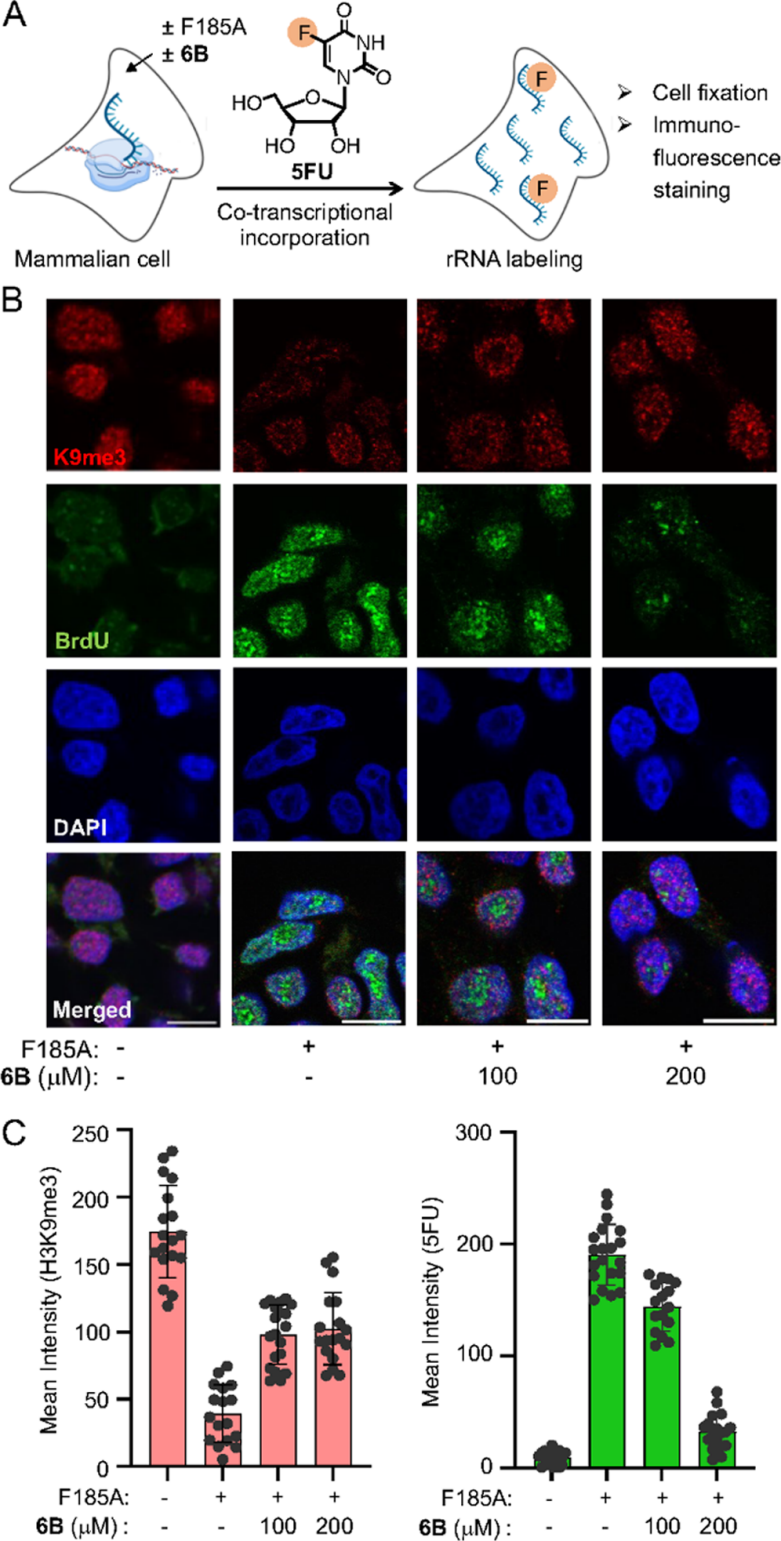
*as*KDM4A-NOL pair regulates rRNA transcription. (A) Schematic of cotranscriptional incorporation of 5-fluorouridine (5FU) into rRNA followed by cell fixation and imaging. (B) Fixed cell immunofluorescence staining of HEK293T cells expressing F185A with or without **6B** using H3K9me3 and 5-bromouridine (BrdU) antibodies (scale = 10 μm). (C) Quantitative representation of accompanying changes in H3K9me3 and 5FU levels (number of cells: 16–20).

Upregulation of protein synthesis is linked to enhanced ribosomal biogenesis driven by the expression and coordinated assembly of the ribosomal RNAs (rRNAs). To systematically investigate the role of KDM4A in rRNA activation, HEK293T cells expressing wild type KDM4A or *as*KDM4A were briefly (∼15 min) exposed to 5-fluorouridine (5FU), a uridine analogue known to rapidly incorporate into nascent RNAs, in the presence of cell-permeable n-octyl IOX1 or NOL derivative **6B**, respectively (Figure 6A). Given that there are about 350 copies of rDNA in the human genome, brief exposure to 5FU ensures the labeling of rRNAs primarily.^49^^52^ The cells were then fixed and stained with H3K9me3 antibody, along with BrdU antibody which recognizes 5FU, to examine any correlation between histone demethylation and rRNA expression using high-resolution immunofluorescence imaging. Both wild type KDM4A and F185A mutant led to robust increase in 5FU incorporated rRNA levels, while reduction in H3K9me3 signals was observed (Figures 6B,C and S36). Importantly, treatment with **6B** attenuated 5FU incorporation in a dose–response manner, with concurrent re-emergence of H3K9me3 levels as a result of mutant inhibition (Figures 6B,C and S36). Similarly, n-octyl IOX1 repressed histone demethylation and 5FU incorporation mediated by the overexpressed KDM4A (Figure S36). These results confirm that the catalytic activity of KDM4A is required to induce rRNA expression.

**Figure 7:**
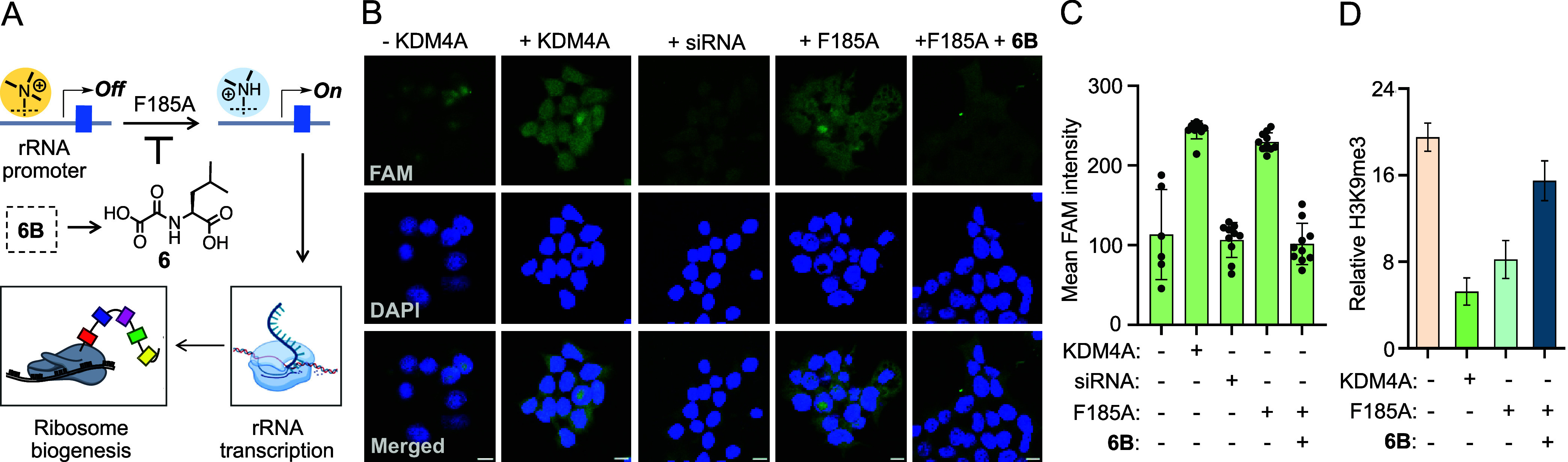
*as*KDM4A-NOL pair regulates histone demethylation at the rRNA promoter. (A) Schematic showing H3K9me3 demethylation at the rRNA promoter leading to rRNA transcription and ribosome biogenesis. **6B** is expected to hinder the process. (B) Fluorescence *in situ* hybridization (FISH) experiment with fluoresceine (FAM)-attached short DNA showing increased expression of 47S precursor rRNA by wild type KDM4A and its F185A mutant. Treatment with siRNA and **6B**, respectively, led to a decreased expression of pre-rRNA (scale = 10 μm). (C) Mean fluorescence intensity as a measure of rRNA transcription obtained from the FISH experiment (# of cells: 6–10). (D) Decrease in H3K9me3 level at the rRNA promoter by wild type KDM4A and F185A as determined by ChIP-qPCR. The activity of the mutant is robustly inhibited by **6B**.

We next examined the mechanistic link between KDM4A-mediated promoter demethylation and rRNA upregulation (Figure 7A). To confirm that KDM4A specifically stimulates rRNA synthesis, we performed fluorescence *in situ* hybridization (FISH) using a fluoresceine-attached short DNA complementary to the 47S rRNA precursor. Overexpression of wild type KDM4A and its *as* variant indeed led to increased expression of the precursor rRNA compared to that of the control vector (Figure 7B,C). More importantly, treatment with siRNA and cognate inhibitor **6B**, respectively, significantly reduced the transcription of the 47S precursor (Figure 7B,C), convincingly demonstrating the role of KDM4A in rRNA expression.

To examine histone demethylase activity at the rRNA promoter for transcriptional activation, we performed chromatin immunoprecipitation (ChIP) with a H3K9me3-specific antibody, followed by qPCR analysis using primers targeting transcriptionally competent rDNA (Figure 7A). *as*KDM4A, like wild type KDM4A, indeed led to a substantial loss of the silencing mark (H3K9me3) at the promoter as revealed by a significant decrease in rDNA enrichment, further supporting our imaging data (Figure 7D). Additionally, treatment with **6B** increased H3K9me3 at the promoter to the extent comparable to nontransfected cells (Figure 7D). Such results convincingly validate that KDM4A-mediated histone demethylation at the rDNA promoter is a crucial post-translational event for the activation of rDNA transcription, which subsequently regulates ribosomal assembly and protein synthesis.

### Probing Temporal Coordination between Histone Demethylation and rRNA Expression with *as*KDM4A-NOL

**Figure 8:**
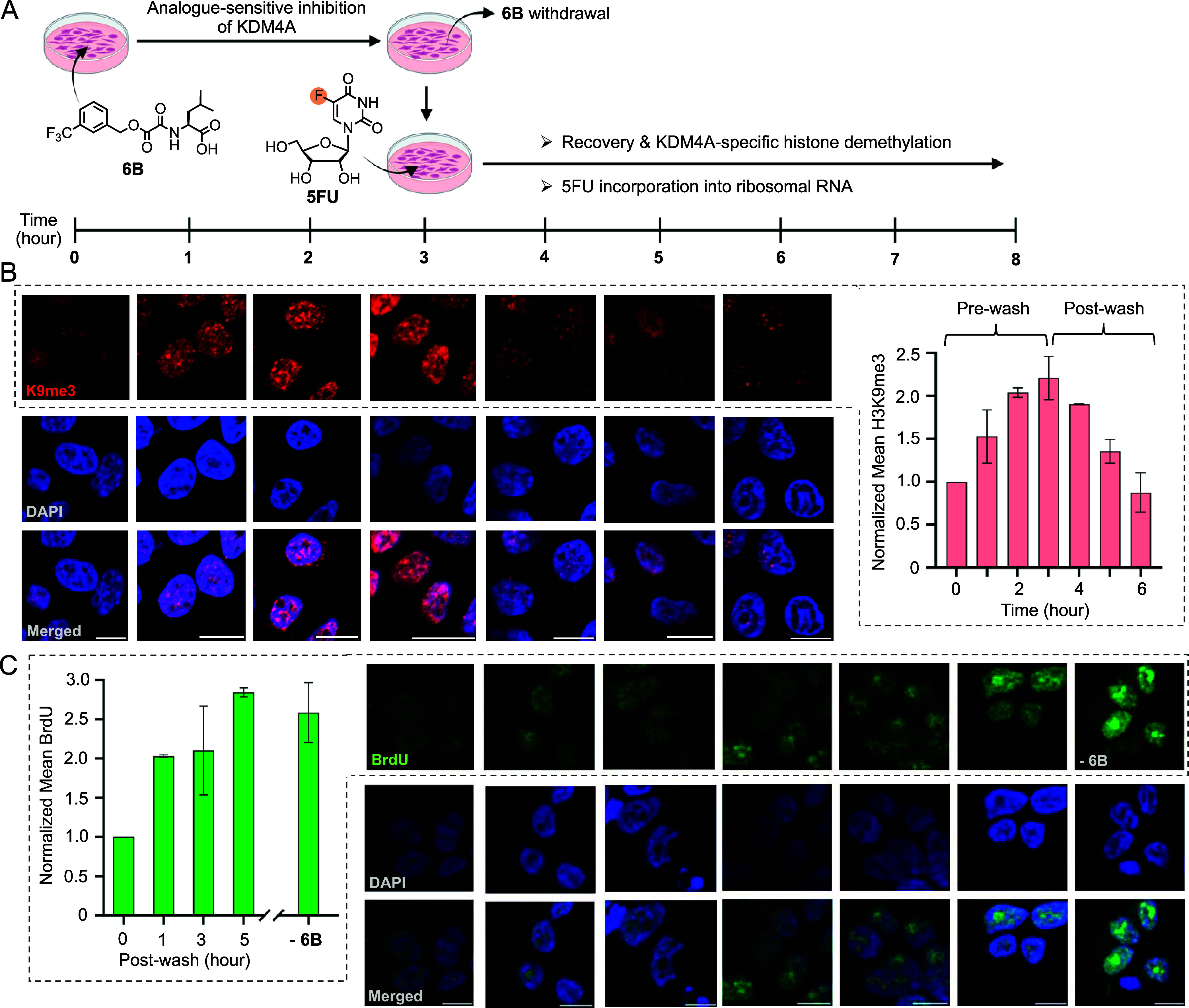
Temporal coordination between histone demethylation and rRNA transcription. (A) Schematic showing treatment of HEK293T cells (expressing KDM4A-F185A) with **6B** for 3 h followed by compound withdrawal and supplementing with 5FU. Cells were cultured in the presence of 5FU for up to 5 h. (B) Fixed cell immunofluorescence staining of time-dependent increase in H3K9me3 by **6B**. Compound withdrawal led to rapid H3K9me3 demethylation by the *as*KDM4A mutant. Bar diagram representation of temporal modulation of H3K9me3 during pre- and postwash of **6B**. (C) Withdrawal of **6B** led to a gradual increase in 5FU incorporation, a measure of rRNA transcription, as a result of *as*KDM4A-mediated histone demethylation (scale = 10 μm).

We exploited the reversible mode of action of the allele-specific inhibitor to gain further insights into the temporal regulation of rRNA expression by histone demethylation. We reasoned that withdrawal of the reversible inhibitor from the cell-culture medium would restore the activity of *as*KDM4A in a temporally defined manner (Figure 8A). HEK293T cells expressing the F185A variant carrying an HA tag were treated with **6B** and fixed at various time points. H3K9me3 levels were quantified in HA-positive cells to ensure that only cells with *as*KDM4A were considered for analysis. Within 3 h of compound treatment, a robust level of H3K9me3 ensued (Figure 8B). Subsequently, the same cells were allowed to grow in inhibitor-free media and examined for histone demethylation by fixed cell imaging. We noted that within 1 h of compound removal, *as*KDM4A regained its demethylase activity, which increased in a time-dependent manner for up to 3 h (Figure 8B), demonstrating a rapid restoration of catalytic activity upon release from the inhibitor block.

To investigate if the restoration of H3K9me3 demethylation is directly linked to rRNA activation, cells overexpressing *as*KDM4A were first treated with **6B** for 3 h followed by compound removal and brief exposure to 5FU before being fixed at different time points for immunofluorescence imaging (Figure 8A,C). As expected, incubation with **6B** resulted in a significant decrease in BrdU signal, indicating low transcriptional activity at the rDNA promoter, which is consistent with the high H3K9me3 level observed at this time point of inhibitor treatment (Figure 8B,C). We recorded a steady recovery of BrdU signal, reaching its maximum within 4–5 h postwithdrawal (equivalent to no **6B** treatment) (Figure 8C), consistent with reactivation of *as*KDM4A as observed above. A relatively sluggish 5FU incorporation into rRNA (Figure 8C), compared to the rapid histone demethylation (Figure 8B), is likely due to the time required to assemble active transcriptional machinery at the rRNA promoters following the removal of the silencing H3K9me3 mark. Achieving such precise temporal control of protein function would be challenging using genetic tools. Collectively, employing the mutant and cognate inhibitor pair, we mapped a highly dynamic signaling pathway linking KDM4A-mediated locus-specific histone demethylation with upregulation of rRNA expression to modulate downstream processes such as ribosomal assembly and protein synthesis.

## Conclusions

The diversity-based mechanism of protein evolution, in which multiple gene products arise from relatively fewer genes, leads to protein families with shared topological and biochemical features. This poses a significant challenge in accessing member-specific chemical probes, and achieving isoform selectivity with small-molecule inhibitors is exceedingly challenging. Members of the KDM family regulate mammalian gene expression and represent novel drug targets for multiple human diseases, such as cancer; yet compensatory and distinct functions of each KDM member are poorly defined. In this work, we employ an analogue-sensitive chemical genetic strategy to elucidate member-specific biological functions of KDMs in a temporally defined manner. We develop novel *as* variants of the KDM4 subfamily by replacing a conserved phenylalanine to alanine in the catalytic pocket and show the mutants exhibit biochemical traits similar to their wild type congeners. Our screening effort led to the identification of NOL as a potent allele-specific inhibitor to specifically perturb the mutants to conditionally modulate histone methylation in cultured human cells, confirming their reciprocal activity while remaining refractory to the endogenous demethylation machinery. We employ this orthogonal tool in conjunction with the BONCAT approach to decipher the member-specific role of KDM4 in dynamic cellular events and observe that KDM4A augments ribosomal protein synthesis; KDM4D lacking the protein–protein interaction domains, on the other hand, displays no such regulatory activity. Furthermore, exploiting the reversible mode of action of NOL, we uncover that KDM4A-mediated histone demethylation at the rDNA promoters leads to rapid rRNA expression, resulting in enhanced ribosomal assembly and protein synthesis. Given that KDM4A is overexpressed in multiple cancers, it is reasonable to speculate that KDM4A-mediated protein upregulation plays a critical role in tumorigenesis, which requires heightened ribosomal activity to support uncontrolled cellular proliferation. We anticipate future experiments involving the generation of *as*KDM4 knock-in line from mouse embryonic stem cells using CRISPR-Cas9 for precise manipulation of KDM4 members with NOL.^53,54^ Given that KDM4 knockout in mice results in embryonic lethality, perturbation of the silent *as*KDM4 variant with NOL would be a particularly attractive strategy to gain mechanistic insights into how histone demethylation regulates developmental processes *in vivo*. The engineering approach for developing a conditional demethylation apparatus is expected to be transferable to the human 2OG-dependent dioxygenase superfamily including RNA demethylases and prolyl hydroxylases for elucidation of their context-dependent biological functions.

## Supporting Information Available

Synthetic methods and reaction schemes for NOG analogues; experimental protocols for protein expression and purification; biochemical assays; and supporting figures and tables (PDF)
Histone extracts proteomic data (XLSX)
