# Supplementary material for: Analogue-Sensitive Inhibition of Histone Demethylases Uncovers Member-Specific Function in Ribosomal Protein Synthesis
Source: J Am Chem Soc. 2025 Jan 14;147(4):3341–52. doi: 10.1021/jacs.4c13870 (PMC11783601; doi:10.1021/jacs.4c13870)
Supplement: Supplementary file 1 — ja4c13870_si_001.pdf [file ja4c13870_si_001.pdf]

Analogue-Sensitive Inhibition of Histone Demethylases Uncovers Member-Specific Function in Ribosomal Protein Synthesis

Jordan Kuwik,[1#] Valerie Scott,[1#] Sara Chedid,[1] Stephanie Stransky,[2] Kathryn Hinkelman,[1] Sam Kavoosi,[1] Michael Calderon,[3] Simon Watkins,[3] Simone Sidoli,[2] and Kabirul Islam[1*]

[1]Department of Chemistry, University of Pittsburgh, Pittsburgh, PA, USA 15260; [2]Department of Biochemistry, Albert Einstein College of Medicine, Bronx, NY, USA 10461; [3]Department of Cell Biology, University of Pittsburgh, Pittsburgh, PA, USA 15260

[#]These authors contributed equally; [*]Corresponding author (kai27@pitt.edu)

Table of Content

1. General materials, methods, and equipment S2
2. Plasmids, mutagenic primers, cell lines and antibodies S2
3. Synthesis and characterization of NOG analogues **3-10** S3
4. Synthesis and characterization of cell-permeable NOG analogues **6A** and **6B** S4
5. Expression and purification of wild type KDM4A-E and analogue-sensitive mutants S5
6. Expression of a set of wild type 2OG enzymes S8
7. Expression and purification of Formaldehyde Dehydrogenase (FDH) S16
8. Demethylase activity of wild type and the mutant proteins on peptide substrate S16
9. Coupled fluorescence assay for measurement of kinetic parameters and IC50 values S17
10. In-vitro enzymatic activity of 2OG enzymes S18
11. Demethylase activity and inhibition of the KDM4 proteins on extracted histones S21
12. Examining cell permeability and toxicity of **6A** and **6B** S22
13. Expression of full-length KDM4A, B and D and their mutants in HEK293T cells S22
14. Expression and activity of full-length TET1 and 3 in HEK293T cells S23
15. Determining the effect of 6A/B on post-translational modifications in HEK293T cells S25
16. Incorporation of Azidohomoalanine in HEK293T cells S27
17. Analysis of rRNA expression in HEK293T cells using fluorescence in-situ hybridization S28
18. Analysis of rRNA promoter demethylation in HEK293T cells S29
19. Fixed cell immunofluorescence imaging S29
20. Quantification of fixed-cell imaging data S30
21. General protocol for Western blotting S30
22. Histone extraction, digestion, and tandem mass spectrometry S31
23. Supplementary figures and tables S34
24. References S65

1. General materials, methods, and equipment

Chemicals: All chemicals were purchased from established vendors (e.g., Sigma-Aldrich, Acros Organics) and used without purification unless otherwise noted. Optima grade acetonitrile was obtained from Fisher Scientific and degassed under vacuum prior to HPLC purification. All reactions to prepare N-oxalyl glycine (NOG) analogues were carried out in round bottom flasks and stirred with Teflon®-coated magnetic stir bars under inert atmosphere when needed following reported methods. Analytical thin layer chromatography (TLC) was performed using EMD 250 micron flexible aluminum backed, UV F_{254} pre-coated silica gel plates and visualized under UV light (254 nm) or by staining with phosphomolybdic acid, ninhydrin or anisaldehyde. Reaction solvents were removed by a Büchi rotary evaporator equipped with a dry ice-acetone condenser. Analytic and preparative HPLC was carried out on an Agilent 1220 Infinity HPLC with diode array detector. Concentration and lyophilization of aqueous samples were performed using Savant Sc210A SpeedVac Concentrator (Thermo), followed by Labconco or SP Scientific VirTis Benchtop Pro Freeze-Dryer system. Proton nuclear magnetic resonance spectra (^{1}H NMR) were recorded on Bruker Ultrashield™ Plus 600/500/400/300 MHz instruments at 24°C. Chemical shifts of ^{1}H and ^{13}C NMR spectra are reported as δ in units of parts per million (ppm) relative to tetramethylsilane (δ 0.0) or residual solvent signals: chloroform-d (δ 7.26, singlet), methanol-d_4 (δ 3.30, quintet), and deuterium oxide-d_2 (δ 4.80, singlet). Coupling constants are expressed in Hz. Mass spectra were collected on a Q-Exactive™ Thermo Scientific LC-MS with electron spray ionization (ESI) probe.

2. Plasmids, mutagenic primers, cell lines and antibodies

All the plasmids are either for bacterial or for mammalian expression. These plasmid constructs were obtained as gifts from individual laboratories or purchased from Addgene. Details of these constructs are given in Table S2. Mutagenic and ChIP-qPCR primers are obtained from Integrated DNA Technologies (Table S3, 4). Various competent bacterial cells were used for protein expression and mutagenesis. Human embryonic kidney 293T (HEK293T) cells, obtained from the American Type Culture Collection (ATCC) and used in the current study following manufacturer's protocol. All the antibodies used in the current study are purchased from established vendors and used following manufacturer's protocol (Table S5).

3. Synthesis and characterization of NOG analogues 3-10

Synthesis and characterization of the majority of the NOG analogues are described earlier.[1-4] Below, a brief description of synthesis of L-leucine NOG **6** and L-cyclohexyl NOG **10** are provided.

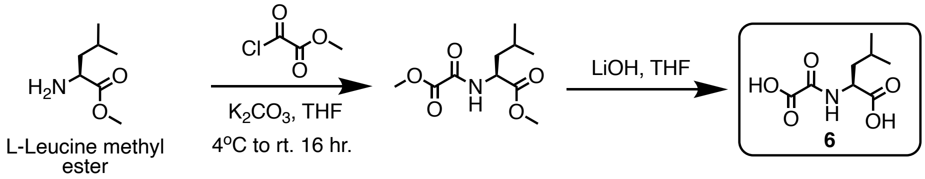

Scheme 1: Synthesis of NOG analogue **6**

To an ice-cold solution of L-leucine methyl ester hydrochloride (1.0 eq.) and K_2CO_3 (1.1 eq.) in anhydrous THF, was added methyl oxalyl chloride (2.2 eq.) and the reaction was stirred at rt overnight. The reaction mixture was concentrated under reduced pressure and the residue was purified by chromatography to give L-leucine NOG Ester which was characterized via ^{1}H and ^{13}C, and HRMS. L-leucine NOG ester was dissolved in 2 ml THF and 2 ml 1N LiOH and the reaction was stirred at room temperature overnight. THF was removed under reduced pressure and the residue was acidified to pH = 4 using 1N HCl, followed by HPLC purification to give L-leucine NOG. The NOG analogue was dissolved in deionized water, filtered through 0.2µM syringe filter, and purified with reversed-phase HPLC (XBridge™ BEH Prep C18 5µm OBD™ 10×250mm). The purification was monitored at 220 nm, eluting with acetonitrile (linear gradient to 80% in the first 12 minutes) in aqueous 0.1% trifluoro acetic acid at a flow rate of 4 mL/min. The peaks were concentrated by SpeedVac and further dried by lyophilization before complete characterization ^{1}H, ^{13}C, and HRMS. The product was dried and re-dissolved in deionized water and the concentrations were measured using standard curve generated for N-oxalyl glycine.

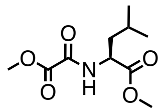

^{1}H NMR (300 MHz, MeOD): δ= 4.51-4.56 (m, 1H), 3.88 (s, 3H), 3.73 (s, 3H), 1.62-1.79 (m, 3H), 0.96 (d, J = 6.2 Hz, 3H), 0.93 (d, J = 6.0 Hz, 3H) ppm; ^{13}C (75 MHz, MeOD): δ= 173.54, 161.61, 159.14, 53.81, 52.89, 52.51, 40.92, 25.99, 23.24, 21.64 ppm.

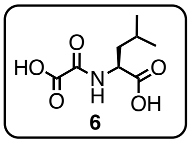

6

^{1}H NMR (300 MHz, D_2O): δ= 4.44 (dd, J = 4.8, 9.8 Hz, 1H), 1.59-1.81 (m, 3H), 0.90 (d, J = 6.2 Hz, 3H), 0.86 (d, J = 6.1 Hz, 3H) ppm; ^{13}C (75 MHz, D_2O): δ= 175.48, 161.86, 160.02, 51.71, 39.01, 24.33, 22.08, 20.39 ppm; $[\alpha]_D^{20} - 34.4\ (c1.4, \mathrm{H_2O})$.

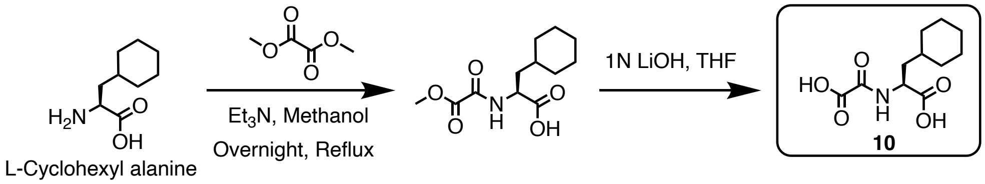

Scheme 2: Synthesis of NOG analogue **10**

To a reaction flask containing L-cyclohexyl alanine (1.0 eq.) in anhydrous methanol, was added dimethyl oxalate (2.0 eq.) and triethylamine (2.0 eq.) and the reaction was refluxed overnight. The reaction mixture was concentrated under reduced pressure and the residue was purified by chromatography to give L-cyclohexyl NOG Ester which was characterized via ^{1}H and ^{13}C, and HRMS. The intermediate NOG ester was then dissolved in 2 ml THF and 2 ml 1N LiOH at 0°C and the mixture was stirred at room temperature. After 1 hour, THF was evaporated, and the aqueous solution was acidified with 1N HCl to pH 4.0 and extracted with ethyl acetate. Compound was purified using HPLC following method listed for **6**.

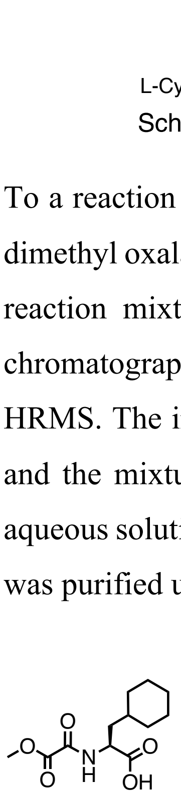

^{1}H NMR (500 MHz, $CDCl_3$): δ= 9.10-9.15 (s, 1H), 7.48 (d, J = 8.4 Hz, 1H), 4.62-4.67 (m, 1H), 3.91 (s, 3H). 1.77-1.82 (m, 2H), 1.62-1.70 (m, 5H), 1.33-1.36 (m, 1H), 1.11-1.26 (m, 3H), 0.86-1.00 (m, 2H); ^{13}C (125 MHz, $CDCl_3$): 176.74, 160.74, 156.43, 53.92, 50.81, 39.69, 34.17, 33.63, 32.35, 26.41, 26.19, 25.99 ppm; HRMS (ESI) calcd. for $C_{12}H_{20}O_5N$ [M+H^{+}]: 258.13360; found 258.13343.

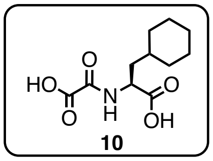

^{1}H NMR (300 MHz, DMSO): δ= 4.26 (dd, J = 4.4, 10.6 Hz, 1H), 1.52-1.72 (m, 7H), 1.07-1.27 (m, 4H), 0.75-0.95(m, 2H); ^{13}C (75 MHz, DMSO): δ= 173.36, 161.98, 158.7, 50.16, 37.7, 33.94, 33.38, 31.64, 26.29, 26.04, 25.85 ppm; HRMS (ESI) calcd for $C_{11}H_{16}O_5N$ [M-H^{+}]: 242.10230; found 242.10700.

4. Synthesis and characterization of cell-permeable NOG analogues 6A and 6B

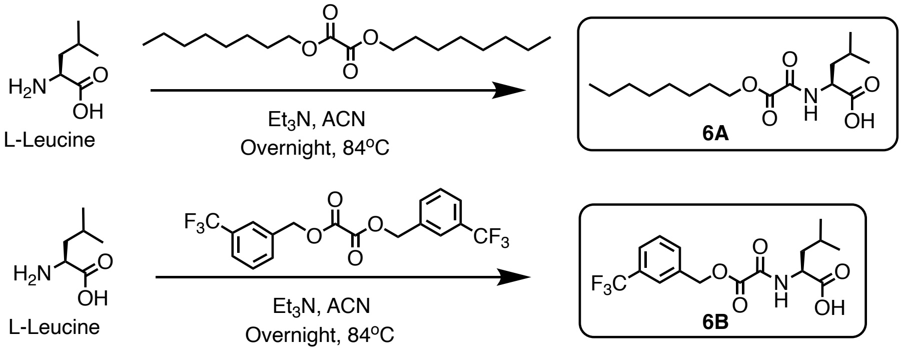

Scheme 3: Synthesis of cell-permeabe NOG analogue **6A** and **6B**

To a flask containing L-leucine in acetonitrile (1.0 eq.) was added di-octyl oxalate or di-trifluoromethyl benzyl oxalate (2.0 eq.) and triethylamine (2.0 eq.) and the reaction was refluxed overnight. The reaction mixture was washed with 1N HCl and the organic layer was extracted using ether. The organic layer was dried using magnesium sulfate, solvent was evaporated under reduced pressure, and the residue was purified by chromatography (typically, 1.0% to 10% MeOH in DCM) to yield **6A** and **6B** which were characterized via ^{1}H and ^{13}C, and HRMS.

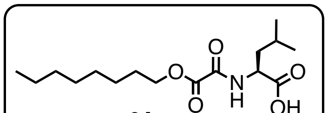

6A

^{1}H NMR (400 MHz, $CDCl_3$): δ= 9.92 (s, 1H), 7.45 (d, *J* = 8.6 Hz, 1H), 4.63-6.69 (m, 1H), 4.27 (t, *J* = 6.8 Hz, 2H), 1.65-1.79 (m, 5H), 1.26-1.37 (m, 11H), 0.96 (d, *J* = 5.9 Hz, 6H), 0.87 (t, *J* = 6.7 Hz, 3H); ^{13}C (100 MHz, $CDCl_3$): δ= 176.71, 160.31, 156.59, 67.71, 51.21, 41.24, 31.86, 29.24, 29.22, 28.39, 25.81, 24.98, 22.9, 22.73, 21.81, 14.19 ppm; HRMS (ESI) calcd. for $C_{16}H_{30}O_5N$ [M+H^{+}]: 316.21185; found 316.21170; $[\alpha]_D^{20} - 7.0\ (c1.5, \mathrm{CHCl_3})$.

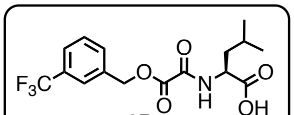

6B

^{1}H NMR (500 MHz, $CDCl_3$) δ= 7.40-7.68 (m, 4H), 5.35 (s, 2H), 4.64-4.68 (m, 1 H), 1.65-1.79 (m, 3H), 0.96 (d, *J* = 5.7 Hz, 1H); ^{13}C NMR (125 MHz, $CDCl_3$) δ= 176.35 171.58 160.07 156.05 135.23 132.25 131.49 131.23 129.50 125.98 125.95 125.92 125.90 125.74 125.71 68.06 60.67 51.28 41.25 25.03 22.91 21.85 21.23 14.36

5. Expression and purification of wild type KDM4A-E and analogue-sensitive mutants

The N-terminal 6xHis-tagged human KDM4A-jmjC domain (catalytic domain of KDM4A) bacterial expression construct pNIC28-Bsa4 (Addgene ID: 38846) was obtained from Addgene and expressed following reported method.[5] The wild type KDM4A plasmid was transformed into *E. Coli* BL21 (DE3) competent cells (Invitrogen) using pNIC28-Bsa4 kanamycin-resistant vector. A single colony was picked up and grown overnight at 37°C in 10 mL of Luria-Bertani (LB) broth in the presence of 50 µg/mL kanamycin. The culture was diluted 100-fold and allowed to grow at 37°C to an optical density (OD_{600}) of 0.8, and protein expression was induced overnight at 17°C with 0.6 mM IPTG in an Innova 44® Incubator shaker (New Brunswick Scientific). Proteins were purified as follows: harvested cells were resuspended in 15 mL lysis buffer (50 mM Tris-HCl pH 8.0, 200 mM NaCl, 5 mM *β*-mercaptoethanol (*β*-ME), 10% glycerol, 25 mM imidazole,

Lysozyme, DNase, and Roche protease inhibitor cocktail). The cells were lysed by pulsed sonication (Qsonica-Q700), and centrifuged at 13000 rpm for 40 min at 4°C. The soluble extracts were subject to Ni-NTA agarose resin (Thermo) according to manufacturer's instructions. After passing 20 volumes of washing buffer (50 mM Tris-HCl pH 8.0, 200 mM NaCl, 5 mM *β*-mercaptoethanol, 10% glycerol, and 25 mM imidazole), proteins were eluted with a buffer containing 50 mM Tris-HCl pH 8.0, 200 mM NaCl, 5 mM *β*-mercaptoethanol, 10% glycerol, and 400 mM imidazole. Proteins were further purified by size exclusion chromatography (Superdex-200) using AKTA pure FPLC system (GE healthcare) with buffer containing 50 mM Tris-HCl pH 8.0, 200 mM NaCl, and 10% glycerol. Purified proteins were concentrated using Amicon Ultra-10k centrifugal filter device (Merck Millipore Ltd.). The protein concentration was determined using Bradford assay kit (BioRad Laboratories) with BSA as a standard. The concentrated proteins were stored at -80°C before use.

The N-terminal strep-tagged human KDM4B and KDM4D jmjC catalytic domain for bacterial expression were kindly provided by Prof. Raymond Trievel, University of Michigan.[6,7] The native constructs were transformed into *E. coli* Rosetta [DE3] competent cells. A single colony was picked up and grown overnight at 37°C in 10 mL of Luria-Bertani (LB) broth in the presence of 100 μg/mL ampicillin. The culture was diluted 100-fold and allowed to grow at 37°C to an optical density (OD_{600}) of 0.8, and protein expression was induced overnight at 17°C with 1 mM IPTG in an Innova 44® Incubator shaker (New Brunswick Scientific). Proteins were purified as follows: harvested cells were resuspended in 15 mL lysis buffer (50 mM Tris-HCl pH 8.0, 200 mM NaCl, 5 mM *β*-mercaptoethanol, 10% glycerol, Lysozyme, DNase, and Roche protease inhibitor cocktail). The cells were lysed by pulsed sonication (Qsonica-Q700) and centrifuged at 13000 rpm for 40 min at 4 °C. The soluble extracts were subject to Strep-tactin resin (Qiagen, cat# 30004) according to manufacturer's instructions. After passing 20 volumes of washing buffer (50 mM Tris-HCl pH 8.0, 200 mM NaCl, 5 mM *β*-mercaptoethanol, 10% glycerol), proteins were eluted with a buffer containing 50 mM Tris-HCl pH 8.0, 200 mM NaCl, 5 mM *β*-mercaptoethanol, 10% glycerol, and 5 mM D-desthiobiotin. Proteins were further purified by size exclusion chromatography (Superdex-200) using AKTA pure FPLC system (GE healthcare) with buffer containing 50 mM Tris-HCl pH 8.0, 200 mM NaCl, and 10% glycerol. Purified proteins were concentrated using Amicon Ultra-10k centrifugal filter device (Merck Millipore Ltd.). The protein

concentration was determined using Bradford assay kit (BioRad Laboratories) with BSA as a standard. The concentrated proteins were stored at -80°C before use.

The N-terminal 6xHis-tagged human KDM4C catalytic domain construct for bacterial expression was a kind gift from Prof. Danica Fujimori, UCSF.[8] Plasmids were transformed into BL21 [DE3] star cells and grown at 37°C to an optical density of 0.6. Protein expression was induced with 0.3 mM IPTG overnight at 17°C. Following bacterial cell lysis, expressed protein was purified first by nickel affinity chromatography and secondly by size exclusion chromatography using a superdex 200 FPLC column. The proteins were concentrated via centrifugation. Protein concentration was determined using Bradford assay.

The N-terminal 6xHis-tagged KDM4E construct for bacterial expression was purchased from Addgene (#38990) and expressed following reported method.[9] The wild type KDM4E plasmid was transformed into *E. Coli* BL21 (DE3) competent cells (Invitrogen) using pNIC28-Bsa4 kanamycin-resistant vector. A single colony was grown overnight at 37°C in 10 mL of Luria-Bertani (LB) broth in the presence of 50 μg/mL kanamycin. The culture was diluted 100-fold and allowed to grow at 37°C to an optical density (OD_{600}) of 0.8, and protein expression was induced overnight at 17°C with 1 mM IPTG in an Innova 44® Incubator shaker (New Brunswick Scientific). KDM4E was purified from pelleted cells by resuspending in lysis buffer (50 mM Tris-HCl pH 8.0, 200 mM NaCl, 5 mM β-ME, 10% glycerol, 25 mM imidazole, lysozyme, DNase, and Pierce protease inhibitor cocktail). The cells were lysed by sonication at 60 mA, 10 seconds on followed by 10 seconds off, for a total of 2 minutes. The cell debris was pelleted at 13,000 rpm for 40 minutes. The soluble cell extracts were incubated with Ni-NTA agarose resin (Thermo) according to manufactures protocol. Beads were washed with 20 column volumes of was buffer (50 mM Tris-HCl pH 8.0, 200 mM NaCl, 10% glycerol 5 mM β-ME, and 25 mM imidazole). Proteins were eluted with 5 column volumes of elution buffer (50 mM Tris pH 8.0, 200 mM NaCl, 5 mM β-ME, 10% glycerol and 400 mM Imidazole). Proteins were next purified via size exclusion chromatography on AKTA pure FPLC system with 50 mM Tris pH 8.0, 200 mM NaCl, and 10% glycerol. Fractions with pure protein were combined and concentrated using Sartorius Vivaspin 10 kDa centrifugal concentrator. Bradford Dye (BioRad) was used to quantify the protein

concentration using BSA as a standard. The concentrated proteins were aliquoted, flash frozen, and stored at -80°C until use.

KDM4A-E variants (KDM4A-F185A, KDM4B-F186A, KDM4C-F187A, KDM4D-F189F, KDM4E-F186A) were generated using the QuikChange Lightning site-directed mutagenesis kit (Agilent Technologies). The primers are provided in Table S3. The resulting mutant plasmids were confirmed by DNA sequencing. The mutant proteins were expressed and purified as stated for the corresponding wildtype congeners.

6. Expression of a set of wild type 2OG enzymes

Expression and purification of MINA: MINA construct plasmid with N-terminal His Tev fusion tag was obtained from Addgene (# 39054). The expression was performed following a procedure from a previous publication.[10] The received bacterial stab was streaked on a 50 µg/mL kanamycin-treated agar plate and one colony was inoculated in 5mL of LB broth (Thermo Scientific) for 24 hours at 37°C. The bacterial culture was then centrifuged at 4000 rpm for 10 minutes and recombinant plasmid was purified using GeneJet miniprep kit (Thermo Scientific). 20ng of plasmid was then transformed into recombinant E. Coli BL21 (DE3) competent cells (Invitrogen) and grown in 50 µg/mL kanamycin-treated LB broth overnight at 37°C. One colony was inoculated into 10mL of LB broth in the presence of 50 µg/mL of kanamycin while shaking at 37°C for 16 hours. The inoculate was expressed on a large scale by pouring into a 1L broth of LB agar supplemented with 50 µg of kanamycin and shaken at 37°C in an Innova 44® Incubator shaker (New Brunswick Scientific) until an OD_{600} Of 0.7 was reached and 1mM of IPTG (Fisher Scientific BP1620-1) was added. The cultures were then shaken at 17°C for 16 hours in an Innova 44® Incubator shaker (New Brunswick Scientific). Cells were then pelleted by centrifugation at 4000 rpm for 20 minutes. The cell pellet was subject to lysis in a 15mL buffer containing 50 mM Tris-HCl pH 8.0, 200 mM NaCl, 5 mM *β*-ME, 10% glycerol, 25 mM imidazole, Lysozyme, DNase, and Pierce protease inhibitor cocktail. The resuspended cells were then lysed by pulsed sonication for 3 minutes on ice with 10 seconds on, 10 seconds off (Fisherbrand) followed by centrifugation at 13000 rpm for 40 minutes. Meanwhile, 750µL of Ni-NTA agarose resin (Thermo) were washed with 5mL of water followed by 5mL of wash buffer (50 mM Tris-HCl pH 8.0, 200

mM NaCl, 5 mM *b*-ME, 10% glycerol, and 25 mM imidazole). The supernatant of the centrifuged cells was then carefully decanted onto the Ni-NTA beads and allowed to rotate for 45 minutes at 4°C. The flow through was then allowed to pass through the column and 20 mL of wash buffer was added followed by 10mL of elution buffer (50 mM Tris-HCl pH 8.0, 200 mM NaCl, 5 mM b-ME, 10% glycerol, and 400 mM imidazole). Eluted protein was then further purified by AKTA pure FPLC system with buffer containing 50 mM Tris-HCl pH 8.0, 200 mM NaCl, and 10% glycerol. Purified proteins were concentrated using Sartorius Vivaspin-10k centrifugal filter device (Merck Millipore Ltd.). The concentration of protein was then determined by Bradford assay (Biorad) kit with BSA concentrations as a standard. Protein was aliquoted and stored in -80C until use.

Expression and purification of C14orf169 (NO66): C14orf169 construct plasmid with C-terminal Tev His Flag fusion tag was obtained from Addgene (# 39114). The expression was performed following a procedure from a previous publication.[11] The received bacterial stab was streaked on a 50 µg/mL kanamycin-treated agar plate and one colony was inoculated in 5mL of LB broth (Thermo Scientific) for 24 hours at 37°C. The bacterial culture was then centrifuged at 4000 rpm for 10 minutes and recombinant plasmid was purified using GeneJet miniprep kit (Thermo Scientific). 20ng of the pNIC-CTHF plasmid was then transformed into recombinant E. Coli BL21 (DE3) competent cells (Invitrogen) and grown in 50 µg/mL kanamycin-treated LB agar overnight at 37°C. One colony was inoculated into 10mL of LB broth in the presence of 50 µg/mL of kanamycin while shaking at 37°C for 16 hours. The inoculate was expressed on a large scale by pouring into a 1L broth of LB agar supplemented with 50 µg of kanamycin and shaken at 37°C in an Innova 44® Incubator shaker (New Brunswick Scientific) until an OD_{600} Of 0.7 was reached and 1mM of IPTG (Fisher Scientific BP1620-1) was added. The cultures were then shaken at 17°C for 16 hours in an Innova 44® Incubator shaker (New Brunswick Scientific). Cells were then pelleted by centrifugation at 4000 rpm for 20 minutes. The cell pellet was subject to lysis in a 15mL buffer containing 50 mM Tris-HCl pH 8.0, 200 mM NaCl, 5 mM *b*-ME, 10% glycerol, 25 mM imidazole, Lysozyme, DNase, and Pierce protease inhibitor cocktail. The resuspended cells were then lysed by pulsed sonication for 3 minutes on ice with 10 seconds on, 10 seconds off (Fisherbrand) followed by centrifugation at 13000 rpm for 40 minutes. Meanwhile, 750µL of Ni-

NTA agarose resin (Thermo) were washed with 5mL of water followed by 5mL of wash buffer (50 mM Tris-HCl pH 8.0, 200 mM NaCl, 5 mM *β*-ME, 10% glycerol, and 25 mM imidazole). The supernatant of the centrifuged cells was then carefully decanted onto the Ni-NTA beads and allowed to rotate for 45 minutes at 4°C. The flow through was then allowed to pass through the column and 20 mL of wash buffer was added followed by 10mL of elution buffer (50 mM Tris-HCl pH 8.0, 200 mM NaCl, 5 mM *β*-ME, 10% glycerol, and 400 mM imidazole). Eluted protein was then further purified by AKTA pure FPLC system with buffer containing 50 mM Tris-HCl pH 8.0, 200 mM NaCl, and 10% glycerol. Purified proteins were concentrated using Sartorius Vivaspin-10k centrifugal filter device (Merck Millipore Ltd.). The concentration of protein was then determined by Bradford assay (Bioad) kit with BSA concentrations as a standard. Protein was aliquoted and stored in -80C until use.

Expression and purification of EGLN1 (PHD2): Full length EGLN1 construct plasmid with N-terminal Tev His fusion tag was designed and ordered from Vector Builder. EGLN1 was purified utilizing a method published previously.[12] 20ng of the pET28 plasmid was then transformed into recombinant E. Coli BL21 (DE3) competent cells (Invitrogen) and grown on 100 µg/mL-ampicillin treated agar overnight at 37°C. One colony was inoculated into 10mL of LB broth in the presence of 100 µg/mL of ampicillin while shaking at 37°C for 16 hours. The inoculate was expressed on a large scale by pouring into a 1L broth of LB agar supplemented with 100 µg of ampicillin and shaken at 37°C in an Innova 44® Incubator shaker (New Brunswick Scientific) until an OD_{600} Of 0.7 was reached and 1mM of IPTG (Fisher Scientific BP1620-1) was added. The cultures were then shaken at 17°C for 16 hours in an Innova 44® Incubator shaker (New Brunswick Scientific). Cells were then pelleted by centrifugation at 4000 rpm for 20 minutes. The cell pellet was subject to lysis in a 15mL buffer containing 50 mM Tris-HCl pH 8.0, 200 mM NaCl, 5 mM *β*-ME, 10% glycerol, 25 mM imidazole, Lysozyme, DNase, and Pierce protease inhibitor cocktail. The resuspended cells were then lysed by pulsed sonication for 3 minutes on ice with 10 seconds on, 10 seconds off (Fisherbrand) followed by centrifugation at 13000 rpm for 40 minutes. Meanwhile, 750µL of Ni-NTA agarose resin (Thermo) were washed with 5mL of water followed by 5mL of wash buffer (50 mM Tris-HCl pH 8.0, 200 mM NaCl, 5 mM *β*-ME, 10% glycerol, and 25 mM imidazole). The supernatant of the centrifuged cells was then carefully

decanted onto the Ni-NTA beads and allowed to rotate for 45 minutes at 4°C. The flow through was then allowed to pass through the column and 20 mL of wash buffer was added followed by 10mL of elution buffer (50 mM Tris-HCl pH 8.0, 200 mM NaCl, 5 mM b-ME, 10% glycerol, and 400 mM imidazole). Eluted protein was then further purified by AKTA pure FPLC system with buffer containing 50 mM Tris-HCl pH 8.0, 200 mM NaCl, and 10% glycerol. Purified proteins were concentrated using Sartorius Vivaspin-10k centrifugal filter device (Merck Millipore Ltd.). The concentration of protein was then determined by Bradford assay (Biorad) kit with BSA concentrations as a standard. Protein was aliquoted and stored in -80C until use.

Expression and purification of EGLN3 (PHD3): Full length EGLN3 construct plasmid with N-terminal Tev His fusion tag was designed and ordered from Vector Builder. EGLN3 was purified utilizing a method published previously.[12] 20ng of the pET28 plasmid was then transformed into recombinant E. Coli BL21 (DE3) competent cells (Invitrogen) and grown on 100 µg/mL-ampicillin treated agar overnight at 37°C. One colony was inoculated into 10mL of LB broth in the presence of 100 µg/mL of ampicillin while shaking at 37°C for 16 hours. The inoculate was expressed on a large scale by pouring into a 1L broth of LB agar supplemented with 100 µg of ampicillin and shaken at 37°C in an Innova 44® Incubator shaker (New Brunswick Scientific) until an OD_{600} Of 0.7 was reached and 1mM of IPTG (Fisher Scientific BP1620-1) was added. The cultures were then shaken at 17°C for 16 hours in an Innova 44® Incubator shaker (New Brunswick Scientific). Cells were then pelleted by centrifugation at 4000 rpm for 20 minutes. The cell pellet was subject to lysis in a 15mL buffer containing 50 mM Tris-HCl pH 8.0, 200 mM NaCl, 5 mM *β*-ME, 10% glycerol, 25 mM imidazole, Lysozyme, DNase, and Pierce protease inhibitor cocktail. The resuspended cells were then lysed by pulsed sonication for 3 minutes on ice with 10 seconds on, 10 seconds off (Fisherbrand) followed by centrifugation at 13000 rpm for 40 minutes. Meanwhile, 750µL of Ni-NTA agarose resin (Thermo) were washed with 5mL of water followed by 5mL of wash buffer (50 mM Tris-HCl pH 8.0, 200 mM NaCl, 5 mM *b*-ME, 10% glycerol, and 25 mM imidazole). The supernatant of the centrifuged cells was then carefully decanted onto the Ni-NTA beads and allowed to rotate for 45 minutes at 4°C. The flow through was then allowed to pass through the column and 20 mL of wash buffer was added followed by 10mL of elution buffer (50 mM Tris-HCl pH 8.0, 200 mM NaCl, 5 mM *β*-ME, 10% glycerol, and

400 mM imidazole). Eluted protein was then further purified by AKTA pure FPLC system with buffer containing 50 mM Tris-HCl pH 8.0, 200 mM NaCl, and 10% glycerol. Purified proteins were concentrated using Sartorius Vivaspin-10k centrifugal filter device (Merck Millipore Ltd.). The concentration of protein was then determined by Bradford assay (BioRad) kit with BSA concentrations as a standard. Protein was aliquoted and stored in -80C until use.

Expression and purification of KDM6B: The plasmid construct for the catalytic domain of wild type KDM6B was kindly provided by Prof. Christopher Schofield of the University of Oxford. The protein was purified as reported by previous publications.[13] Purified plasmid was transformed into Rosetta [DE3] competent cells. Propagation of plasmid-containing cells was performed in Luria-Bertani (LB) broth at 37 °C. Once the optical density reached ~0.8, protein expression was induced with 0.3 mM IPTG shaking at 17°C overnight. The cells were collected by centrifugation at 4000 rpm for 30 minutes. Following bacterial cell lysis, expressed protein was purified first by nickel affinity chromatography and secondly by size exclusion chromatography using a superdex 200 FPLC column.

Expression and purification of ALKBH2: ALKBH2 construct was a kind gift from Dr. Chuan He (University of Chicago). Purification of the protein was performed closely following a previously reported method.[14] 20ng ofALKBH2 plasmid with His tag in a pET28a vector was transformed into recombinant E. Coli BL21 (DE3) competent cells (Invitrogen) and grown on 50 µg/mL kanamycin-treated agar overnight at 37°C. One colony was inoculated into 10mL of LB broth in the presence of 50 µg/mL of kanamycin while shaking at 37°C for 16 hours. The inoculate was expressed on a large scale by pouring into a 1L broth of LB agar supplemented with 50 µg of kanamycin and shaken at 37°C in an Innova 44® Incubator shaker (New Brunswick Scientific) until an OD_{600} Of 0.7 was reached and 1mM of IPTG (Fisher Scientific BP1620-1) was added. The cultures were then shaken at 17°C for 16 hours in an Innova 44® Incubator shaker (New Brunswick Scientific). Cells were then pelleted by centrifugation at 4000 rpm for 20 minutes. The cell pellet was subject to lysis in a 15mL buffer containing 50 mM Tris-HCl pH 8.0, 200 mM NaCl, 5 mM *β*-ME, 10% glycerol, 25 mM imidazole, Lysozyme, DNase, and Pierce protease inhibitor cocktail. The resuspended cells were then lysed by pulsed sonication for 3 minutes on ice

with 10 seconds on, 10 seconds off (Fisherbrand) followed by centrifugation at 13000 rpm for 40 minutes. Meanwhile, 750µL of Ni-NTA agarose resin (Thermo) were washed with 5mL of water followed by 5mL of wash buffer (50 mM Tris-HCl pH 8.0, 200 mM NaCl, 5 mM *β*-ME, 10% glycerol, and 25 mM imidazole). The supernatant of the centrifuged cells was then carefully decanted onto the Ni-NTA beads and allowed to rotate for 45 minutes at 4°C. The flow through was then allowed to pass through the column and 20 mL of wash buffer was added followed by 10mL of elution buffer (50 mM Tris-HCl pH 8.0, 200 mM NaCl, 5 mM *β*-ME, 10% glycerol, and 400 mM imidazole). Eluted protein was then further purified by AKTA pure FPLC system with buffer containing 50 mM Tris-HCl pH 8.0, 200 mM NaCl, and 10% glycerol. Purified proteins were concentrated using Sartorius Vivaspin-10k centrifugal filter device (Merck Millipore Ltd.). The concentration of protein was then determined by Bradford assay (Biorad) kit with BSA concentrations as a standard. Protein was aliquoted and stored in -80C until use.

Expression and purification of ALKBH5: ALKBH5 construct was a kind gift from Dr. Chuan He (University of Chicago). 20ng ofALKBH5 plasmid with C-terminal His tag in a PMCSG19 vector was then transformed into recombinant E. Coli BL21 (DE3) competent cells (Invitrogen) and grown on 50 µg/mL kanamycin-treated agar overnight at 37°C. One colony was inoculated into 10mL of LB broth in the presence of 50 µg/mL of kanamycin while shaking at 37°C for 16 hours. The inoculate was expressed on a large scale by pouring into a 1L broth of LB agar supplemented with 50 µg of kanamycin and shaken at 37°C in an Innova 44® Incubator shaker (New Brunswick Scientific) until an OD_{600} Of 0.7 was reached and 1mM of IPTG (Fisher Scientific BP1620-1) was added. The cultures were then shaken at 17°C for 16 hours in an Innova 44® Incubator shaker (New Brunswick Scientific). Cells were then pelleted by centrifugation at 4000 rpm for 20 minutes. The cell pellet was subject to lysis in a 15mL buffer containing 50 mM Tris-HCl pH 8.0, 200 mM NaCl, 5 mM *β*-ME, 10% glycerol, 25 mM imidazole, Lysozyme, DNase, and Pierce protease inhibitor cocktail. The resuspended cells were then lysed by pulsed sonication for 3 minutes on ice with 10 seconds on, 10 seconds off (Fisherbrand) followed by centrifugation at 13000 rpm for 40 minutes. Meanwhile, 750µL of Ni-NTA agarose resin (Thermo) were washed with 5mL of water followed by 5mL of wash buffer (50 mM Tris-HCl pH 8.0, 200 mM NaCl, 5 mM *b*-ME, 10% glycerol, and 25 mM imidazole). The supernatant of the centrifuged cells was

then carefully decanted onto the Ni-NTA beads and allowed to rotate for 45 minutes at 4°C. The flow through was then allowed to pass through the column and 20 mL of wash buffer was added followed by 10mL of elution buffer (50 mM Tris-HCl pH 8.0, 200 mM NaCl, 5 mM *β*-ME, 10% glycerol, and 400 mM imidazole). Eluted protein was then further purified by AKTA pure FPLC system with buffer containing 50 mM Tris-HCl pH 8.0, 200 mM NaCl, and 10% glycerol. Purified proteins were concentrated using Sartorius Vivaspin-10k centrifugal filter device (Merck Millipore Ltd.). The concentration of protein was then determined by Bradford assay (Biorad) kit with BSA concentrations as a standard. Protein was aliquoted and stored in -80C until use.

Expression and purification of ALKBH9 (FTO): Expression of FTO was performed closely following a reported method.[15] Briefly, 20ng of FTO plasmid with N-terminal His tag in a pET28a vector was then transformed into recombinant E. Coli BL21 (DE3) competent cells (Invitrogen) and grown on 50 µg/mL kanamycin-treated agar overnight at 37°C. One colony was inoculated into 10mL of LB broth in the presence of 50 µg/mL of kanamycin while shaking at 37°C for 16 hours. The inoculate was expressed on a large scale by pouring into a 1L broth of LB agar supplemented with 50 µg of kanamycin and shaken at 37°C in an Innova 44® Incubator shaker (New Brunswick Scientific) until an OD_{600} Of 0.7 was reached and 1mM of IPTG (Fisher Scientific BP1620-1) was added. The cultures were then shaken at 17°C for 16 hours in an Innova 44® Incubator shaker (New Brunswick Scientific). Cells were then pelleted by centrifugation at 4000 rpm for 20 minutes. The cell pellet was subject to lysis in a 15mL buffer containing 50 mM Tris-HCl pH 8.0, 200 mM NaCl, 5 mM *β*–ME, 10% glycerol, 25 mM imidazole, Lysozyme, DNase, and Pierce protease inhibitor cocktail. The resuspended cells were then lysed by pulsed sonication for 3 minutes on ice with 10 seconds on, 10 seconds off (Fisherbrand) followed by centrifugation at 13000 rpm for 40 minutes. Meanwhile, 750µL of Ni-NTA agarose resin (Thermo) were washed with 5mL of water followed by 5mL of wash buffer (50 mM Tris-HCl pH 8.0, 200 mM NaCl, 5 mM *β*-ME, 10% glycerol, and 25 mM imidazole). The supernatant of the centrifuged cells was then carefully decanted onto the Ni-NTA beads and allowed to rotate for 45 minutes at 4°C. The flow through was then allowed to pass through the column and 20 mL of wash buffer was added followed by 10mL of elution buffer (50 mM Tris-HCl pH 8.0, 200 mM NaCl, 5 mM *β*-ME, 10% glycerol, and 400 mM imidazole). Eluted protein was then further purified by AKTA

pure FPLC system with buffer containing 50 mM Tris-HCl pH 8.0, 200 mM NaCl, and 10% glycerol. Purified proteins were concentrated using Sartorius Vivaspin-10k centrifugal filter device (Merck Millipore Ltd.). The concentration of protein was then determined by Bradford assay (Biorad) kit with BSA concentrations as a standard. Protein was aliquoted and stored in -80C until use.

Expression and purification of TET2: Recombinant TET2 Wild Type plasmid was a kind gift from Dr. Yanhui Xu, Fudan University. 20ng of the TET2 plasmid with N-terminal His tag in a pET28b vector was transformed into recombinant *E. Coli* BL21 (DE3) competent cells (Invitrogen) and grown on 50 µg/mL kanamycin-treated agar overnight at 37°C. One colony was inoculated into 10mL of LB broth in the presence of 50 µg/mL of kanamycin while shaking at 37°C for 16 hours. The inoculate was expressed on a large scale by pouring into a 1L broth of LB agar supplemented with 50 µg of kanamycin and shaken at 37°C in an Innova 44® Incubator shaker (New Brunswick Scientific) until an OD_{600} of 0.7 was reached and 1mM of IPTG (Fisher Scientific BP1620-1) was added. The cultures were then shaken at 17°C for 16 hours in an Innova 44® Incubator shaker (New Brunswick Scientific). Cells were then pelleted by centrifugation at 4000 rpm for 20 minutes. The cell pellet was subject to lysis in a 15mL buffer containing 50 mM Tris-HCl pH 8.0, 200 mM NaCl, 5 mM *β*-ME, 10% glycerol, 25 mM imidazole, Lysozyme, DNase, and Pierce protease inhibitor cocktail. The resuspended cells were then lysed by pulsed sonication for 3 minutes on ice with 10 seconds on, 10 seconds off (Fisherbrand) followed by centrifugation at 13000 rpm for 40 minutes. Meanwhile, 750µL of Ni-NTA agarose resin (Thermo) were washed with 5mL of water followed by 5mL of wash buffer (50 mM Tris-HCl pH 8.0, 200 mM NaCl, 5 mM *β*-ME, 10% glycerol, and 25 mM imidazole). The supernatant of the centrifuged cells was then carefully decanted onto the Ni-NTA beads and allowed to rotate for 45 minutes at 4°C. The flow through was then allowed to pass through the column and 20 mL of wash buffer was added followed by 10mL of elution buffer (50 mM Tris-HCl pH 8.0, 200 mM NaCl, 5 mM *β*-ME, 10% glycerol, and 400 mM imidazole). Eluted protein was then further purified by AKTA pure FPLC system with buffer containing 50 mM Tris-HCl pH 8.0, 200 mM NaCl, and 10% glycerol. Purified proteins were concentrated using Sartorius Vivaspin-10k centrifugal filter device (Merck Millipore Ltd.). The concentration of protein was then determined by Bradford

assay (Biorad) kit with BSA concentrations as a standard. Protein was aliquoted and stored in -80C until use.

7. Expression and purification of Formaldehyde Dehydrogenase (FDH)

The N-terminal 6xHis-tagged *P. putida* FDH bacterial expression construct in pET28 vector was obtained from the Bhagwat laboratory at the Wayne State University.[16] The plasmid was transformed into *E. coli* Rosetta [DE3] competent cells. A single colony was picked up and grown overnight at 37°C in 10 mL of Luria-Bertani (LB) broth in the presence of 50 µg/mL kanamycin. The culture was diluted 100-fold and allowed to grow at 37°C to an optical density (OD_{600}) of 0.8, and protein expression was induced overnight at 17°C with 1 mM IPTG in an Innova 44® Incubator shaker (New Brunswick Scientific). Proteins were purified as follows: harvested cells were resuspended in 15 mL lysis buffer (50 mM Tris-HCl pH 8.0, 200 mM NaCl, 5 mM *β*-mercaptoethanol, 10% glycerol, 25 mM imidazole, Lysozyme, DNase, and Roche protease inhibitor cocktail). The cells were lysed by pulsed sonication (Qsonica-Q700), and centrifuged at 13000 rpm for 40 min at 4 °C. The soluble extracts were subject to Ni-NTA agarose resin (Thermo) according to manufacturer's instructions. After passing 20 volumes of washing buffer (50 mM Tris-HCl pH 8.0, 200 mM NaCl, 5 mM *β*-mercaptoethanol, 10% glycerol, and 25 mM imidazole), proteins were eluted with a buffer containing 50 mM Tris-HCl pH 8.0, 200 mM NaCl, 5 mM *β*-mercaptoethanol, 10% glycerol, and 400 mM imidazole. Proteins were further purified by size exclusion chromatography (Superdex-200) using AKTA pure FPLC system (GE healthcare) with buffer containing 50 mM Tris-HCl pH 8.0, 200 mM NaCl, and 10% glycerol. Purified proteins were concentrated using Amicon Ultra-10k centrifugal filter device (Merck Millipore Ltd.). The protein concentration was determined using Bradford assay kit (BioRad Laboratories) with BSA as a standard. The concentrated proteins were stored at -80°C before use.

8. Demethylase activity of the wild type and mutant KDM4 proteins on peptide substrate

To monitor enzymatic activity, a demethylase activity assay was optimized and then observed through MALDI-TOF.[7] Each histone demethylase assay sample included either wild type or mutant enzyme (10µM KDM4A, 7.5µM KDM4C, 10µM KDM4D, or 1µM KDM4E), 10 µM peptide, 200 µM 2OG, 50 mM Tris pH 8, 50 mM $(NH_4)_2Fe(SO_4)_2$, 0-200µM NOG analogue, and 2 mM L-ascorbic acid with a total assay volume of 10 µL. Fe(II) supplement and L-ascorbic acid

were prepared freshly. An enzyme master-mix containing everything except 2OG and Peptide was aliquoted and incubated with inhibitor for 10 minutes on ice. Lastly, the 2OG and peptide were added to the enzyme mix and the samples were incubated at 37°C for 30 minutes. To observe demethylase activity, 1 μL of assay sample was applied to the MALDI plate followed by 1 μL of CHCA or S-DHB matrix. The sample was ionized with Bruker FlexControl in the reflectron positive mode and analyzed on Bruker FlexAnalysis software. The percent demethylated peptide was calculated by first multiplying the K9me1 peak by 2 and the K9me0 peak by 3 to account for the multiple demethylation events on the peptide. The percent peak area was calculated, and the K9me2, me1, and me0 peaks were summed to get the relative percent demethylated peptide**.** The negative control included all components of the assay except for a KDM4 protein.

9. Coupled fluorescence assay for measurement of kinetic parameters and IC_{50} values

To determine the catalytic efficiency of the wild type and engineered KDM4-2OG pairs, the demethylase activity was measure by fluorescence intensity of accumulated NADH by employing a coupled fluorescence intensity assay.[16,7] The experiments were performed in 384-well white Corning plates. The assay was composed of an enzyme cocktail (50 mM HEPES pH 8.0, 50 μM $(NH_4)_2Fe(SO_4)_2$, 1 mM L-ascorbic acid, 1 mM NAD+, 200 nM FDH, and 1 μM KDM4A or KDM4A-F185A, 2 μM KDM4C WT or 3 μM KDM4C-F187A, 3 μM KDM4D WT or KDM4D-F189A, 3 μM KDM4E WT or KDM4E-F186A and a substrate cocktail (0-1500 μM 2KG/analogue and 300 μM $H3K9Me_3$ peptide). The substrate cocktail was first applied to the microplate followed by the enzyme cocktail. The accumulation of NADH was measured by fluorescence intensity (excitation= 340 nm, emission= 490 nm) every 30 seconds over a period of 15 minutes or until the linear range of turnover was abrogated on a TECAN Infinite M1000Pro. The data was analyzed using GraphPad Prism software and converted to μM of H3K9me3 demethylated using the NADH calibration curve. Only points within the linear range were used to calculate the slope for each 2OG concentration and the values were fitted to the Michaelis-Menten equation to get the K_M and k_{cat} values (Y = Et*kcat*X/(Km + X); Y = enzyme velocity, Et = concentration of enzyme catalytic sites, X = substrate concentration). Experiments were performed in triplicates.

To convert the relative fluorescence unit (RFU) values collected in the coupled fluorescence intensity assay, an NADH calibration curve was generated by measuring fluorescence intensity.[2]

NADH (ACROS, cat #124530050) was added to assay buffer (50 mM HEPES pH 8, 50 μM $(NH_4)_2Fe(SO_4)_2$, 1 mM L-ascorbic acid, 1 mM NAD+, 200 nM FDH, and 1 μM KDM4A) in varying concentrations (0-10 μM). The experiments were performed in 384-well white Corning plates. The fluorescence intensity was measured with excitation = 340 nm and emission = 490 nm on a TECAN Infinite M1000Pro. Triplicate values were taken for each concentration.

The set up and analysis for IC_{50} of L-Leu NOG against KDM4A F185A enzyme was similar to that described in the coupled fluorescence assay section, but adjusted to function as an end point assay. The assay composed of an enzyme cocktail (50mM HEPES pH 8.0, 75 μM $(NH_4)_2Fe(SO_4)_2$, 2 mM L-ascorbic acid, 1 mM NAD^+, 200 nM FDH, 3 μM wild type KDM4A or KDM4A-F185A, and varying concentration of compounds (NOGs **6**, **9** and **10**) and a substrate cocktail (200 μM 2OG and 60 μM H3K9me3 peptide). Different concentrations of NOG **6** were measured at one time point ranging for 1-5 hours. For **9** and **10**, the same set-up remained, but enzymatic activity was measured via MALDI-TOF analysis, and relative activity and inhibition was calculated for different NOG concentrations as described above. IC_{50} measurements were performed in triplicates. The data was plotted in GraphPad Prism to the equation Y=Bottom + (Top-Bottom)/(1+10^(X-LogIC50)), where Y = % Activity, and Bottom and Top being the lower and upper most plateaus of percent activity, respectively.

10. In-vitro enzymatic activity of 2OG enzymes

MALDI-based Assay for KDM6B: To monitor the enzymatic activity of KDM6B WT in the presence of L-Leu NOG **6**, we utilized a MALDI-TOF based assay.[17] 10μM WT enzyme was incubated with 10 μM H3K27me3 peptide (H_2N-APRKQLATKAARK(me3)SAPATGGVK-$CONH_2$), 50 mM Tris pH 8.0, 75 μM Fe^{2+}, 50 mM Ascorbic Acid, 50-200 μM L-Leu NOG **6** or equivalent volume of water in 10 μL aliquots. The samples were left to sit on ice for 10 minutes followed by addition of 200μM 2-KG and samples were placed at 37°C. At various time points of 5-30 minutes, 1 μL of each sample was spotted on a MALDI plate with addition of 1 μL of CHCA or S-DHB matrix. The sample was ionized with Bruker FlexControl in the reflectron positive mode and analyzed on Bruker FlexAnalysis software. The percent demethylated peptide was calculated by first multiplying the K27me1 peak by 2 and the K27me0 peak by 3 to account for the multiple

demethylation events on the peptide. The percent peak area was calculated, and the K27me2, me1, and me0 peaks were summed to get the relative percent demethylated peptide**.**

MALDI-based assay for FTO and ALKBH5: To determine the enzymatic activity of FTO and ALKBH5 recombinant proteins in the presence of L-Leu NOG **6**, a previously published methods were utilized.[15, 18] Briefly,10 µM of each protein was incubated in the presence of 10 µM RNA substrate (5'-CUGGm6ACUGG-3'), 75 µM Iron (II), 2mM Ascorbic Acid, 50 mM HEPES pH 7.5, 150 mM KCl, 50 µM L-Leu NOG or equivalent volume of water in 10 µL aliquots. The samples were placed on ice for 10 minutes followed by addition of 200 µM 2OG or equivalent volume of water. After incubation at 37°C for 30 minutes, samples were desalted with 3 µL of BT AG 50W-X8 RESIN (Bio-Rad 143-5441) at room temperature for 10 minutes. Samples were then spotted on a MALDI plate with 1uL sample and 1uL of 3-HPA matrix and monitored with Bruker FlexControl in the reflectron negative mode and analyzed on Bruker FlexAnalysis software. Relative inhibition was monitored via assessing percent product decrease with L-Leu NOG samples compared to no L-Leu NOG samples.

MALDI-based assay for C14orf169 (NO66) and MINA: To monitor the enzymatic activity of C14orf169 and MINA recombinant proteins, previous methods were utilized.[10-11] Briefly, 10 µM each recombinant protein was incubated with 10 µM peptide substrate (NH_2-PVEHPFGGGN**H**QHIGKPSTIR-$CONH_2$ for C14orf169 and NH_2-PGGRGNAGGL**H**HHRINFDKYH-$CONH_2$ for MINA), 300mM NaCl, 500 µM TCEP, 200 µM Ascorbic Acid, 100 µM Iron (II), 50mM HEPES pH 7.5, and 200 µM L-Leu NOG or equivalent volume of water in 10 µL water. Aliquots were kept on ice for 10 minutes before addition of 200 µM 2OG or equivalent volume of water. After 30-60 minutes incubation at 37°C, 1 µL sample and 1 µL S-DHB matrix were spotted on a MALDI plate and monitored with Bruker FlexControl in the reflectron positive mode and analyzed on Bruker FlexAnalysis software. Relative inhibition was monitored via assessing percent product decrease with L-Leu NOG samples compared to no L-Leu NOG samples.

MALDI-based assay EGLN1 and EGLN3: **The activity of EGLN1 and EGLN3 were monitored following a reported method.[19]** To monitor the enzymatic activity of EGLN1 and 3

recombinant proteins, 10 μM of each recombinant protein was incubated with 10 μM peptide substrate (NH_2-DLDLEMLAPYIPMDDDFQL-$CONH_2$), 50mM Tris pH 8.0, 5mM KCl, 1.5mM $MgCl_2$, 50 μM Iron (II), 1 mM DTT, 2mM Ascorbic Acid, 50-200 μM L-Leu NOG or equivalent volume of water in 10 μL water. Aliquots were kept on ice for 10 minutes before addition of 200 μM 2OG or equivalent volume of water. After 30-60 minutes incubation at 37°C, 1 μL sample and 1 μL S-DHB matrix were spotted on a MALDI plate and monitored with Bruker FlexControl in the reflectron positive mode and analyzed on Bruker FlexAnalysis software. Relative inhibition was monitored via assessing percent product decrease with L-Leu NOG samples compared to no L-Leu NOG samples.

MALDI-based assay for TET2: To determine the enzymatic activity of TET2 recombinant protein, 10 μM of protein was mixed with 5 μM 5-mC DNA substrate (5'-CAC5mCGGTG-3' and 5'-CAC5mCGGTG-3' (Identical palindromic sequences)), 50mM HEPES pH 7.5, 100mM NaCl, 1mM DTT, 1mM ATP, 100 μM Iron (II), 2mM Ascorbic Acid, and 50μM L-Leu NOG **6** or equivalent volume of water in 20μL aliquots. Samples were left to incubate on ice for 10 minutes and 1mM 2OG or equivalent volume of water was then added. After incubation at 37°C for 3 hours, samples were desalted with 3 μL of BT AG 50W-X8 RESIN (Bio-Rad 143-5441) at room temperature for 10 minutes. Samples were then spotted on a MALDI plate with 1uL sample and 1uL of 3-HPA matrix and monitored with Bruker FlexControl in the reflectron negative mode and analyzed on Bruker FlexAnalysis software. Relative inhibition was monitored via assessing percent product decrease with L-Leu NOG samples compared to no L-Leu NOG samples.

Dot blot-based assay for ALKBH2 on genomic DNA: To determine the effect of L-Leu NOG on ALKBH2 activity, we utilized a previously published method for ALKBH2 activity.[14] Briefly, 1μg of purified HEK293T cell gDNA was incubated with 10μM purified ALKBH2 recombinant wild type protein, 75 μM Iron (II), 1mM Ascorbic Acid, 150 μM KCl, 50mM HEPES buffer pH 7.5, and 50 μM L-Leu NOG or equivalent volume of water in 20 μL aliquots. Samples were allowed to incubate on ice for 10 minutes followed by addition of 1mM 2-OG or equivalent volume of water. Samples were allowed to shake at 37°C for 3 hours, followed by addition of 40μL of 2M NaOH-50mM EDTA solution for 3 minutes at room temperature. 140μL of 10mM Tris/1mM EDTA pH 8.0 (TE) buffer and heated at 95°C for 10 minutes. Aliquots were then transferred

directly to ice and 2M Ammonium Acetate was added and left on ice for 5 minutes. Meanwhile, PVDF Imoblinin P (Merck Millipore Ltd.) membrane was cut to size, activated with MeOH for 30 seconds and incubated in TE buffer for 5 minutes while shaking. The membrane was then placed on a 96-well Bio-Dot microfiltration apparatus (Bio-Rad), washed with 400μL of TE buffer and the gDNA solution was applied and vacuum filtered. 400μL of TE buffer was then used to the membrane post gDNA binding. The membrane was then dried at 37°C for 30 minutes followed by blocking in 5% milk concentrate (Bio-Rad) TBST solution for 1 hour at room temperature. Primary antibody 5-mC (Abcam ab214727) at 1-2000 dilution in 5% milk/TBST was applied and left to rotate overnight at 4°C. The following day, the blots were washed 3 times, 5 minutes each with TBST and secondary antibody of goat-anti Rabbit antibody (CST 7074) in 5% milk/TBST at 1-10000 dilution was added for 1 hour at room temperature while shaking. The blots were then washed with TBST 3 times, 5 minutes each. Thermo Scientific Pierce ECL blotting solution (PI32106) was then added, left to rotate at room temperature for 5 minutes and imaged using Bio-Rad imaging ChemiDoc. The blot was then washed with TBST 3 times, 5 minutes each and incubated with methylene blue solution (Sigma 03978) for loading determination.

11. Demethylase activity and inhibition of the KDM4 proteins on extracted histones

Histone extraction was carried out following protocol the protocol by D. Shechter et al.[20] The HEK293T cells are grown in 10% FBS and DMEM media to a 100% confluency and collected by trypsinization. The cells are washed twice with PBS buffer and incubated in hypotonic solution (10 mM Tris–Cl pH 8.0, 1 mM KCl, 1.5 mM $MgCl_2$, 1 mM DTT and 1X protease inhibitor) for 30 mins at 4°C. The intact nuclei were pelleted by centrifuging at 4°C, 10,000 g for 10 mins. The supernatant was discarded, and the nuclei was resuspended in 400 μL of 0.4 N H_2SO_4, rocking overnight. The samples were the centrifuged at 16000 g, 4°C for 10 mins to remove nuclear debris. The supernatant was transferred to a fresh 1.5 mL centrifuge tube, 132 μL of TCA was added dropwise to precipitate the histones and incubated on ice for 30 mins to 1.5 hr. The histones were pelleted by centrifuging at 16,000 g, 4°C for 4 mins. The supernatant was removed, and the pelleted histones were washed thrice with ice-cold acetone, centrifuging at 16,000g, 4°C for 5 mins after every wash. Finally, the histone pellet was air dried for 20 mins and re-suspended in deionized H_2O. 1 μL of histone extracts was loaded on a 15% gel with the BSA standards to quantify the amount of H3 for the assay. 5 μM of indicated protein in buffer containing 100 μM

$(NH_4)_2Fe(SO_4)_2$, 2 mM L-ascorbic acid and 50 mM Tris pH 8.0 were incubated with or without 150 μM of L-Leu NOG for 5 minutes on ice, after which substrate (300 μM 2OG and 2 μg of Histone extract) was added to the tube. The assay was incubated at 37°C for 1.5 hours, the samples were analyzed via western blotting using H3K9me3 specific antibody.

12. Examining cell permeability and toxicity of 6A and 6B

In a T-25 cell culture flask, HEK293T cells were treated with either 200 μM L-Leu octyl-ester NOG **6A**, L-Leu TFMB-ester NOG **6B**, or corresponding volume of DMSO. Following 24 hours of incubation at 37°C, media was removed and 3mL of 80:20 methanol: water was added while the flasks with cells were on dry ice. The flasks were then incubated in -80°C for 10 minutes to allow cell lysis. With the flasks on dry ice, the cells were scraped from the tissue culture flask, transferred to a 15mL centrifuge tube, and centrifuged at 14,000xg for 20 minutes. The pellet was discarded, and the supernatant was dried using speed vac machine to remove the methanol. The dried product was dissolved in 50μM of cell-grade water. To remove insoluble extract, samples were centrifuged 13,000xg for 10 minutes and 1μL of the resulting supernatant was dissolved in 400μL cell-grade water and submitted for HRMS. HRMS detection was set for the molecular weight of de-esterified L-Leu NOG product **6** for each sample and the corresponding esterified substrates. Relative de-esterification was determined by subtracting abundance of each treatment sample by the DMSO control and indicated molecular weights followed by dividing the abundance of L-Leu NOG molecular weight by the total abundance of esterified and de-esterified L-Leu **6** for each sample. Calculations were performed over 3 technical replicates with n=2 biological replicates each for a total of n=6.

To assess the toxicity of **6A/B** on HEK 293-T cells (ATCC CRL-1573) were counted, and 5,000 cells were plated into a 96 well cell culture plate (Corning) for n=5 of each treatment. The next day, the cells were washed with fresh media and **6A/B** from 100 to 800 μM or equivalent volume of DMSO. Cells were incubated at 37°C with 5% CO_2 supplementation for 24 hours and MTT assay processing ensued with CyQUANT™ MTT Cell Proliferation Assay Kit (V13154) according to manufacturer protocol. Absorbance at 570nm was quantified via TECAN Infinite M1000Pro plate reader.

13. Expression of full-length KDM4A-E and their mutants in HEK293T cells

KDM4A mammalian expression plasmid in CMV vector was purchased from Addgene (plasmid #24180). KDM4B mammalian expression plasmid in CMV vector was purchased from Addgene (plasmid #24181). KDM4C mammalian expression plasmid in CMV vector was purchased from Addgene (plasmid #24214). KDM4D mammalian expression plasmid in pReciever vector was purchased from Genecopoia (EX-Z0917-M06.) KDME mammalian expression plasmid in pRP-EXP was designed from Vector Builder. The KDM4A-F185A, KDM4B-F186A, KDM4C -F187A, KDM4D-F189A, and KDM4E F186A mutants were generated using Quikchange Lightening Mutagenesis Kit (Agilent) according to manufacturer's protocol and confirmed by Sanger sequencing.

HEK293T/17 cells (ATCC) were grown in Delbucco's Modified Eagle Medium (DMEM) (Corning) with 10% FBS at 37°C (Corning), 5% CO_2, and 95% relative humidity in a 6-well cell culture plate. When cells reached 90% confluency, 3 μg of plasmid and 6 μg lipofectamine 2000 (Invitrogen: 11668019) were incubated with 100 μL OptiMem. After 5 minutes the plasmid and lipofectamine were combined and incubated at room temperature for 20 minutes and added to the cell culture disk. Cells were returned to the incubator to grow for 4-6 hours. This was followed by treatment of compound treatment (n-octyl IOX1[21,22] or a specific NOG ester) at indicated concentrations and further culture for specified time. The media was removed, and cells were washed with ice cold PBS (Corning), then trypsinized with TrpLE Express (Gibco). The trypsinization reaction was quenched with DMEM 10% FBS and cells were pelleted then washed once with ice cold PBS. The nuclear extracts were generated by first resuspending the cell pellets in 700 μL nuclear isolation buffer (15 mM Tris pH 7.5, 60 mM KCl, 15 mM NaCl, 5 mM $MgCl_2$, 1 mM $CaCl_2$, 1mm DTT, 2 mM Na_3Van, 250 mM sucrose, 1X Pierce protease inhibitor (Thermo Fisher #PIA32955), 1 mM PMSF, 0.3% NP40 diluted to volume with MilliQ H_2O) and incubated on ice for 5 minutes.[23] The samples were then pelleted by centrifugation at 2000 rcf for 5 minutes. The supernatant was removed, and the pelleted nuclei were resuspended in 200 μL Pierce IP lysis buffer (Thermo Fisher #87787) containing 1X Pierce protease inhibitor and left on ice for 5 minutes. The nuclei were then sonicated at 100% amp for 5 minutes (pulsed 1 minute "on", 20 seconds "rest") using a Qsonica-Q700, cuphorn sonicator. The samples were pelleted once again at 2000 rcf for 5 minutes and the supernatant containing the nuclear extracts were collected. $MgCl_2$ (1M) solution was added to each sample give a final concentration of 6mM. The nuclear extracts

were quantified with Bradford assay (Bio-Rad laboratories) using BSA as a standard and used for Western blot analysis.

14. Expression and activity of full-length TET1 and 3 in HEK293T cells

To determine the effect of **6A** on TET1 and TET3 activity, HEK293T cells were plated in 6 well plates and transfected with full-length TET1/3 constructs. Briefly, 2ug of TET1/3 or Empty Vector control per well was added to 200µL of JetOptimus buffer and vortexed followed by addition of 3µL JetOptimus reagent. The solution was vortexed and left to sit at room temperature for 10 minutes. The solution was then added to 1.8mL of DMEM and added to the cells in culture plate. The cells were left for 4 hours at 37°C incubator at 5% CO_2. The media was aspirated and replaced with 2mL of DMEM and 100µM of **6A** was added or equivalent volume of DMSO and left in the incubator for 24 hours. Cells were then collected and subject to genomic DNA isolation using Fisher GeneJET gemoic DNA Purification Kit (Fisher K0721) following manufactures protocol. 1000ng of gDNA, as measured via nanodrop, was then subject to Dot Blot experiment. Briefly, 20µL of 1000ng gDNA was added to 40µL of 2M NaOH-50mM EDTA solution for 3 minutes at room temperature. 140µL of 10mM Tris/1mM EDTA pH 8.0 (TE) buffer and heated at 95°C for 10 minutes. Aliquots were then transferred directly to ice and 2M Ammonium Acetate was added and left on ice for 5 minutes. Meanwhile, PVDF Imoblinin P (Merck Millipore Ltd.) membrane was cut to size, activated with MeOH for 30 seconds and incubated in TE buffer for 5 minutes while shaking. The membrane was then placed on a 96-well Bio-Dot microfiltration apparatus (Bio-Rad), washed with 400µL of TE buffer and the gDNA solution was applied and vacuum filtered. 400µL of TE buffer was then used to wash the membrane post gDNA binding. The membrane was then dried at 37°C for 30 minutes followed by blocking in 5% milk concentrate (Bio-Rad) TBST solution for 1 hour at room temperature. Primary antibody 5-hmC (Abcam ab214728) 1-10000 dilution in 5% milk/TBST was applied and left to rotate overnight at 4°C. The following day, the blots were washed 3 times, 5 minutes each with TBST and secondary antibody of goat-anti Rabbit antibody (CST 7074) in 5% milk/TBST at 1-10000 dilution was added for 1 hour at room temperature while shaking. The blots were then washed with TBST 3 times, 5 minutes each. Thermo Scientific Pierce ECL blotting solution (PI32106) was then added, left to rotate at room temperature for 5 minutes and imaged using Bio-Rad imaging ChemiDoc. Blots were then

washed with TBST 3 times, 5 minutes each and incubated with methylene blue solution (Sigma 03978) for loading determination.

15. Determining the effect of 6A/B on post-translational modifications in HEK293T cells

To determine the effects of 6B on 5hmC and 5mC levels, and correspondingly any proteins responsible for addition or removal of these motifs, HEK293-T cells (ATCC CRL-1573) were plated into 6-well tissue culture plates (Corning) in DMEM (Corning) supplemented with 10% FBS (Corning) and 1X Antibiotic/Antimycin (Penicillin/Streptomycin/Fungiozone) (Cytiva HyClone) in an incubator held at 37°C with 5% CO_2. At 75% confluency, cells were treated with 200μM 6B or equivalent volume of DMSO and left to incubate at 37°C. 24 hours later, the media was aspirated, washed with 1X PBS (Corning) and uplifted via trypsinization (Gibco TrypLE™ Express Enzyme). Cells were centrifuged at 3000xg for 3 minutes, washed with 1X PBS and centrifuged again at 3000xg for 3 minutes.

To study the effect on genomic DNA, genomic DNA was extracted with Fisher GeneJET gemoic DNA Purification Kit (Fisher K0721) following manufactures protocol. 1000ng of gDNA, as measured via nanodrop, was then subject to Dot Blot experiment. Briefly, 20μL of 1000ng gDNA was added to 40μL of 2M NaOH-50mM EDTA solution for 3 minutes at room temperature. 140μL of 10mM Tris/1mM EDTA pH 8.0 (TE) buffer was added and heated at 95°C for 10 minutes. Aliquots were then transferred directly to ice and 200μL of 2M Ammonium Acetate was added and left on ice for 5 minutes. Meanwhile, PVDF Imoblinin P (Merck Millipore Ltd.) membrane was cut to size, activated with MeOH for 30 seconds and incubated in TE buffer for 5 minutes while shaking. The membrane was then placed on a 96-well Bio-Dot microfiltration apparatus (Bio-Rad), washed with 400μL of TE buffer and the gDNA solution was applied and vacuum filtered. 400μL of TE buffer was then used to wash the membrane post gDNA binding. The membrane was then dried at 37°C for 30 minutes followed by blocking in 5% milk concentrate (Bio-Rad) TBST solution for 1 hour at room temperature. Primary antibody 5-mC (Abcam ab214727) at 1-2000 dilution or 5-hmC (Abcam ab214728) 1-10000 dilution in 5% milk/TBST was applied and left to rotate overnight at 4°C. The following day, the blots were washed 3 times, 5 minutes each with TBST and secondary antibody of goat-anti Rabbit antibody (CST 7074) in 5% milk/TBST at 1-10000 dilution was added for 1 hour at room temperature while shaking. The

blots were then washed with TBST 3 times, 5 minutes each. Thermo Scientific Pierce ECL blotting solution (PI32106) was then added, left to rotate at room temperature for 5 minutes and imaged using Bio-Rad imaging ChemiDoc. Blots were then washed with TBST 3 times, 5 minutes each and incubated with methylene blue solution (Sigma 03978) for loading determination.

To study the effect of histone methyl motifs (H3K4me3, H3K9me3, H3K27me3, H3K36me3, H3K79me3) and hydroxyproline motif, cell pellets were lysed with RIPA buffer with EDTA (Fisher J61529) supplemented with 1X protease inhibitor tablet (Roche). Cell pellets were pulse sonicated at 100% amp for 5 minutes (pulsed 1 minute "on", 20 seconds "rest") using a Qsonica-Q700, cuphorn sonicator and pelleted at 15,000 rpm for 10 minutes at 4°C. The protein concentration was determined using Bradford assay kit (BioRad Laboratories) with BSA as a standard. 20ug of lysates were then subject to western blot procedure as mentioned below and probed with indicated primary antibody.

To study the effect of m6A modification via 6A/B, cell pellets were subject to total RNA isolation using PuroSPIN™ Total RNA Purification Kit (NK051-250) following manufacturer's protocol. 1000ug of RNA was then subject to dot blot analysis. Briefly, 1000ng of RNA in 20µL aliquots were added to 8.25µL of 4X RNA denaturation solution (16.4 M Formamide, 2.8 M Formaldehyde, 26.6 mM MPS Buffer (6.7 mM Sodium Acetate, 1.3mM EDTA, and 1.3mM EGTA)) and left to incubate at 95°C for 3 minutes. Samples were directly moved to ice and quenched with 33.2µL of 20X SSC buffer (3.0M NaCl, 0.3 M Sodium Citrate, pH 7.0) for 5 minutes and diluted to a final volume of 100µL with water. Meanwhile, PVDF Imoblinin P (Merck Millipore Ltd.) membrane was cut to size, activated with MeOH for 30 seconds and incubated in 10X SSC buffer for 5 minutes while shaking. The membrane was then placed on a 96-well Bio-Dot microfiltration apparatus (Bio-Rad), washed with 100µL of 10X SSC buffer and the RNA solution was applied, and vacuum filtered. Sample was then rinsed with 100µL of 10X SSC buffer. The membrane was dried at 37°C followed by crosslinking for 5 minutes with UV light at 254nm. Membrane was then subject to the protocol mentioned in the section General Protocol for Western Blot section.

16. Incorporation of Azidohomoalanine in HEK293T cells

HEK293T cells were passaged into a 6 well plate. The cells were grown overnight then transfected with 3 ug of indicated plasmid, KDM4A WT/F185A or KDM4D WT/F189A expression vector,

and 6 ug of Thermo Turbofect (FERR0531). KDM4A siRNA was transfected into cells using 10nM siRNA (Thermo 4392420) with 1:2 ug amount of Thermo Turbofect (FERR0531). Cells were washed 4 hours later and fresh DMEM was applied. The next day, the cells were treated with inhibitor (L-leucine NOG) and grown for 24 hours. The cells were subjected to a methionine/cysteine washout with Gibco™ DMEM, High Glucose, No Glutamine, No Methionine, No Cystine (21-013-024), supplemented with 4.5 g/L Glutamine and Sodium Pyruvate and 10% FBS, then treated with 2 mM L-azidohomoalanine (Sigma-900892-100MG) for 2 hours.[24] The cells were collected and snap frozen in liquid nitrogen for future cell lysis. The cells were thawed on ice then lysed with EDTA-free RIPA buffer and sonicated for 5 minutes, 1 minute on and 30 seconds off at 100 mA. The lysate was quantified with Bradford dye and 40 ug of lysate was incubated with 1 mM Tris[(1-benzyl-1H-1,2,3-triazol-4-yl)methyl]amine (TBTA Sigma- 678937-50MG), 0.04 mM TAMRA Alkyne, 1 mM $CuSO_4$ (MP Biomedicals 191415), and 2 mM Ascorbic Acid (Alfa Aesar 36237) to a total volume of 20 µL at 37°C for 15 minutes with gentle shaking. 8 ug of reaction was loaded onto 5% SDS-PAGE. Once the TAMRA band ran off the gel, it was imaged using the TAMRA program on BioRad ChemiDoc. The gel was then stained with Coomassie blue as a loading control.

For analysis of AHA incorporation measured by fixed cell imaging, HEK293-T cells (ATCC CRL-1573) were grown in a 24 well plate (Corning) on poly-D-lysine (Gibco A3890401) treated glass coverslips (ThermoScientific 12-545-81P) overnight and transfected the next day with 500 ng KDM4A WT/F185A or Empty Vector (EV) equivalent using 1:2 ug of Thermo Turbofect (FERR0531). KDM4A siRNA was transfected into cells using 10nM siRNA (Thermo 4392420) with 1:2 ug amount of Thermo Turbofect (FERR0531). Cells were washed 4 hours post transfection and fresh DMEM was added. 24 hours later, cells were treated with IOX-1, 6A, or DMSO equivalent volume. 24 hours later, cells were washed 3 times with 1X PBS, then fixed with 4% Paraformaldehyde in PBS (Thermo Scientific J19943-K2) for 10 minutes. Cells were then washed with 1X PBS three times and permeabilized with .5% Triton X-100 in PBS for 10 minutes. Cells were then washed with 1X PBS three times. Cells were then incubated with 1 mM Tris[(1-benzyl-1H-1,2,3-triazol-4-yl)methyl]amine (TBTA Sigma- 678937-50MG), 0.04 mM TAMRA Alkyne, 1 mM $CuSO_4$ (MP Biomedicals 191415), and 2 mM Ascorbic Acid (Alfa Aesar 36237) to a total volume of 200 µL at 37°C for 15 minutes in a humidified chamber. Cells were then washed three times with 1X PBS. The nuclei were stained with Hoechst stain 0.01 mg/mL in H2O

(Sigma B2883-25MG) for 30 seconds, washed with PBS and finally adhered to a slide with gelvatol. The cells were imaged at University of Pittsburgh Center for Biological Imaging on an Olympus Fluoview 1000 II, grant number 1S10OD019973-01 using excitation and emission wavelengths corresponding to that of Rhodamine. Images were analyzed on ImageJ open-source software and NIS elements software.

17. Analysis of rRNA expression in HEK293T cells using fluorescence in-situ hybridization

HEK293-T cells (ATCC CRL-1573) were grown in a 24 well plate (Corning) on poly-D-lysine (Gibco A3890401) treated glass coverslips (ThermoScientific 12-545-81P) overnight and transfected the next day with 500 ng KDM4A WT/F185A or Empty Vector (EV) equivalent using 1:2 ug of Thermo Turbofect (FERR0531). KDM4A siRNA was transfected into cells using 10nM siRNA (Thermo 4392420) with 1:2 ug amount of Thermo Turbofect (FERR0531). After four hours, cells were washed and treated with 5-10 μM of cell permeable IOX-1 or 100-200 μM 6A/B in DMEM with FBS and antibiotic supplements, as stated above. Cells were left to incubate at 37°C at 5% CO_2 for 24 hours. Cells were washed 3 times with 1X PBS, then fixed with 4% Paraformaldehyde in PBS (Thermo Scientific J19943-K2) for 10 minutes. Cells were then washed with 1X PBS three times and permeablized with .5% Triton X-100 in PBS for 10 minutes. Cells were then washed with 1X PBS three times followed by washing with 2X SSC (0.3 M NaCl, 0.03 M sodium citrate, pH 7.0) and hybridized with FAM-labeled probe of antisense pre-rRNA in 200 μL volume of 50% formamide, 10% dextran sulfate and to volume of 2X SSC at 37°C in a humidified box at for 16 hours. The coverslips were then washed 3 times with 2X SSC and 3 times with 1X SCC. The nuclei were stained with Hoechst stain 0.01 mg/mL in H_2O (Sigma B2883-25MG) for 30 seconds, washed with PBS and finally adhered to a slide with gelvatol. The cells were imaged at University of Pittsburgh Center for Biological Imaging on an Olympus Fluoview 1000 II, grant number 1S10OD019973-01. Images were analyzed on ImageJ open-source software and NIS elements software.

18. Analysis of rRNA promoter demethylation in HEK293T cells

HEK293T cells were plated on 150 cm plates to be at ~75% confluency the next day. The plates were transfected with 15 μg indicated plasmid and 30 μg lipofectamine 2000 (Invitrogen 11668019). 6 hours post-transfection indicated inhibitor was added and incubated for 30 hours. 30

hours after addition of inhibitor the cells were ~95% confluent, and they were subsequently crosslinked with formaldehyde, following the protocol for Cell Signaling Technology SimpleChIP kit (#9005). The following steps were also performed following the protocol for the SimpleChIP kit. Nuclei were then isolated, chromatin was digested, and analyzed for quality and concentration. 10 µg of chromatin, and reserving a 2% input sample prior to antibody binding, was incubated with 10 µg of H3K9me3 antibody (CST 13969S) overnight at 4°C with gentle rotation, the next day protein G magnetic beads were incubated with antibody-antigen complex for 2hours. The crosslinking was reversed, and protein was degraded using proteinase K. DNA was purified through the provided columns and 2 µL of purified eluted DNA was mixed with 2 µL of each forward and reverse primers, and 10 µL of PerfeCTa SybrGreen Supermix (QuantaBio 95054-500). Each sample was measured in triplicates. qPCR was performed on BioRad C1000 Touch Thermocycler CFX96 Real Time System, at 55°C for 3 minutes, then 55 cycles of 95°C for 10 seconds followed by 55°C for 30 seconds. Values were measured using percentage of the input method.

19. Fixed cell immunofluorescence staining

Cells were grown on poly-D-lysine (Gibco A3890401) treated glass coverslips (ThermoScientific 12-545-81P) overnight and transfected the next day with 300 ng KDM4A WT/F185A using 600 ng of lipofectamine 2000 (Invitrogen 11668019). The cells were incubated overnight and treated with indicated inhibitor at varying time points. When probing rRNA transcription, cells were incubated with 2 mM 5-Fluorouridine (Sigma F5130) for 15 minutes. Cells were washed three times with phosphate buffered saline (PBS) at pH 7.6 (Corning 21040CV), fixed with 2% paraformaldehyde in PBS (ThermoFisher AAJ19943K2) for 15 minutes at room temperature, washed with PBS three times, and permeabilized with 0.1% tritonX (Fisher BP151-100) in PBS for 15 minutes. Next, coverslips were blocked with 2% Bovine Serum Albumin (BSA Fisher BP1600-100) in PBS for 1 hour at room temperature, coverslips were incubated with primary antibody diluted in 0.5% BSA PBS (anti-H3K9me3: Cell Signaling Technology cat: 13969S host:rabbit 1:1000; anti-HA: Cell Signaling Technology host:mouse, cat: 2367S or host:rabbit, cat: 3724S, both 1:1000; or anti-BrdU BD Biosciences host: mouse, cat: BDB555627, 1:500) for 1 hour at room temperature, then incubated with secondary antibodies diluted in 0.5% BSA PBS (anti-mouse—488: Invitrogen host: goat, cat: A-11001 or anti-rabbit—cy3: Invitrogen host: goat,

cat: A10520, both dilutions 1:1000), between each step coverslips were washed five times with 0.5% BSA PBS. The nuclei were stained with Hoechst stain 0.01 mg/mL in H_2O (Sigma B2883-25MG) for 30 seconds, washed with PBS and finally adhered to a slide with gelvatol. The cells were imaged at University of Pittsburgh Center for Biological Imaging on an Olympus Fluoview 1000 II, grant number 1S10OD019973-01.

20. Quantification of fixed-cell imaging data

TIF images obtained from confocal microscopy experiments were quantified in ImageJ. Images were converted to 8-bit images and then thresholded to remove background signal. All images within the same experiment used the same threshold values. For concentration dependent imaging experiments, FISH, and AHA experiments, images were cropped to contain individual cells. For each cell image, the selection tool was used to select the area within individual cells or cell nuclei. The mean fluorescent signal was then measured. Each cell measurement was plotted in GraphPad Prism with the corresponding bars representing the mean and standard deviation of the population. For the time dependent imaging, cells containing the transfected KDM4A mutant were identified by their HA signal. These cells were used to create a selection which was then imposed onto the respective H3K9me3 or BrdU images. The mean signal was then measured for H3K9me3 or BrdU only within the cells expressing the KDM4A mutant. To compare changes in H3K9me3 or BrdU between trials, the quantification values were normalized to the mean signal at t=0. The normalized, mean signal and error for H3K9me3 or BrdU were then plotted in GraphPad Prism.

21. General protocol for Western blotting

Assay samples were mixed with appropriate volume of 4X Laemmli Dye (Bio-Rad), heated at 95°C for 5 minutes and loaded onto 4–12% Criterion™ XT Bis-Tris protein gels (Bio-Rad #3450123) or in-house prepared SDS-polyacrylamide gel and subjected to electrophoresis (Criterion Precast Tank, Bio-Rad 1656001) typically at 150V for 30 min - 1 hour in 1X MES buffer (prepared from Invitrogen™ Novex™ 20X Bolt™ MES SDS Running Buffer). After electrophoresis, gels were removed from cassette and transferred onto a 0.2 mm supported nitrocellulose membrane via semidry blotting apparatus (Bio-Rad Laboratories) at a constant voltage of 15 V for 30 minutes or via wet blotting tank at 80V for 1 hr. (or at 40V for 2.5 hr.) at 4°C. Occasionally, 0.45 μm nitrocellulose membrane (BIORAD cat #1620112) or 0.2 μm PVDF

(Immobilon-PSQ ISEQ00010) membrane preactivated with methanol for 30 seconds was also used. For semidry transfer, gels were incubated for 5 minutes with semidry transfer buffer (48mM Tris Base, 39 mM glycine, 0.0375% SDS, 20% methanol) followed by transfer via semidry blotting apparatus (Bio-Rad #1703940) at a constant voltage of 15 V for 30 minutes or at a constant amplitude of 5.5 mA per square centimeter for 30 minutes with a maximum voltage of 25 V. After transfer, membranes were blocked with 5% Bovine Serum Albumin (BSA) in TBST buffer (50 mM Tris HCl pH 7.6, 200 mM NaCl, 0.1% Tween 20) for 1 hour at room temperature with gentle shaking. The blocking buffer was removed, and membranes were washed three times with 20 mL of TBST buffer. Immunoblotting was performed with diluted primary antibodies (Table S3) at 4°C overnight with gentle shaking. The antibody solution was removed, and the membranes were washed with TBST 3 times for 5 minutes each, then incubated with 1:10000 dilute HRP-conjugated secondary antibody Goat anti-Rabbit IgG (cat #7040 Cell Signaling Technology) or Goat anti-Mouse IgG (cat #7076 Cell Signaling Technology) for 2 hours at room temperature. To remove the secondary antibodies, the membrane was washed with TBST 3 times, 5 minutes each. Subsequently, the membrane was incubated with Pierce™ ECL Western Blotting Substrate (ThermoScientific cat # PI32160) following manufacturer's protocol. The membrane was imaged on BioRad chemidoc and analyzed with BioRad Image Lab software.

22. Histone extraction, digestion and tandem mass spectrometry

HEK 293-T cells (ATCC CRL-1573) were plated into 6-well plates (Corning) in DMEM (Corning) supplemented with 10% FBS (Corning) and 1X Antibiotic/Antimycin (Penicillin/Streptomycin/Fungiozone) (Cytiva HyClone) in an incubator held at 37°C with 5% CO_2. At 75% confluency, cells were transfected with 2.5 µg KDM4A F185A or Empty Vector with 5µg Thermo Turbofect (FERR0531). After 4 hours, the media was washed and 200 µM of **6A** or equivalent volume of DMSO was added and left to incubate at 37°C for 24 hours. Cells were then washed with 1X PBS (Corning) followed by trypsinization (Gibco TrypLE™ Express Enzyme). Collected cells were centrifuged at 3000xg for 3 minutes, followed by washing with 1X PBS and centrifuged for an additional 3000xg for 3 minutes. Histone proteins were extracted from the pellet as described by[25] to ensure good-quality identification and quantification of single histone marks. Briefly, cell pellets were dissolved in 100 µL of cold 0.2 M H_2SO_4 and incubated for 2 hours rotating in a cold room (4°C). Aliquots were then centrifuged at 3400xg for 5 minutes

at 4°C and the supernatant was added to a fresh tube. 100% cold TCA was then added to a final volume of 33% volume/volume TCA/supernatant and left to incubate for 1 hour in a cold room (4°C). Samples were then centrifuged at 3400xg for 5 min at 4°C and the supernatant was discarded. The precipitate was then washed with cold acetone/0.1% HCl and centrifuged at 3400xg for 5 minutes at 4°C. The supernatant was discarded, and the precipitate was washed with cold acetone and centrifuged at 3400xg for 5 minutes at 4°C. The supernatant was discarded, and the pellet was dried using a vacuum centrifuge.

The pellet was dissolved in 50 mM ammonium bicarbonate, pH 8.0, and histones were subjected to derivatization using 5 µL of propionic anhydride and 14 µL of ammonium hydroxide (all Sigma Aldrich) to balance the pH at 8.0. The mixture was incubated for 15 min and the procedure was repeated. Histones were then digested with 1 µg of sequencing grade trypsin (Promega) diluted in 50mM ammonium bicarbonate (1:20, enzyme:sample) overnight at room temperature. Derivatization reaction was repeated to derivatize peptide N-termini. The samples were dried in a vacuum centrifuge.

Sample desalting: Prior to mass spectrometry analysis, samples were desalted using a 96-well plate filter (Orochem) packed with 1 mg of Oasis HLB C-18 resin (Waters). Briefly, the samples were resuspended in 100 µl of 0.1% TFA and loaded onto the HLB resin, which was previously equilibrated using 100 µl of the same buffer. After washing with 100 µl of 0.1% TFA, the samples were eluted with a buffer containing 70 µl of 60% acetonitrile and 0.1% TFA and then dried in a vacuum centrifuge.

LC-MS/MS Acquisition and Analysis: Samples were resuspended in 10 µl of 0.1% TFA and loaded onto a Dionex RSLC Ultimate 300 (Thermo Scientific), coupled online with an Orbitrap Fusion Lumos (Thermo Scientific). Chromatographic separation was performed with a two-column system, consisting of a C-18 trap cartridge (300 µm ID, 5 mm length) and a picofrit analytical column (75 µm ID, 25 cm length) packed in-house with reversed-phase Repro-Sil Pur C18-AQ 3 µm resin. Peptides were separated using a 30 min gradient from 1-30% buffer B (buffer A: 0.1% formic acid, buffer B: 80% acetonitrile + 0.1% formic acid) at a flow rate of 300 nl/min. The mass spectrometer was set to acquire spectra in a data-independent acquisition (DIA) mode.

Briefly, the full MS scan was set to 300-1100 m/z in the orbitrap with a resolution of 120,000 (at 200 m/z) and an AGC target of 5x10e5. MS/MS was performed in the orbitrap with sequential isolation windows of 50 m/z with an AGC target of 2x10e5 and an HCD collision energy of 30.
Histone peptides raw files were imported into EpiProfile 2.0 software [26]. From the extracted ion chromatogram, the area under the curve was obtained and used to estimate the abundance of each peptide. In order to achieve the relative abundance of post-translational modifications (PTMs), the sum of all different modified forms of a histone peptide was considered as 100% and the area of the particular peptide was divided by the total area for that histone peptide in all of its modified forms. The relative ratio of two isobaric forms was estimated by averaging the ratio for each fragment ion with different mass between the two species. The resulting peptide lists generated by EpiProfile were exported to Microsoft Excel and further processed for a detailed analysis.

23. Supplementary figures and tables

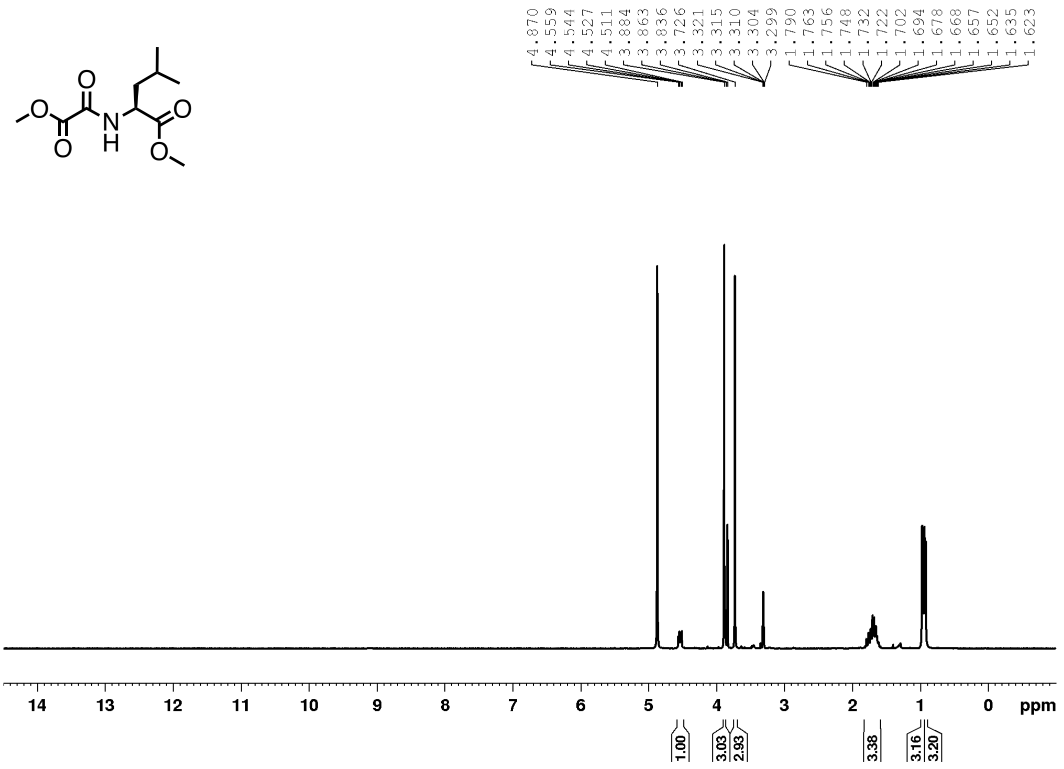

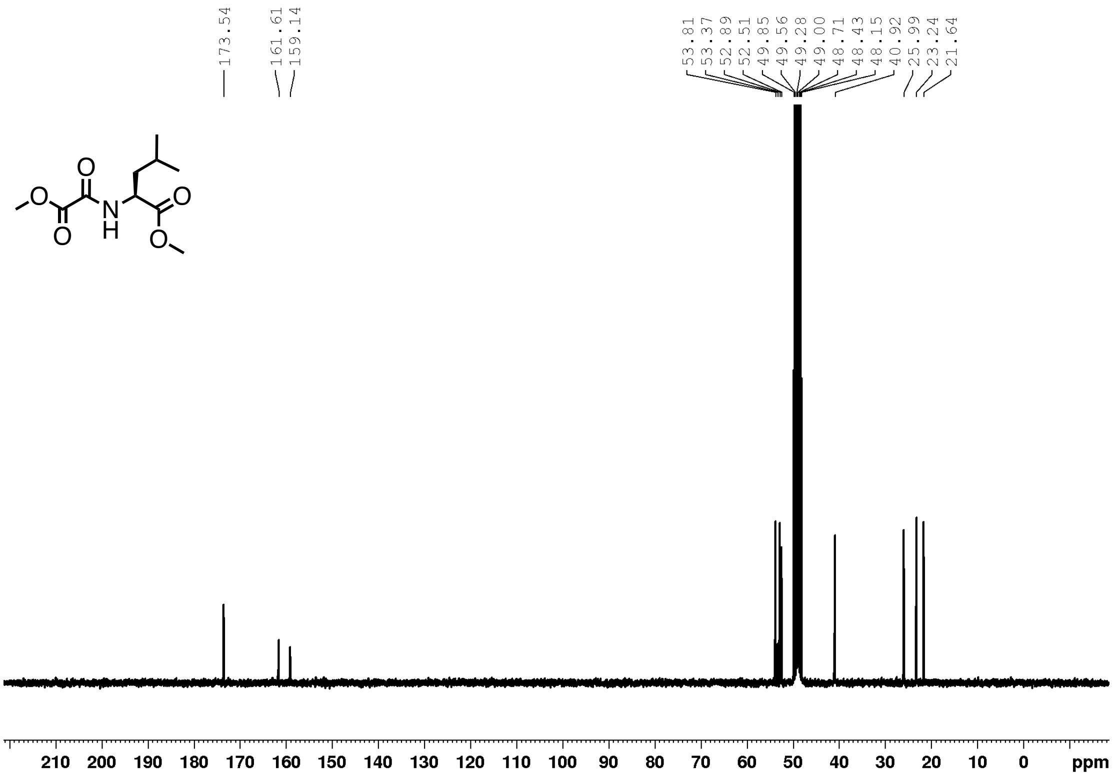

Supplementary Figure S1. ^{1}H and ^{13}C NMR spectra of the indicated compound in CD_3OD.

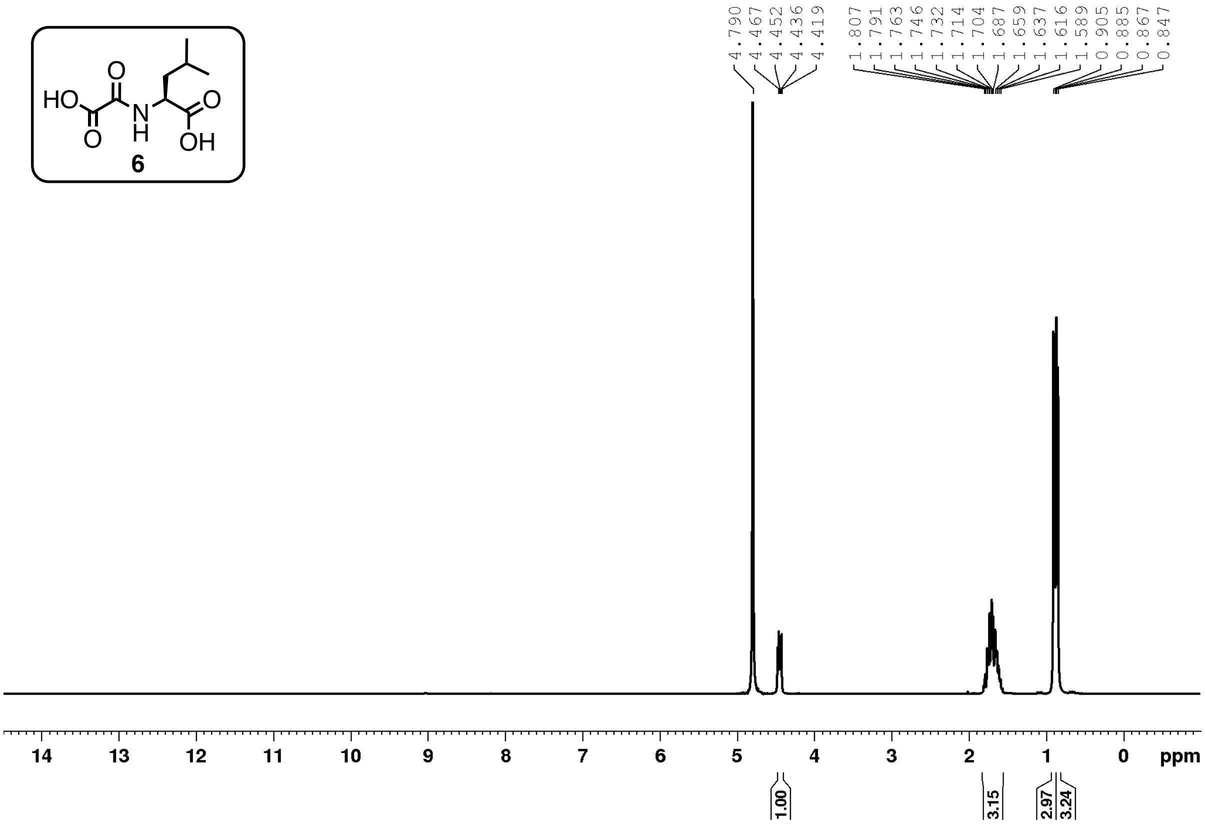

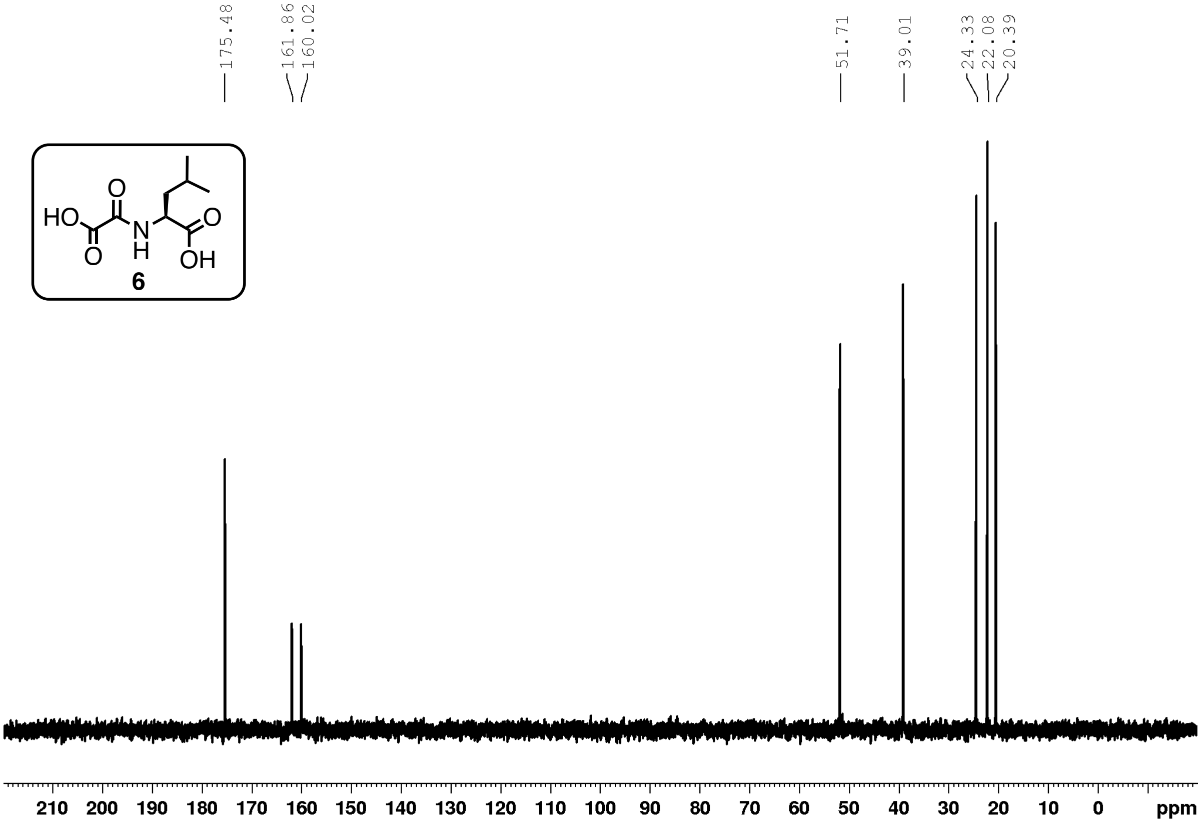

Supplementary Figure S2. ^{1}H and ^{13}C NMR spectra of **6** in D_2O.

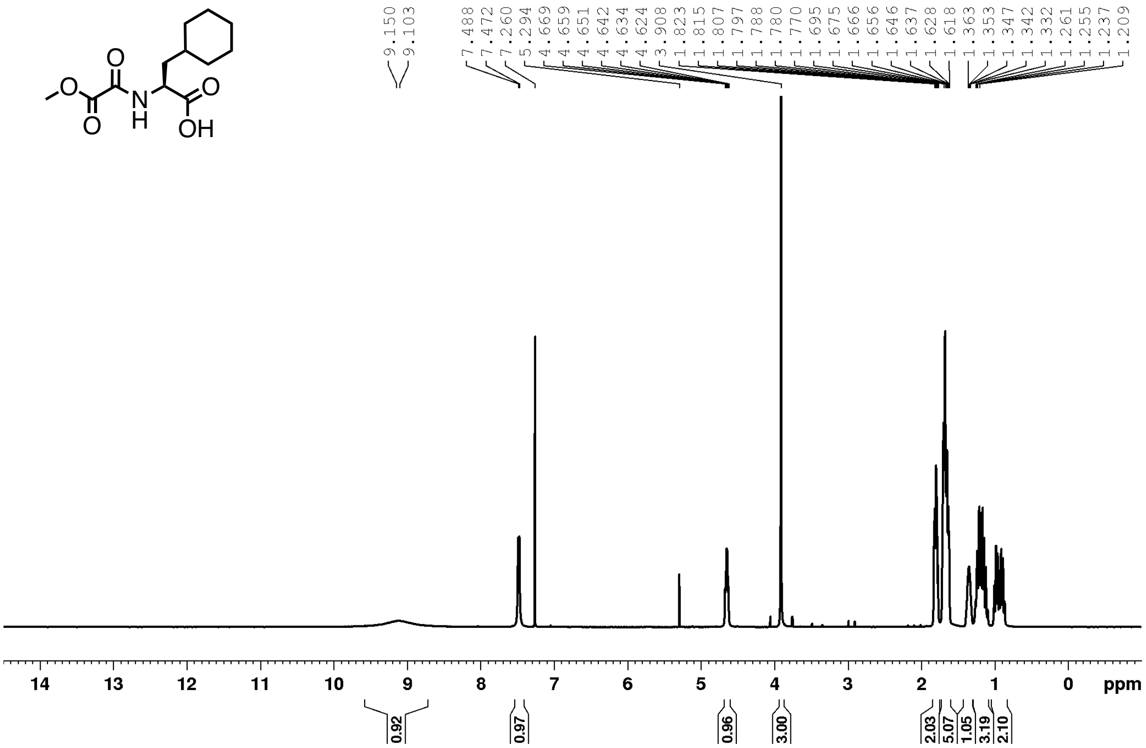

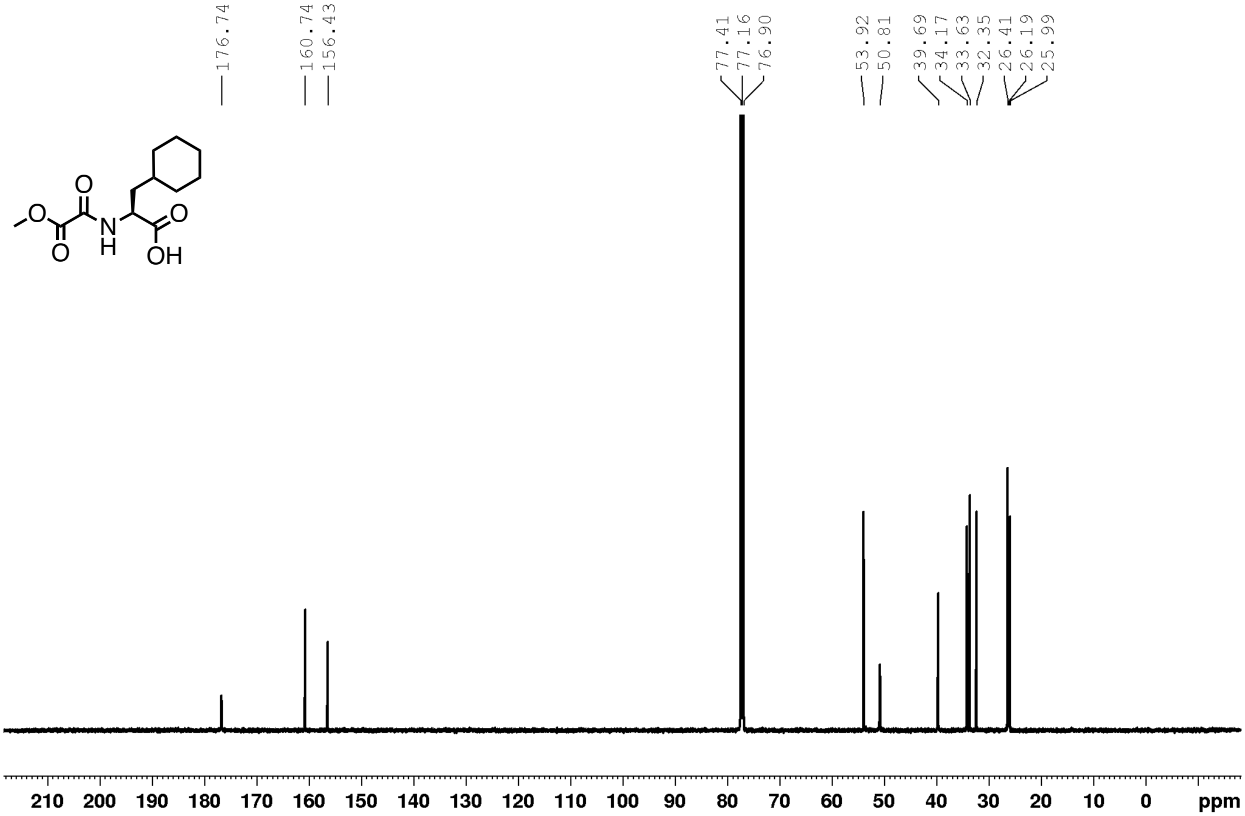

Supplementary Figure S3. ^{1}H and ^{13}C NMR spectra of the indicated compound in CD_3Cl.

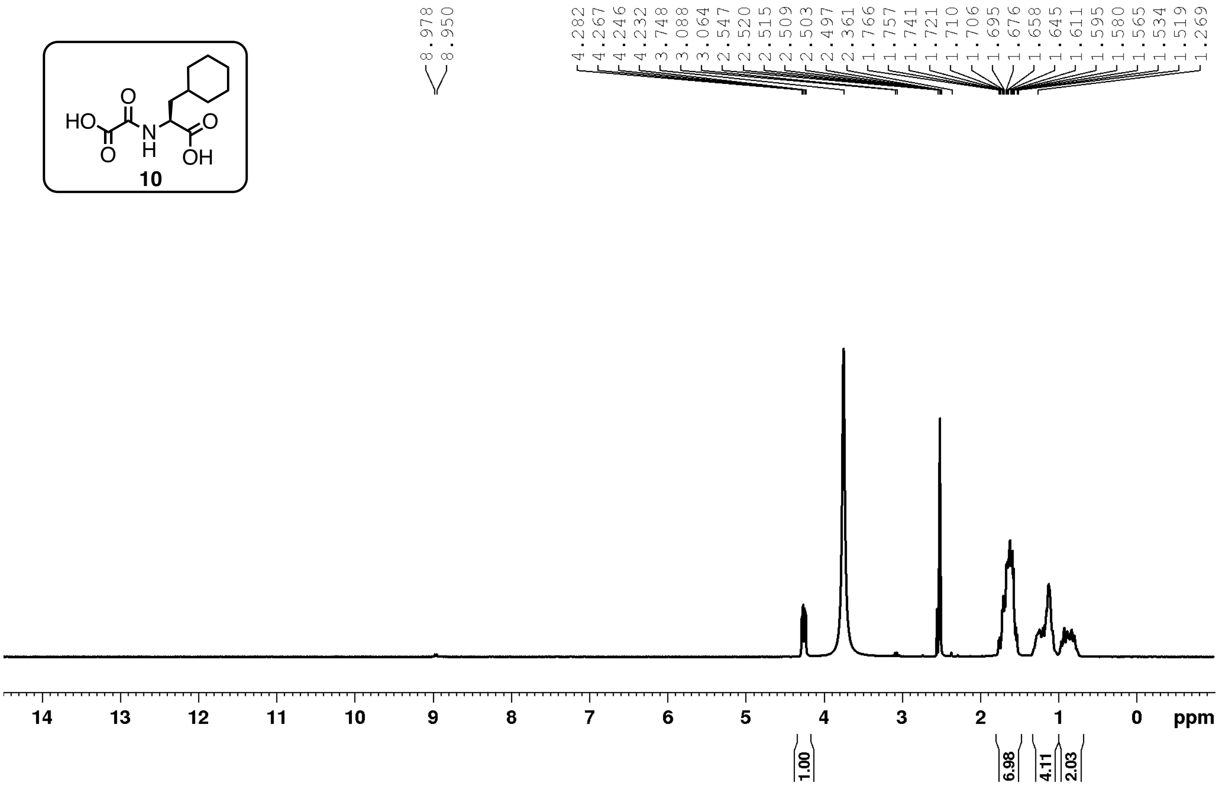

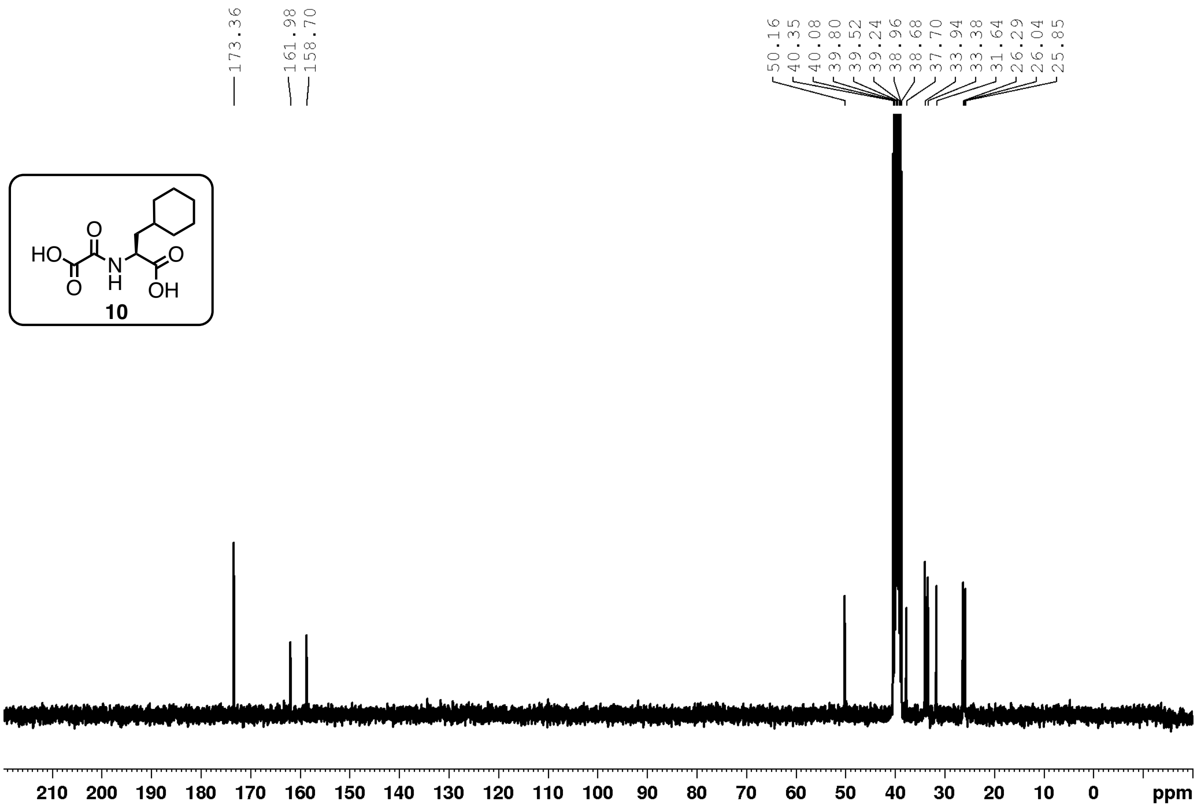

Supplementary Figure S4. ^{1}H and ^{13}C NMR spectra of **10** in DMSO-d6.

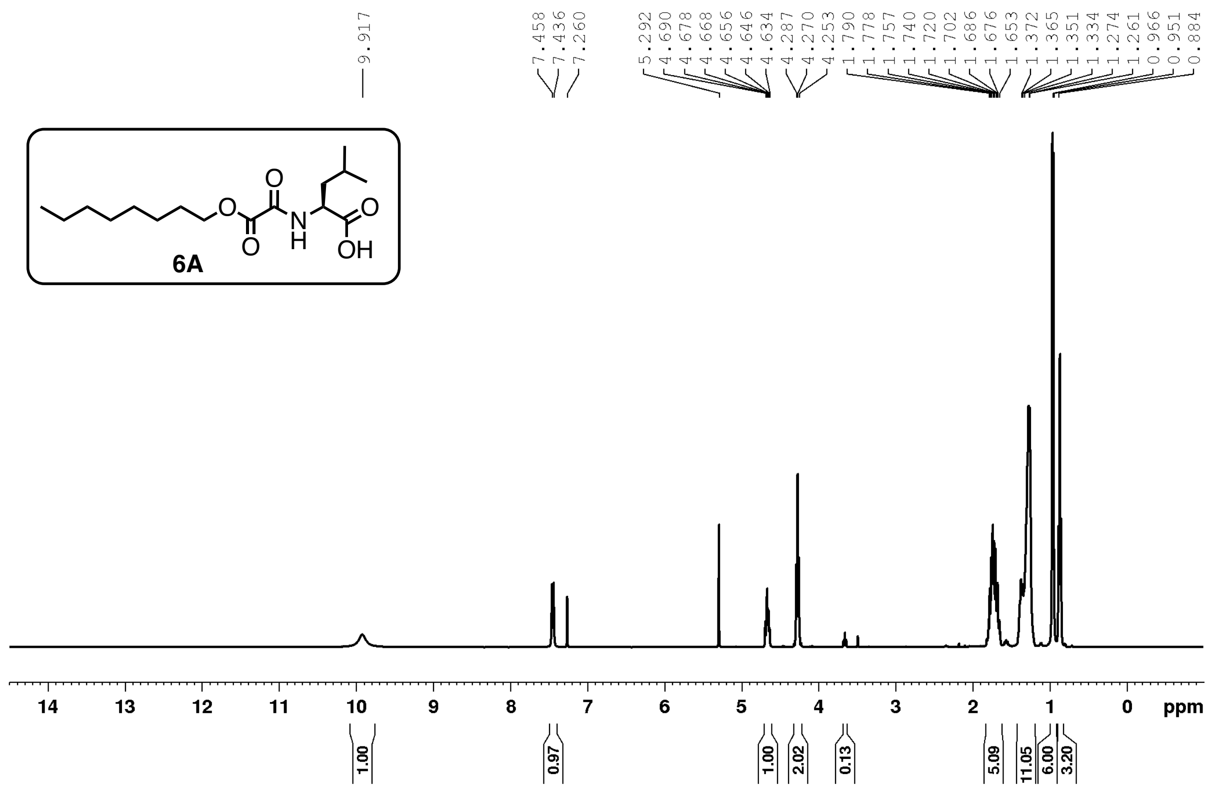

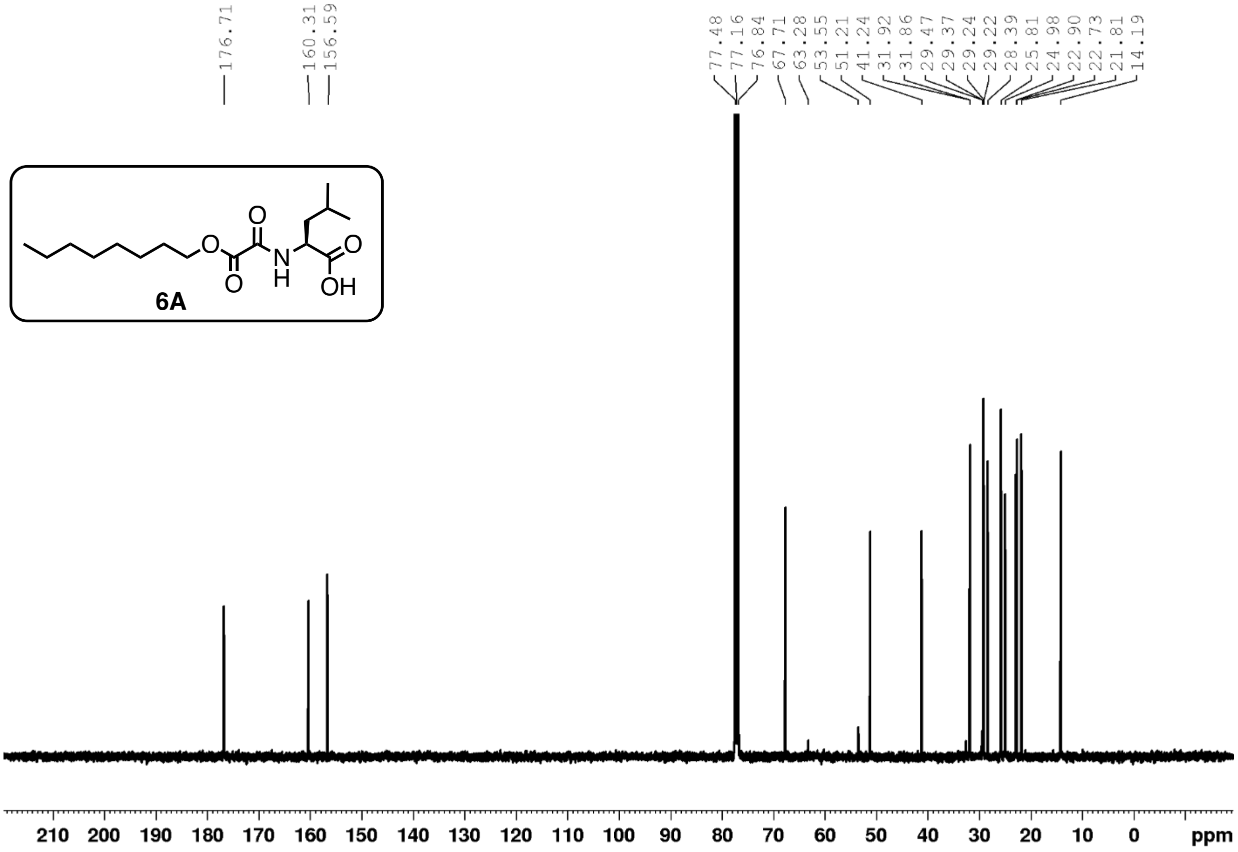

Supplementary Figure S5. ^{1}H and ^{13}C NMR spectra of **6A** in CD_3Cl.

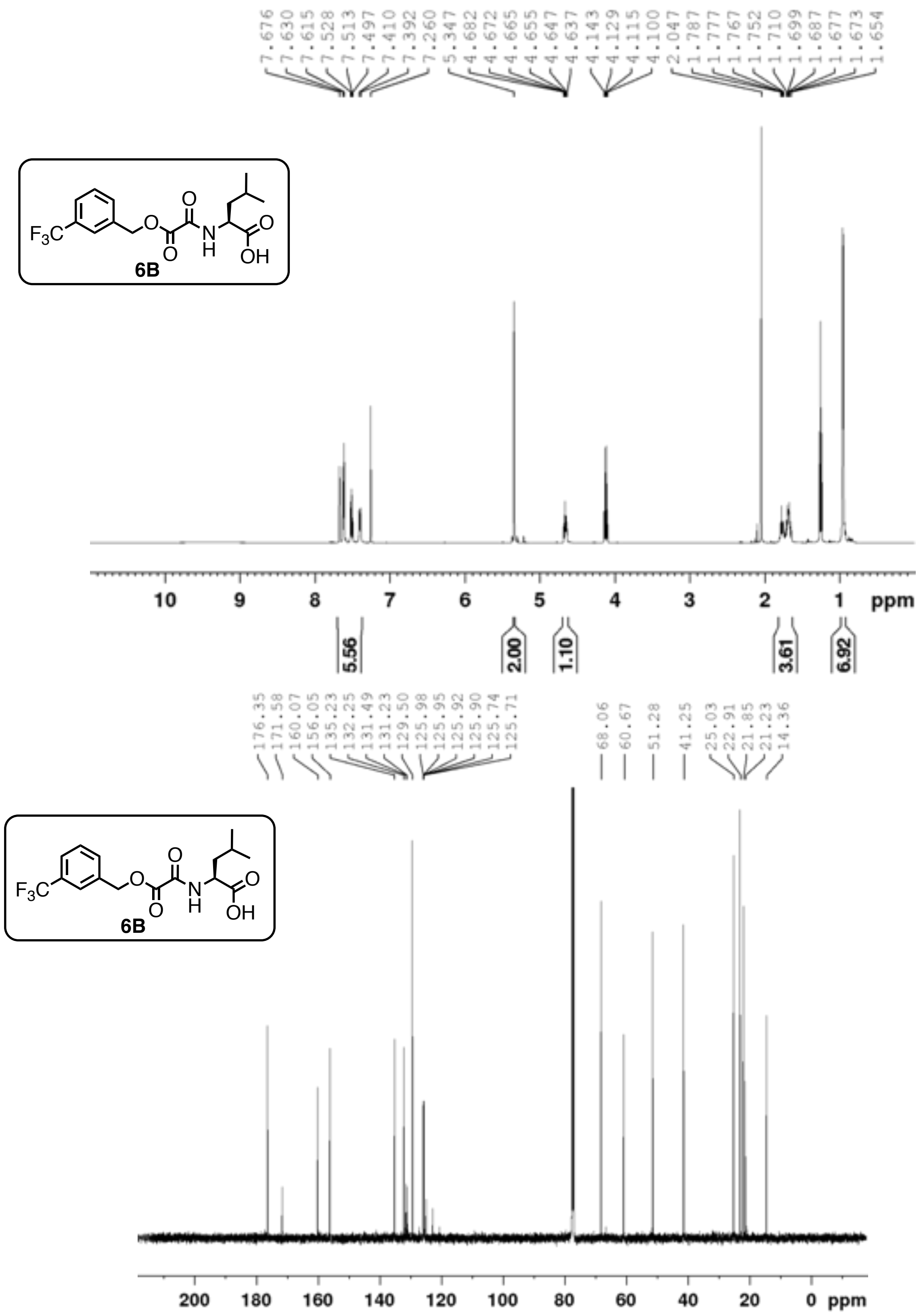

Supplementary Figure S6. ^{1}H and ^{13}C NMR spectra of **6B** in CD_3Cl.

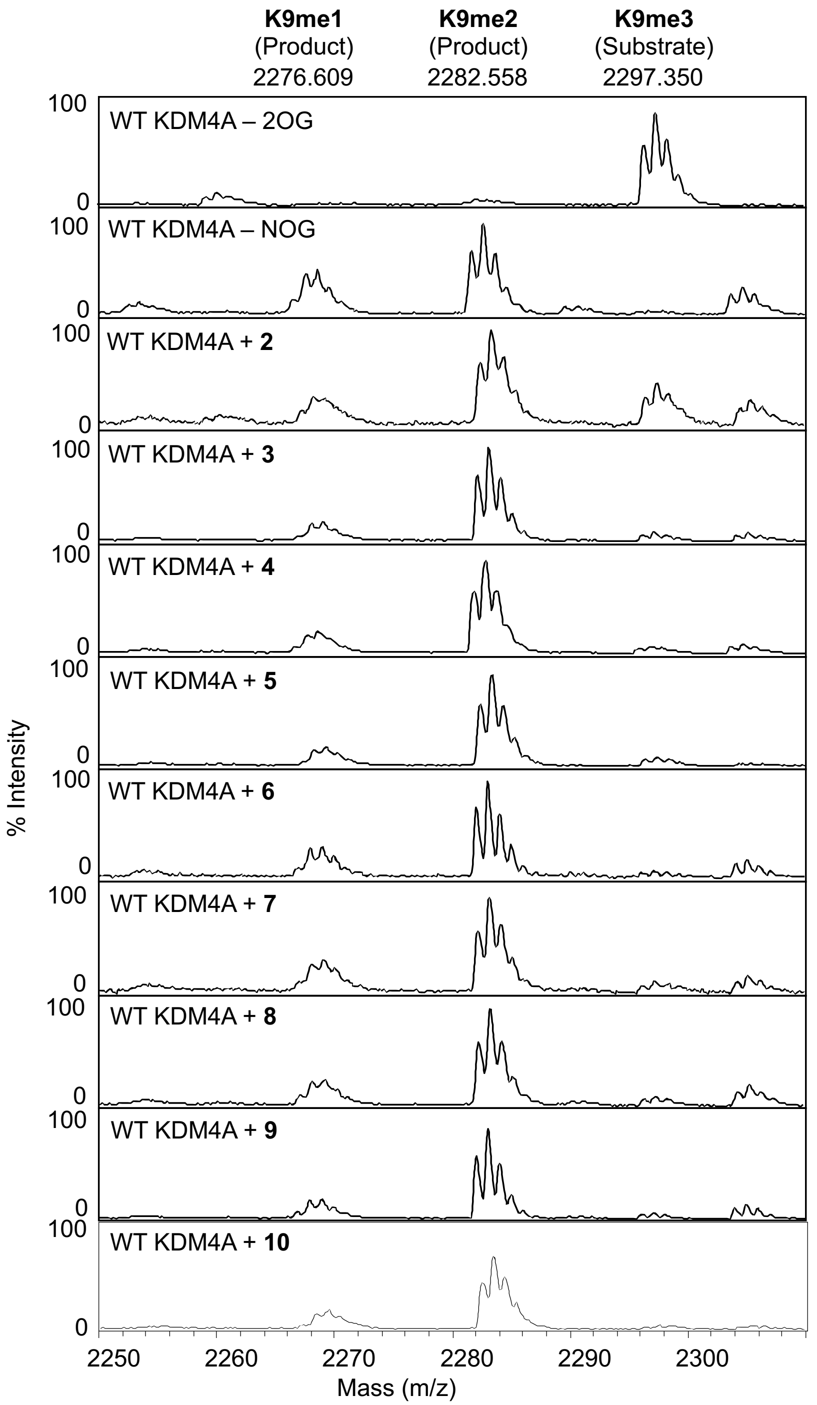

Supplementary Figure S7. MALDI-TOF spectra of demethylase activity of wild type KDM4A towards H3K9me3 peptide in the presence of NOG analogues. The topmost panel represents assay condition with no cofactor (2OG) added. The second panel represents assay condition with cofactor (2OG) added without any NOG inhibitor.

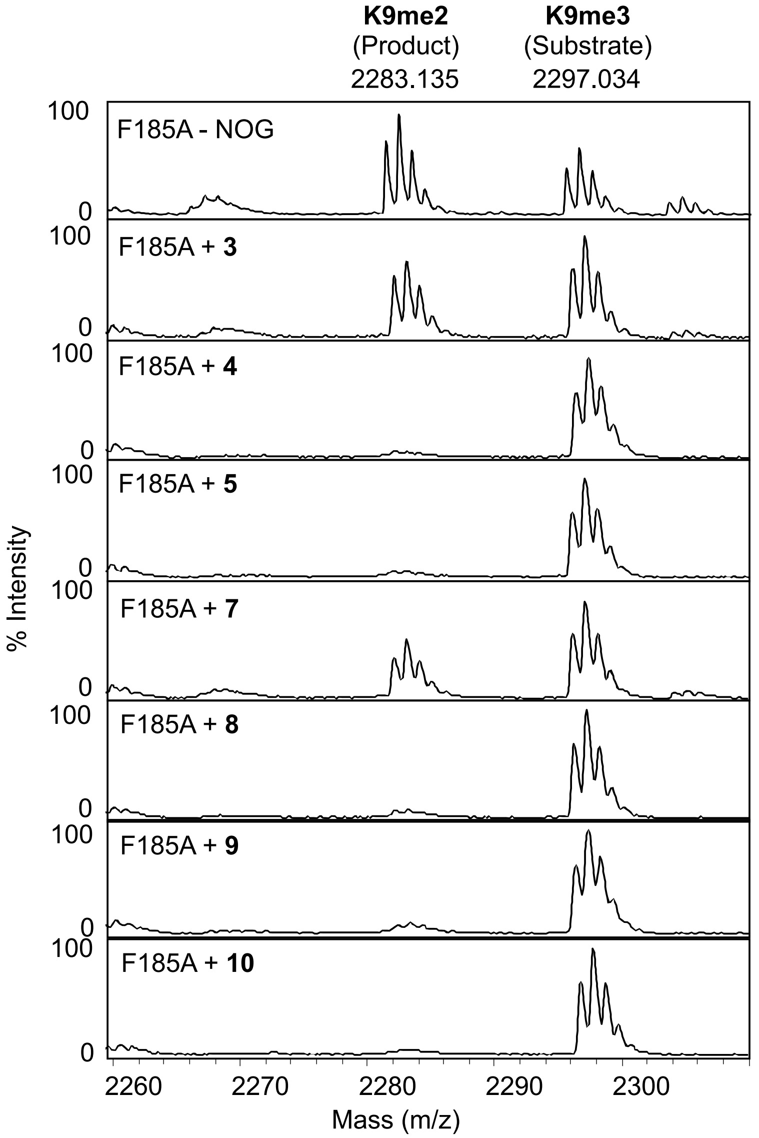

Supplementary Figure S8. MALDI-TOF spectra of demethylase activity of KDM4A-F185A mutant towards H3K9me3 peptide in the presence of NOG analogues. The topmost panel represents assay condition with cofactor (2OG) added without any NOG inhibitor. The MALDI spectra for NOG **2** and **6** are provided in Figure 2C in the manuscript.

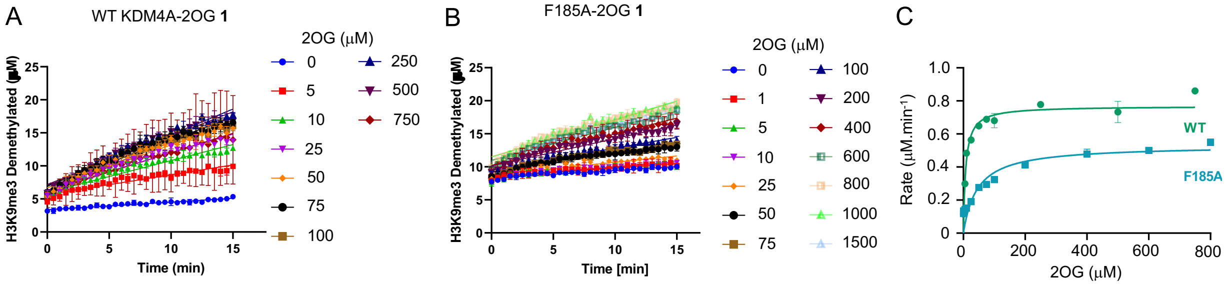

Supplementary Figure S9. Kinetic constants of the wild type and mutant KDM4A. (**A-B**) Time- and 2OG concentration-dependent increase of NADH fluorescence intensity as measure of the catalytic activity of wild type KDM4A-**1** (**A**), and F185A-**1** (**B**) pairs. **(C)** Michaelis-Menten curves for KDM4A-**1** and F185A-**1** pairs to determine the kinetic parameters (k_{cat}, K_M and k_{cat}/K_M).

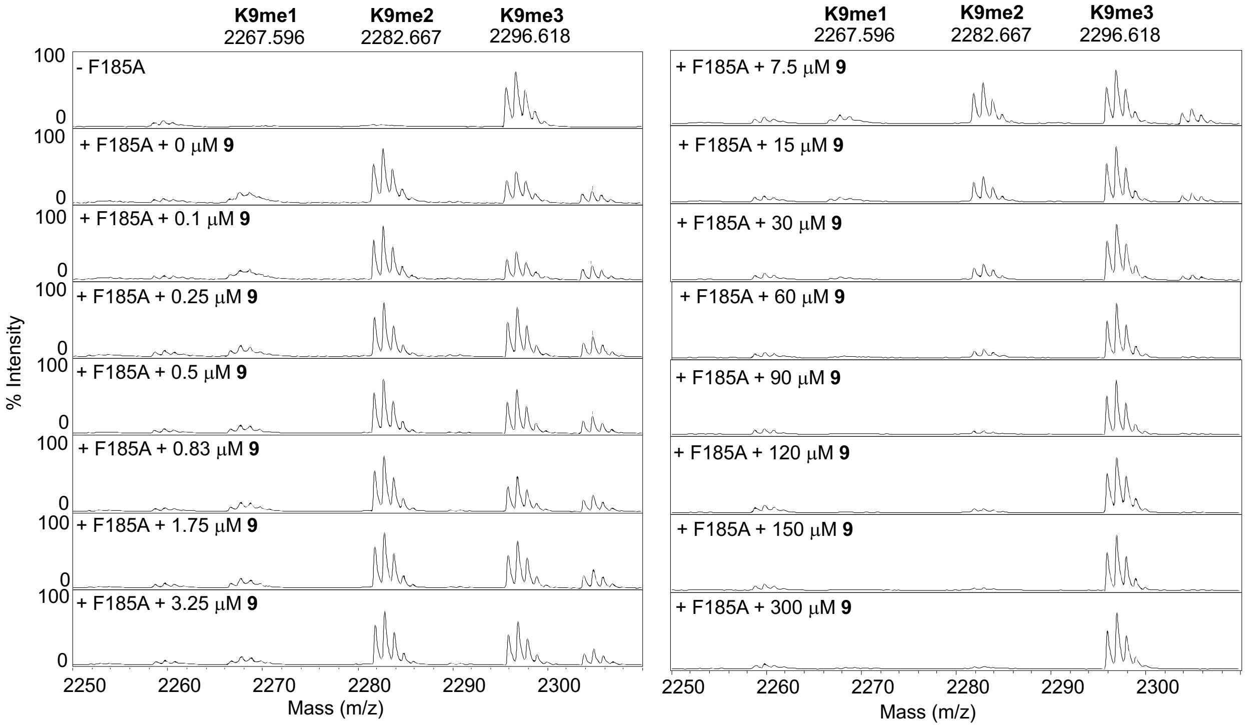

Supplementary Figure S10. Representative MALDI-TOF spectra for dose-dependent inhibition of KDM4A-F185A mutant by benzyl-NOG **9**.

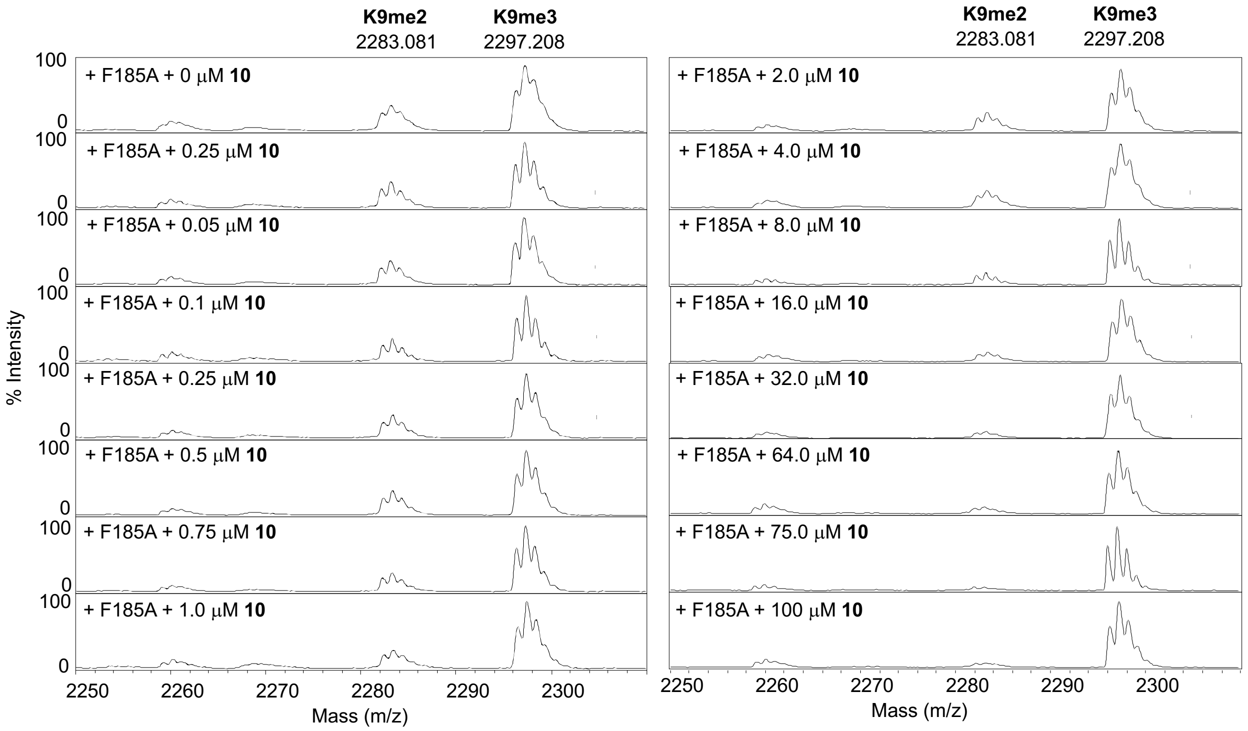

Supplementary Figure S11. Representative MALDI-TOF spectra for dose-dependent inhibition of KDM4A-F185A mutant by cyclohexyl-NOG **10**.

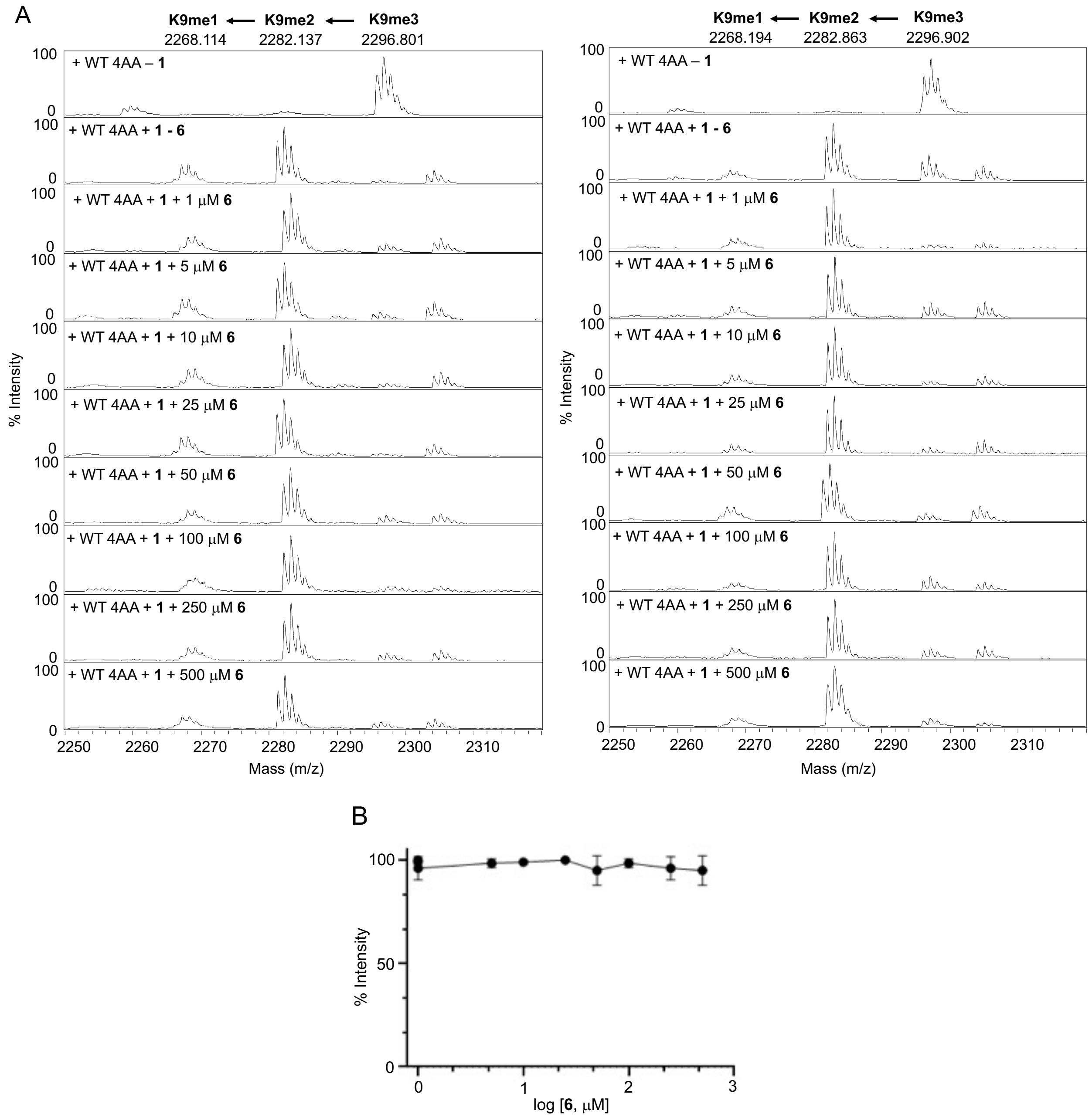

Supplementary Figure S12. (A) MALDI-TOF spectra for dose-dependent inhibition of wild type KDM4A by NOL **6** (n=1,2). (B) Quantitative representation of % activity obtained by MALDI-MS confirms **6** does not inhibit wild type KDM4A.

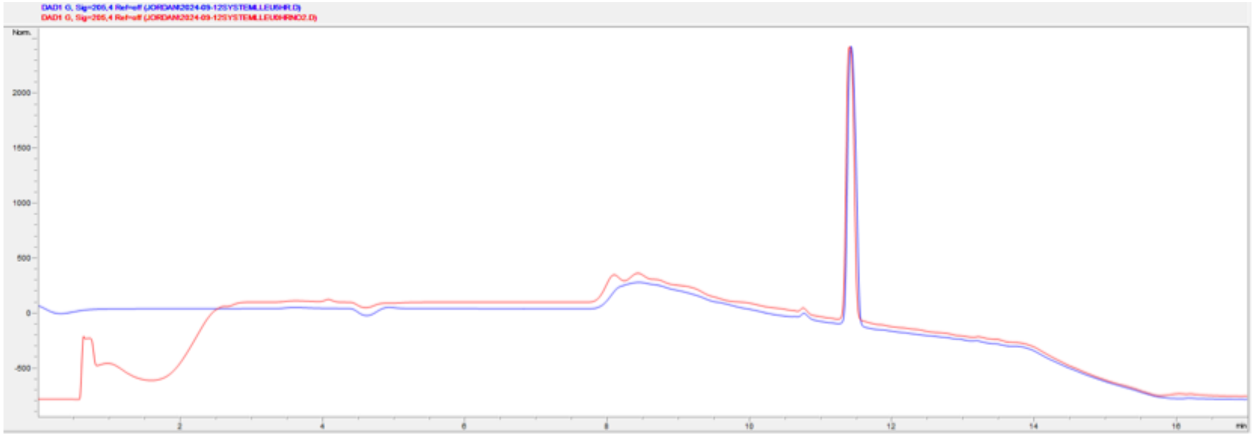

Supplementary Figure S13. HPLC traces of NOL **6** at t=0 hr. (red) and t=5 hr. (blue) incubation in assay buffer confirm the stability of the compound. The compound integrity was further confirmed by LC-MS.

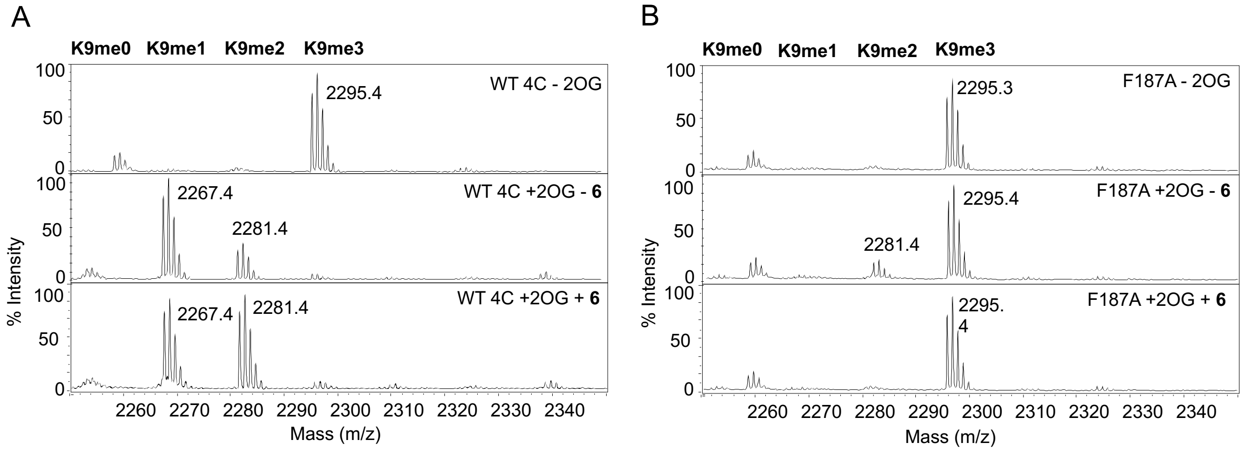

Supplementary Figure S14. MALDI-TOF spectra for demethylase activity of wild type KDM4C (A) and KDM4C-F187A mutant (B). In each case, topmost panel is assay without the cofactor 2OG (negative control), middle panel is assay with the cofactor (positive control) and the bottom panel in assay in the presence of NOG **6** which does not inhibit WT KDM4C (A) but inhibits the mutant completely at 50 µM concentration (B). The demethylase activity of F187A mutant is significantly low compared to the other analogue-sensitive mutants of the KDM4 subfamily.

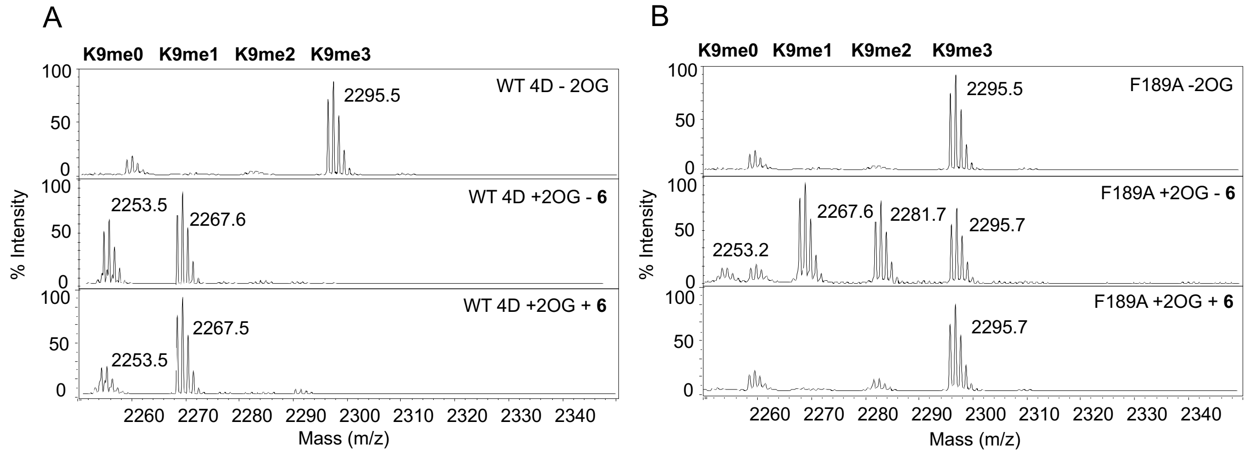

Supplementary Figure S15. MALDI-TOF spectra for demethylase activity of wild type KDM4D (A) and KDM4D-F189A mutant (B). In each case, topmost panel is assay without the cofactor 2OG (negative control), middle panel is assay with the cofactor (positive control) and the bottom panel in assay in the presence of NOG **6** which does not inhibit WT KDM4D (A) but inhibits the mutant completely (B) at 50 µM concentration.

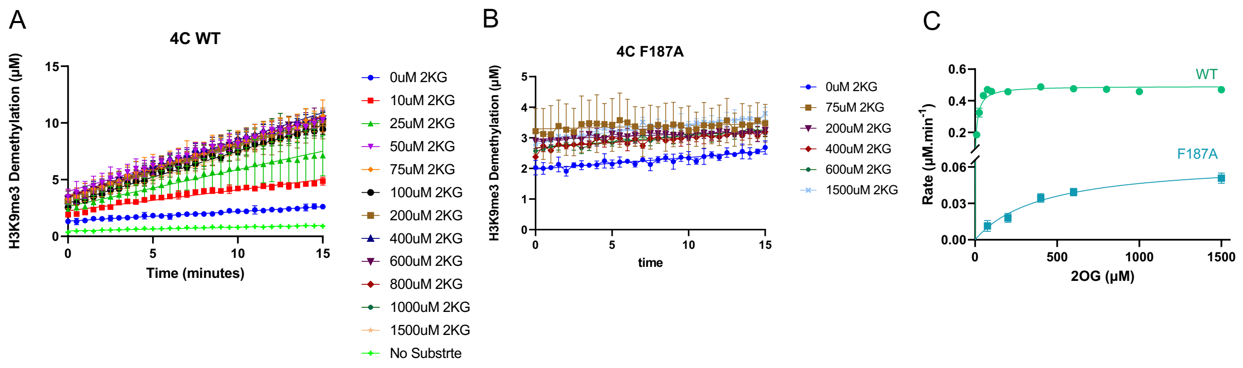

Supplementary Figure S16. Kinetic constants of the wild type and mutant KDM4C. (**A-B**) Time- and 2OG concentration-dependent increase of NADH fluorescence intensity as measure of the catalytic activity of wild type KDM4C-**1** (**A**), and F187A-**1** (**B**) pairs. (**C**) Michaelis-Menten curves for KDM4C-**1** and F187A-**1** pairs to determine the kinetic parameters (k_{cat}, K_M and k_{cat}/K_M).

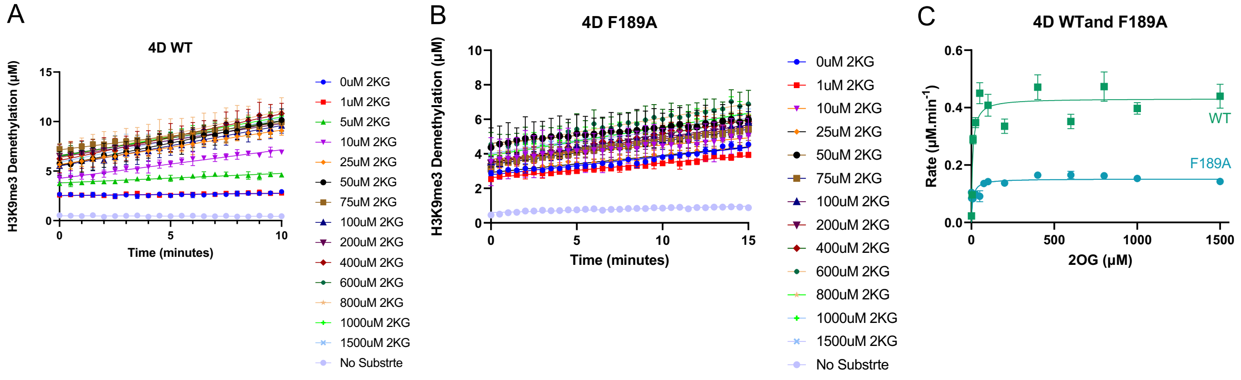

Supplementary Figure S17. Kinetic constants of the wild type and mutant KDM4D. (**A-B**) Time- and 2OG concentration-dependent increase of NADH fluorescence intensity as measure of the catalytic activity of wild type KDM4D-**1** (**A**), and F189A-**1** (**B**) pairs. **(C)** Michaelis-Menten curves for KDM4D-**1** and F189A-**1** pairs to determine the kinetic parameters (k_{cat}, K_M and k_{cat}/K_M).

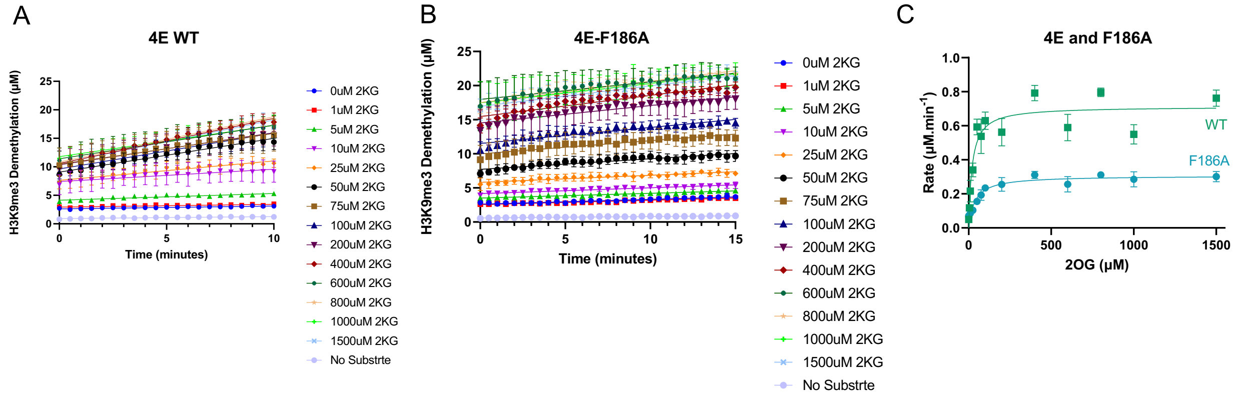

Supplementary Figure S18. Kinetic constants of the wild type and mutant KDM4E. (**A-B**) Time- and 2OG concentration-dependent increase of NADH fluorescence intensity as measure of the catalytic activity of wild type KDM4E-**1** (**A**), and F186A-**1** (**B**) pairs. **(C)** Michaelis-Menten curves for KDM4E-**1** and F189A-**1** pairs to determine the kinetic parameters (k_{cat}, K_M and k_{cat}/K_M).

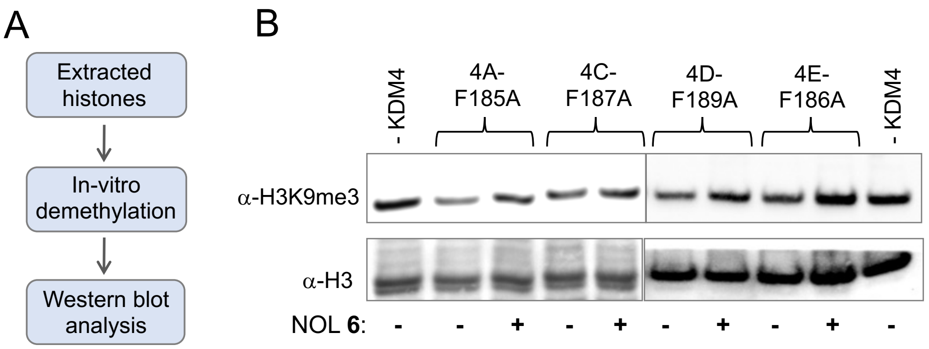

Supplementary Figure S19. Inhibitory activity of NOL **6** towards *as*KDM4 mutants on full-length histone H3. (A) Flow-chart showing *in-vitro* demethylation of full-length H3 extracted from HEK293T cells. (B) Western blotting with anti H3K9me3 antibody confirms demethylase activity of *as*KDM4 mutants (KDM4A-F185A, KDM4C-F187A, KDM4D-F189A and KDM4E-F186A) on extracted H3. NOL **6** at 20 μM concentration inhibits the mutants.

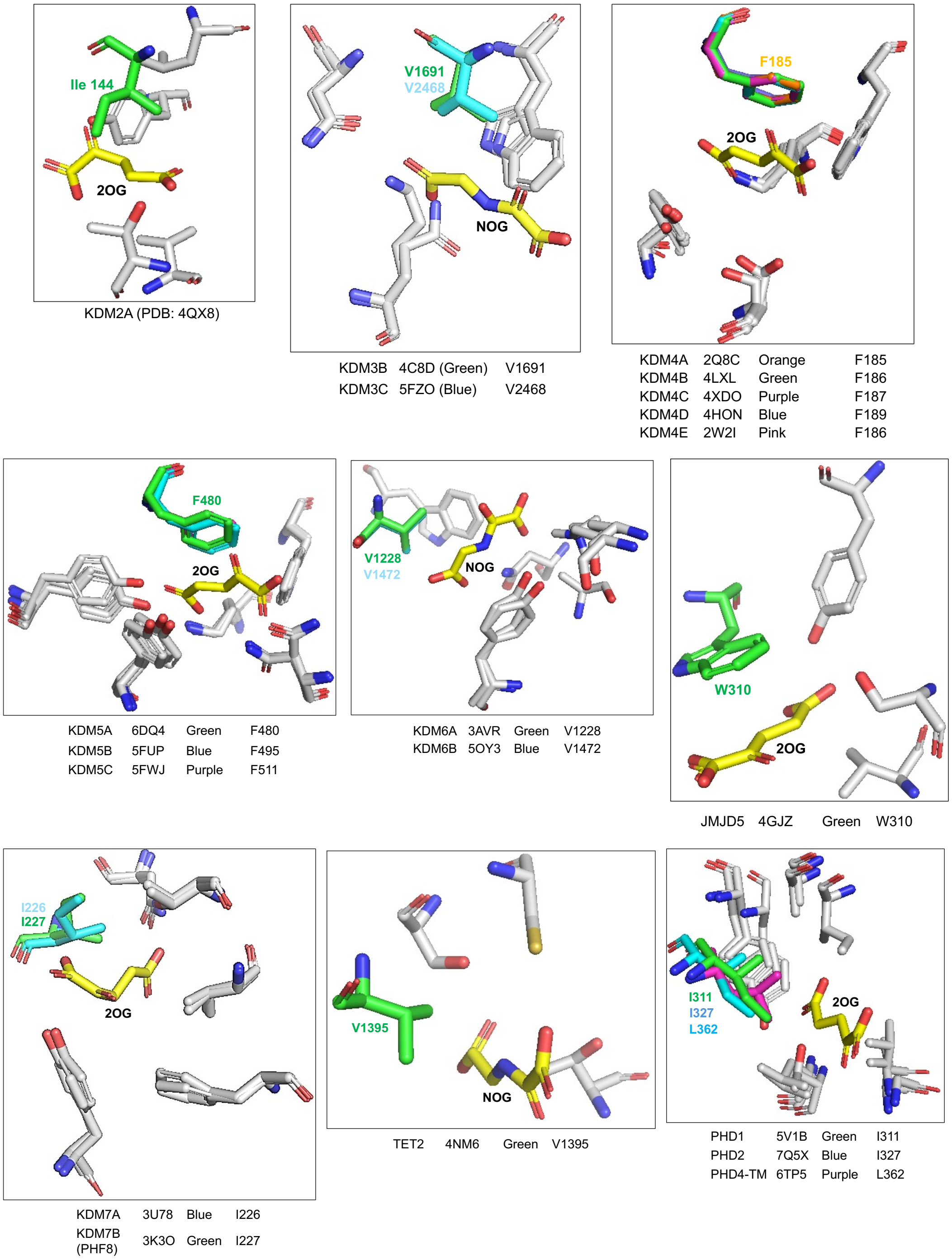

Supplementary Figure S20. Analyses of the crystal structures of 29 2OG enzymes using PyMol molecular visualization system. The Protein Data Bank (PDB) code for each structure is provided. Superimposed structures are generated based on the coordinates of 2OG or NOG. The potential gatekeeper residues are labeled.

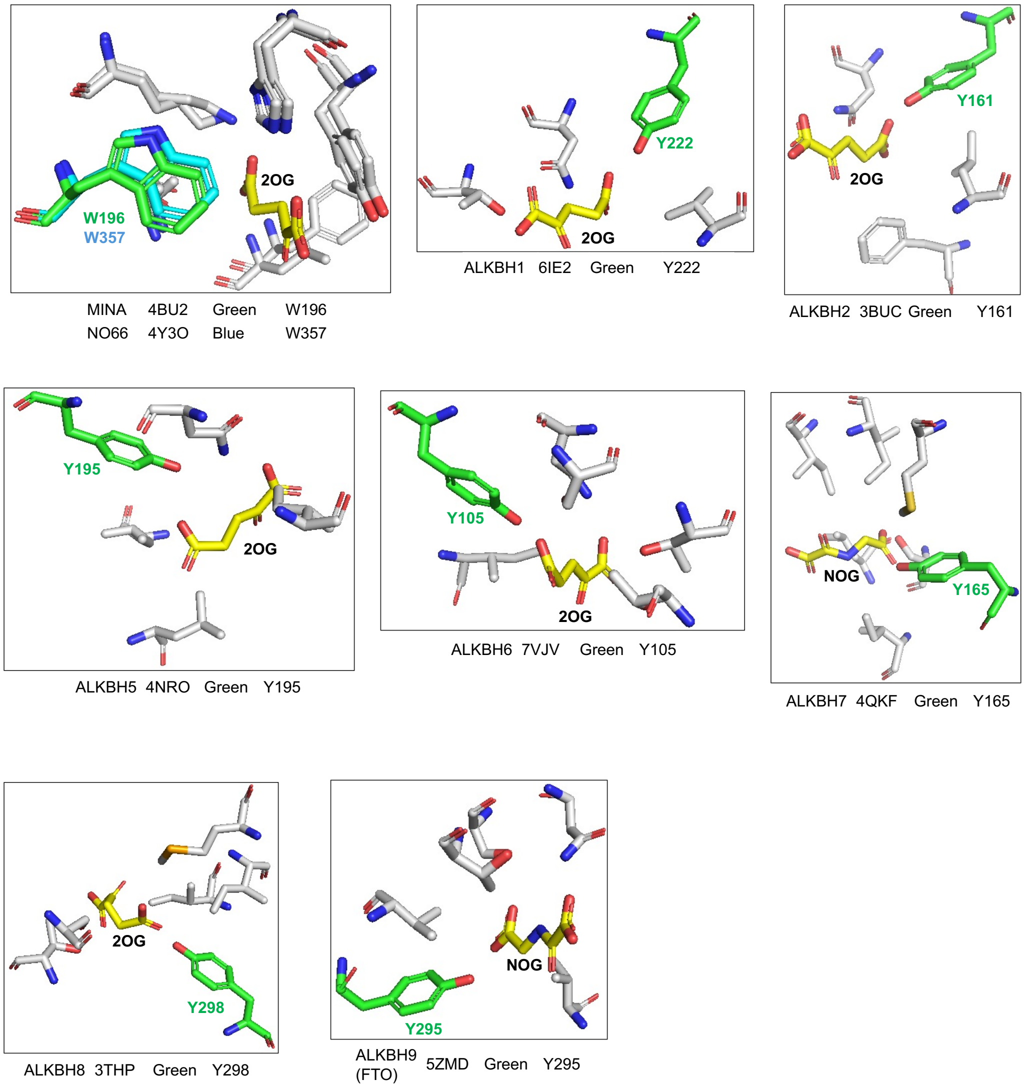

Supplementary Figure S20 continued. Analyses of the crystal structures of 29 2-OG enzymes using PyMol molecular visualization system. The Protein Data Bank (PDB) code for each structure is provided. Superimposed structures are generated based on the coordinates of 2OG or NOG. The potential gatekeeper residues are labeled.

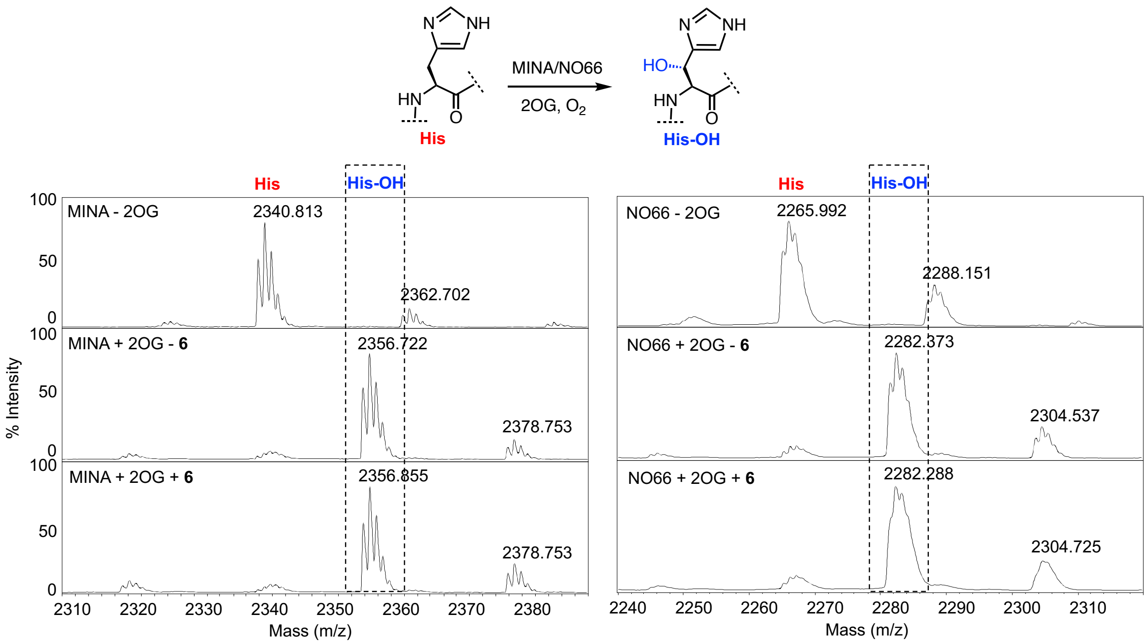

Supplementary Figure S21. Representative MALDI-TOF spectra for enzymatic activity of MINA (left panel) and NO66 (right panel) towards (NH_2-PVEHPFGGGN**H**QHIGKPSTIR-$CONH_2$ for NO66 and NH_2-PGGRGNAGGL**H**HHRINFDKYH-$CONH_2$ for MINA) peptide. No inhibition was observed by NOL **6**.

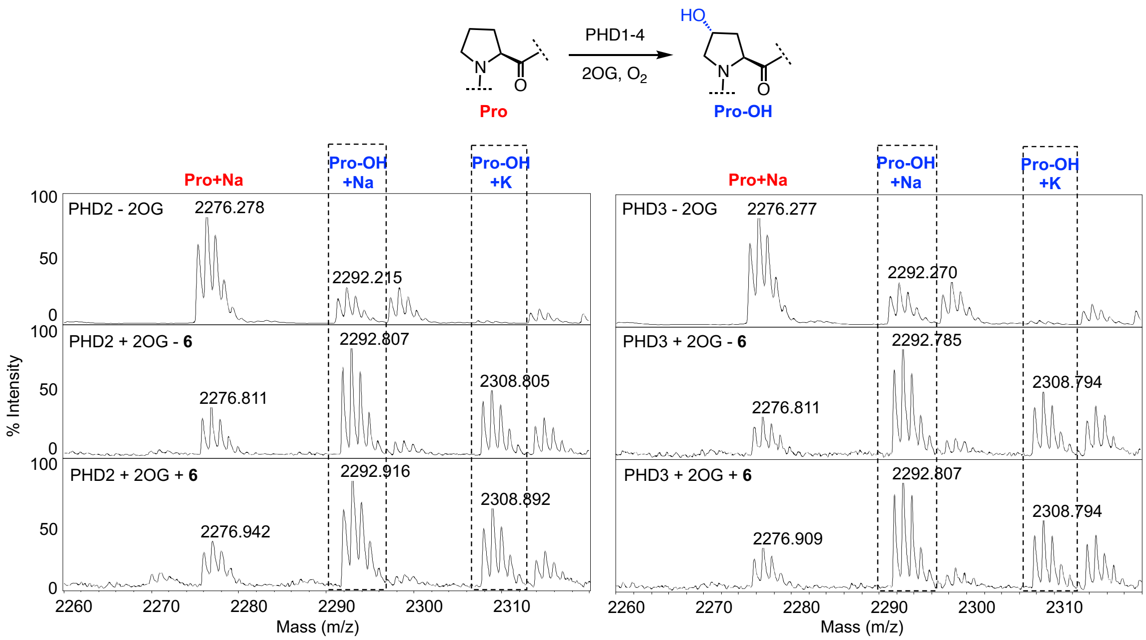

Supplementary Figure S22. Representative MALDI-TOF spectra for enzymatic activity of PHD2 (left panel) and PHD3 (right panel) towards (NH_2-DLDLEMLAPYIPMDDDFQL-$CONH_2$) peptide. No inhibition was observed by NOL **6**.

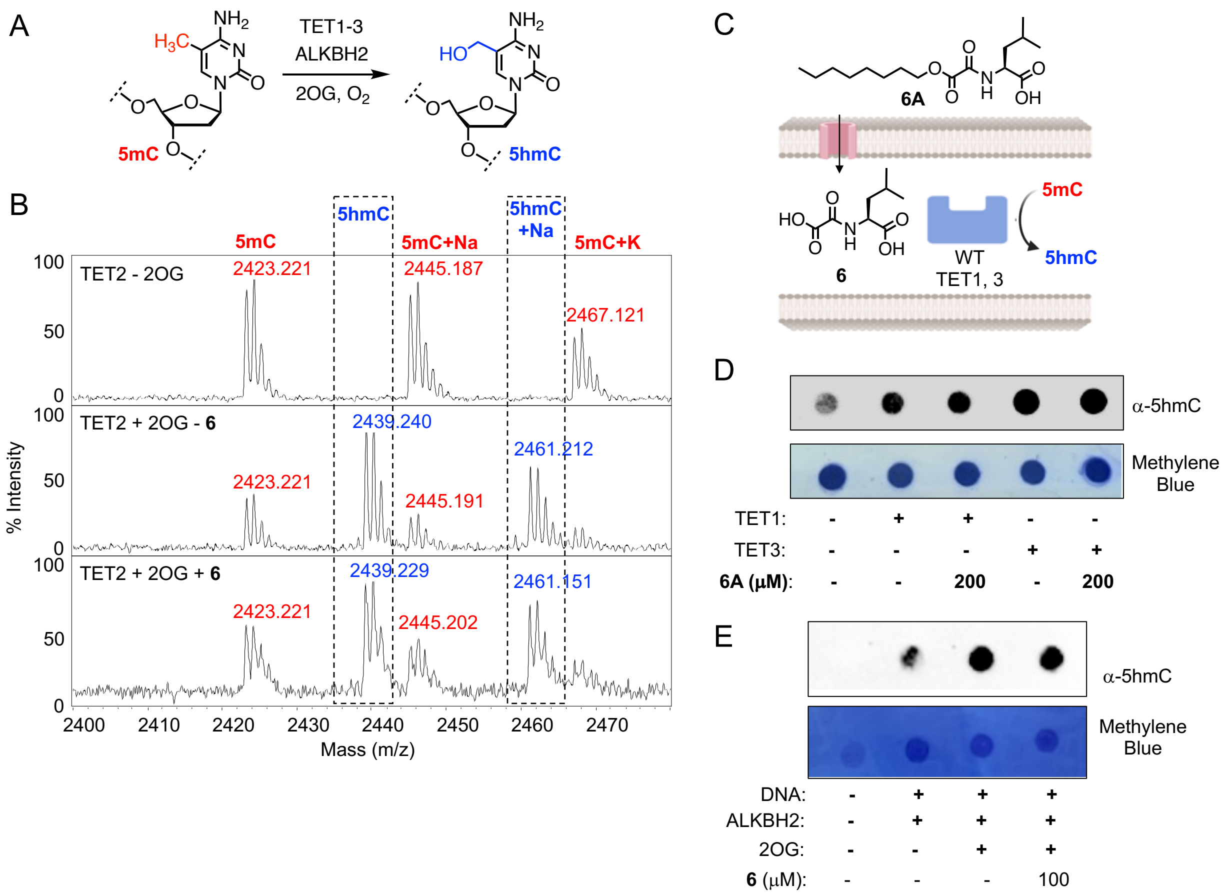

Supplementary Figure S23. Enzymatic activity of TET1-3, and ALKBH2. (A) Schematic of 5mC oxidation to 5hmC by TET1-3. (B) Representative MALDI-TOF spectra for enzymatic activity of TET2 towards short oligonucleotide (5'-CAC5mCGGTG-3' and 5'-CAC5mCGGTG-3' Identical palindromic sequences). No inhibition was observed by NOL **6**. (C) Schematic of activity of full-length wild type TET1 and 3 in absence and presence cell-permeable ester **6A** in HEK293T cells. (D) Dot-blot assay with 5hmC antibody on isolated genomic DNA confirms 5hmC formation by TET1 and 3 in HEK293T cells. No inhibition was observed by **6A**. Methylene blue staining is for loading control. (E) In-vitro activity of bacterially expressed recombinant ALKBH2 towards genomic DNA isolated from HEK293T cells followed by dot-blot assay with 5hmC antibody. No inhibition was observed by NOL **6**.

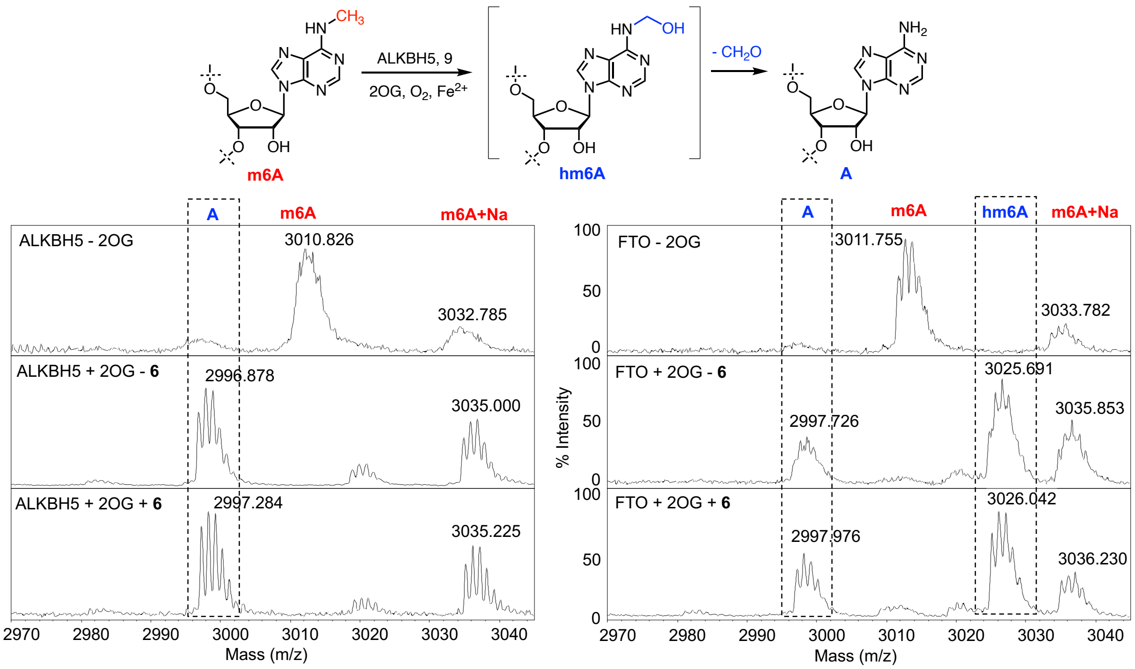

Supplementary Figure S24. Enzymatic activity of ALKBH5 and ALKBH9 (FTO) on 6-methyl adenosine (m6A). Representative MALDI-TOF spectra for enzymatic activity of ALKBH5 (left panel) and FTO (right panel) towards short oligonucleotide (5’-CUGGm6ACUGG-3’). No inhibition was observed by NOL **6**. Formation of 6-hydroxymethyl adenosine (hm6A) intermediate was discernable as reported before.[18, 27]

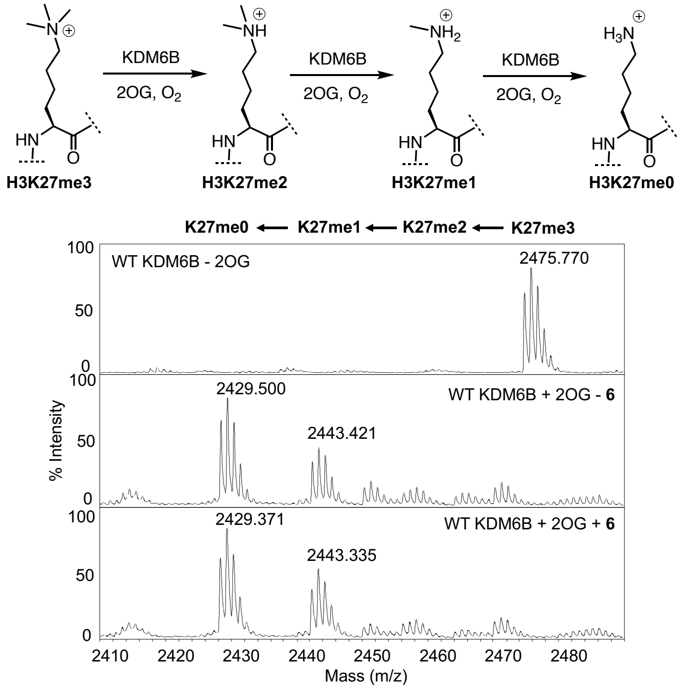

Supplementary Figure S25. Representative MALDI-TOF spectra for enzymatic activity of KDM6B towards (H_2N-APRKQLATKAARK(me3)SAPATGGVK-$CONH_2$) peptide. No inhibition was observed by NOL **6**.

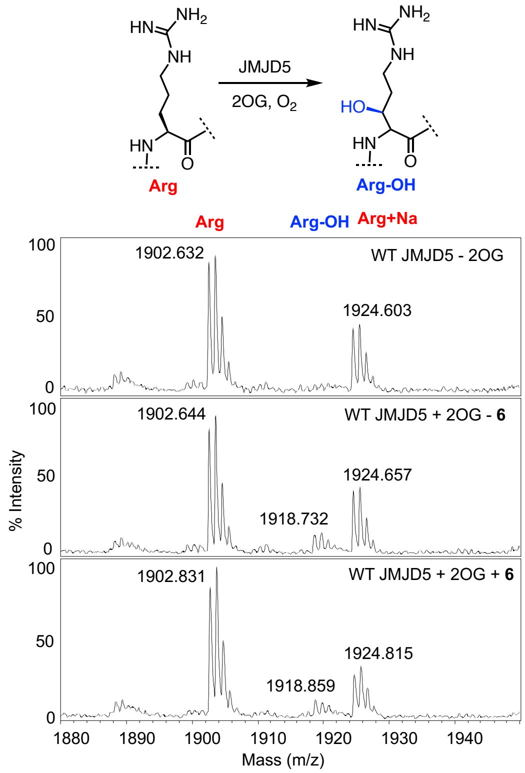

Supplementary Figure S26. Representative MALDI-TOF spectra for enzymatic activity of JMJD5 towards (NH_2-VPRRLGPK**R**ASRIRKL-$CONH_2$) peptide; albeit much lower activity despite following the reported method.[28] No inhibition was observed by NOL **6**.

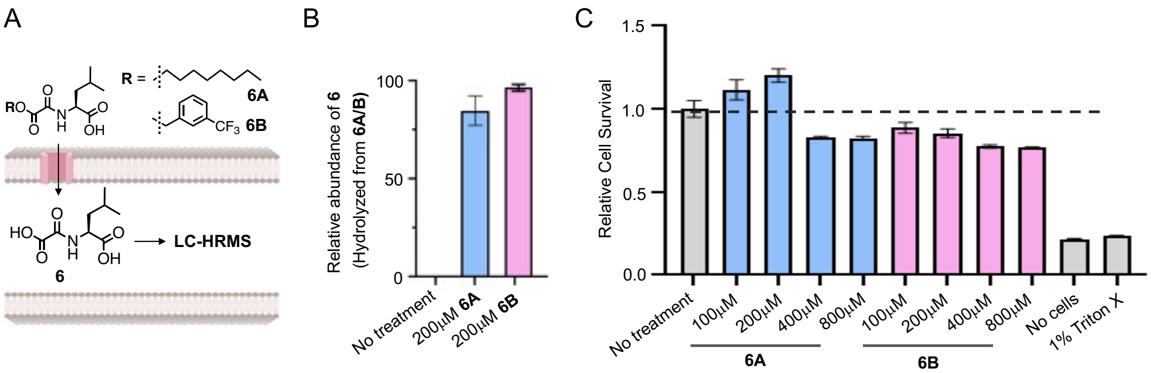

Supplementary Figure S27. Cell-permeability and toxicity of **6A** and **6B**. (A) Schematic showing cell-penetration of **6A/B** followed by hydrolysis to **6**. (B) Quantitative representation of relative

amount of **6** hydrolyzed from **6A/B** in HEK293T cells as determined by LC-HRMS analysis of cell extracts. (C) Dose-dependent effect of **6A/B** on HEK293T cell survival as measured by MTT assay.

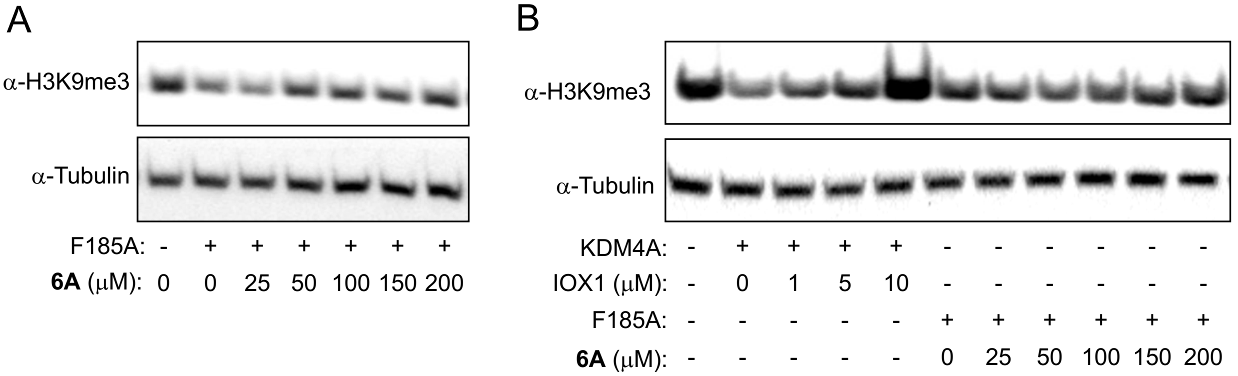

Supplementary Figure S28. Dose-dependent inhibition of F185A by **6A** in HEK293T cells as judged by Western blot analysis. (A-B) are independent biological replicates. The other biological replicates are provided in the Figure 4B.

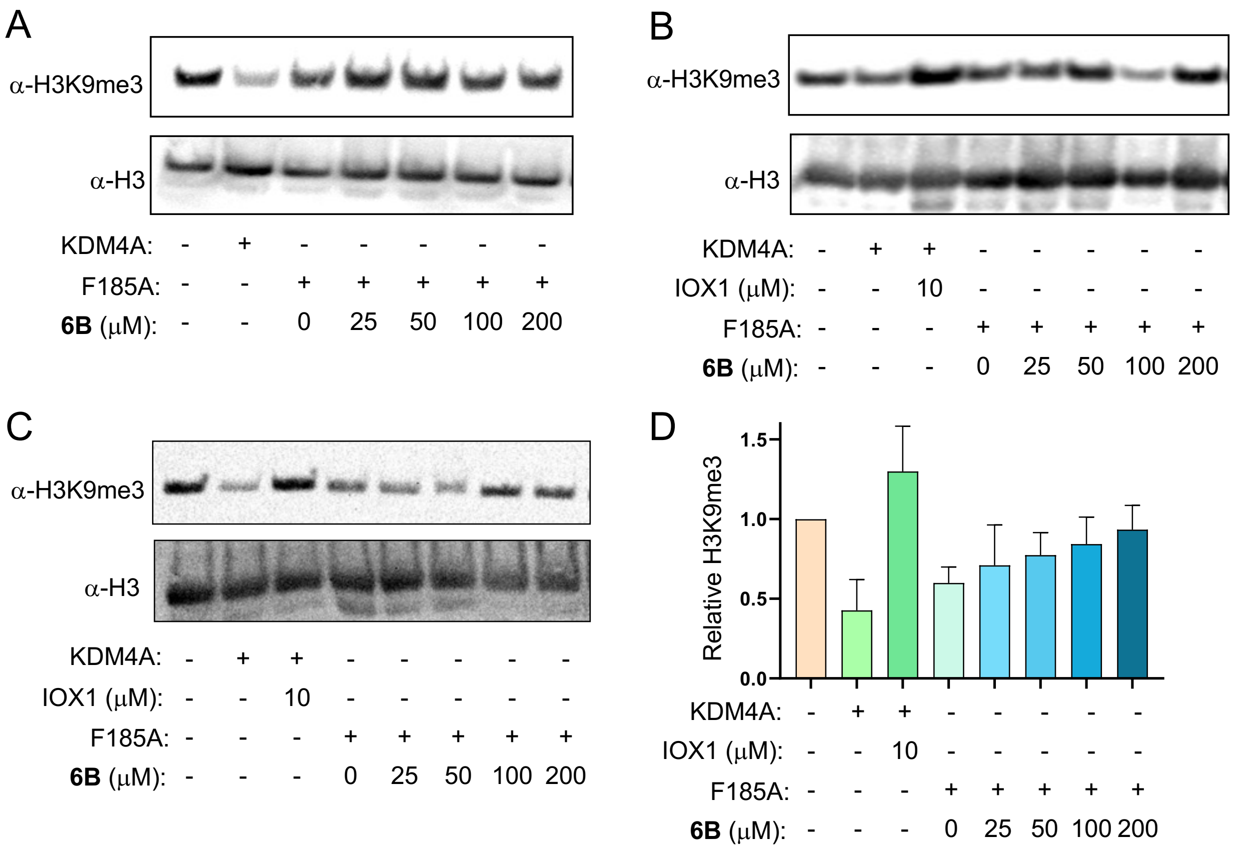

Supplementary Figure S29. Dose-dependent inhibition of F185A by **6B** in HEK293T cells as judged by Western blot analysis. (A-C) are three independent biological replicates; also shown inhibition of wild type KDM4A by n-octyl IOX1 in B and C. (D) Bar diagram representation of inhibition and wild type KDM4A and F185A mutant by IOX1 and **6B**, respectively, based on the Western blot data provided in A-C.

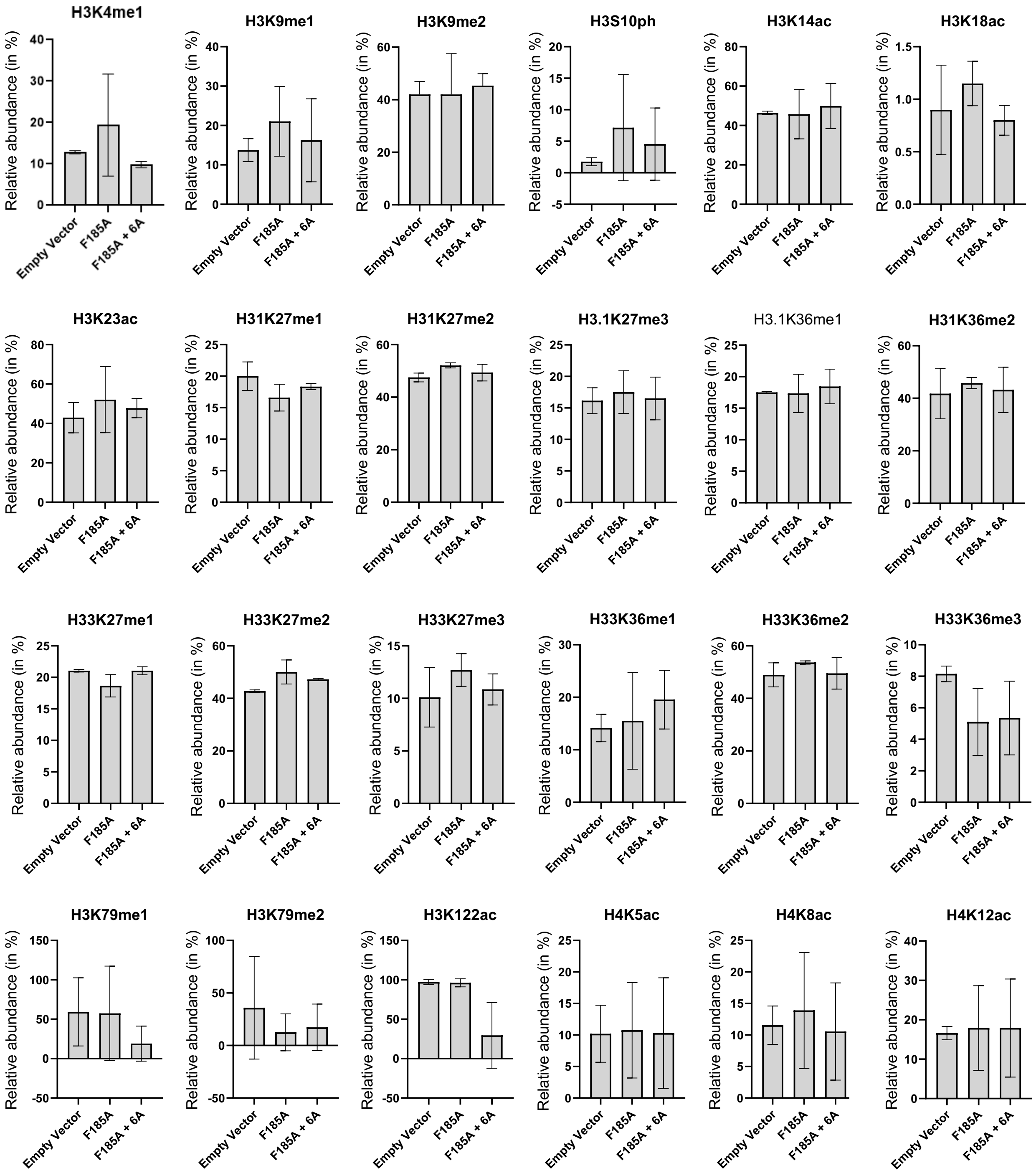

Supplementary Figure S30. Tandem mass spectrometric analyses of key histone modifications in HEK293T cells under indicated conditions. The original proteomic data is provided in Table S1 as a separate file. Histone modifications with >1% relative abundance are shown here.

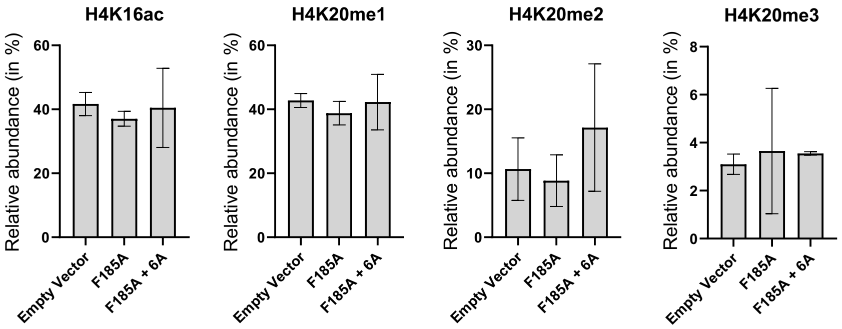

Supplementary Figure S30 continued. Tandem mass spectrometric analyses of key histone modifications in HEK293T cells under indicated conditions. The original proteomic data is provided in Table S1 as a separate file. Histone modifications with >1% relative abundance are shown here.

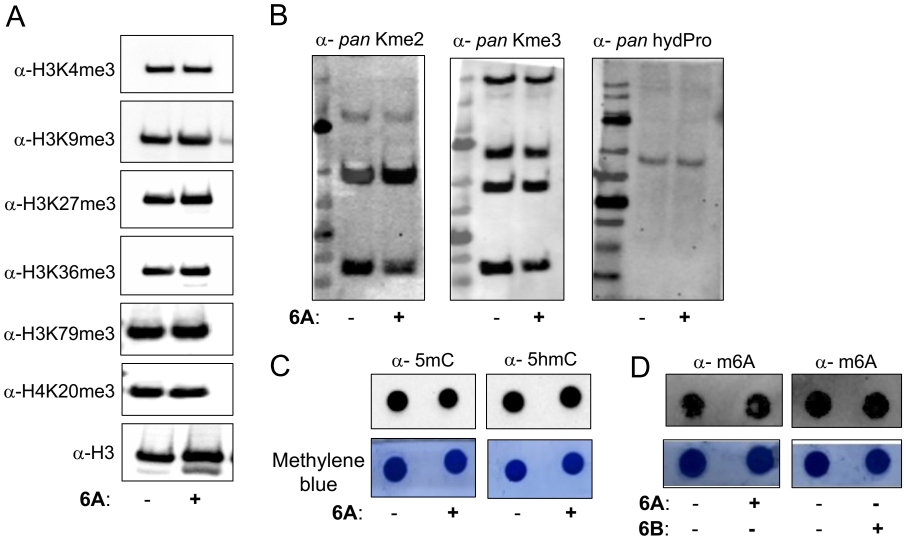

Supplementary Figure S31. In-cell selectivity of **6A/B** towards key post-synthetic modifications. (A) The level of key histone H3 trimethylation (H3K4me3, H3K9me3, H3K27me3, H3K36me3, H3K79me3 and H4K20me3) in HEK293T cells treated with or without 400 μM of n-octyl NOL ester **6A** as revealed by Western blot analysis with appropriate antibodies. (B) Western blot analyses of cellular extracts with *pan* antibodies show **6A** had no inhibitory effect on dimethyllysine, trimethyllysine and hydroxyproline marks on cellular proteins. (C) Isolated genomic DNA from HEK293T cells treated with **6A** was analyzed with 5mC and 5hmC antibodies in dot-blot assay. 6A had no inhibitory effect on the marks. (D) Isolated total RNA from HEK293T cells treated with either **6A** or **6B** was analyzed with m6A antibody in dot-blot assay.

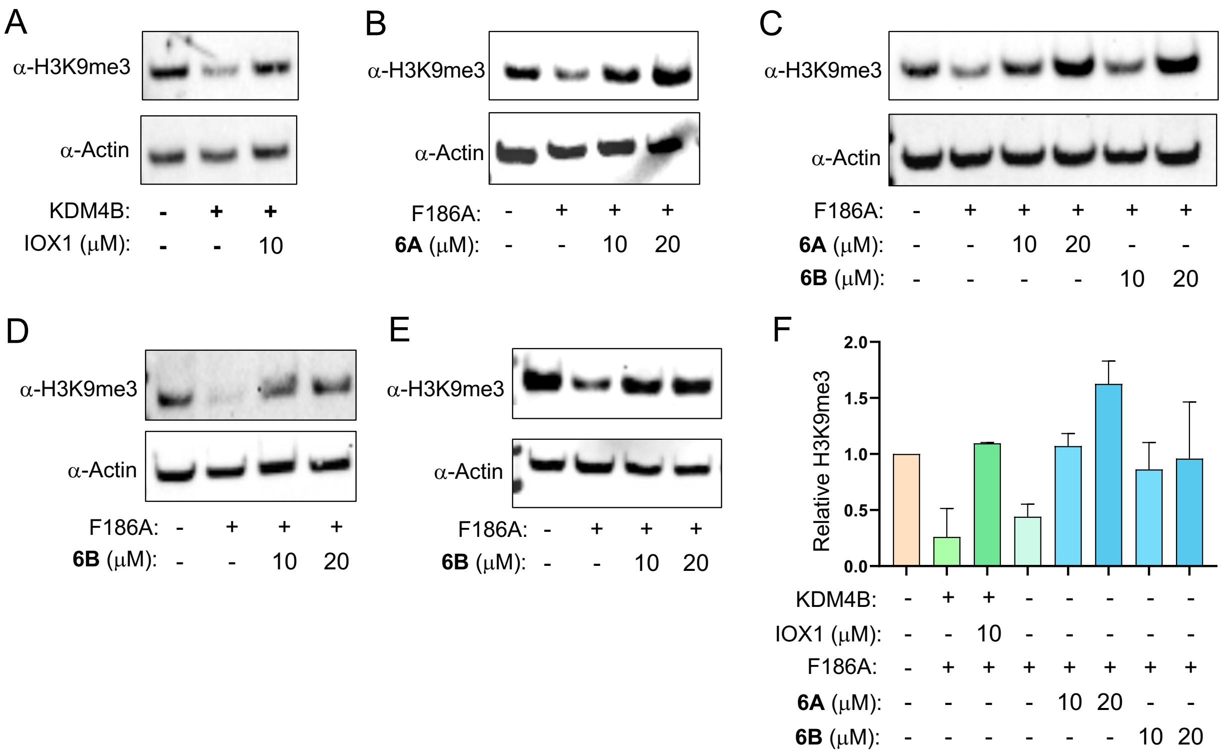

Supplementary Figure S32. Dose-dependent inhibition of wild type KDM4B and its F186A mutant by n-octyl IOX1 and **6A/B** in HEK293T cells as judged by Western blot analysis. (A-E) are independent biological replicates. The other replicates are provided in the Figure 4F in the manuscript. (F) Bar diagram representation of inhibition and wild type KDM4B and F186A mutant by IOX1 and **6A/B**, respectively, based on the Western blot data provided in A-E and Figure 4F.

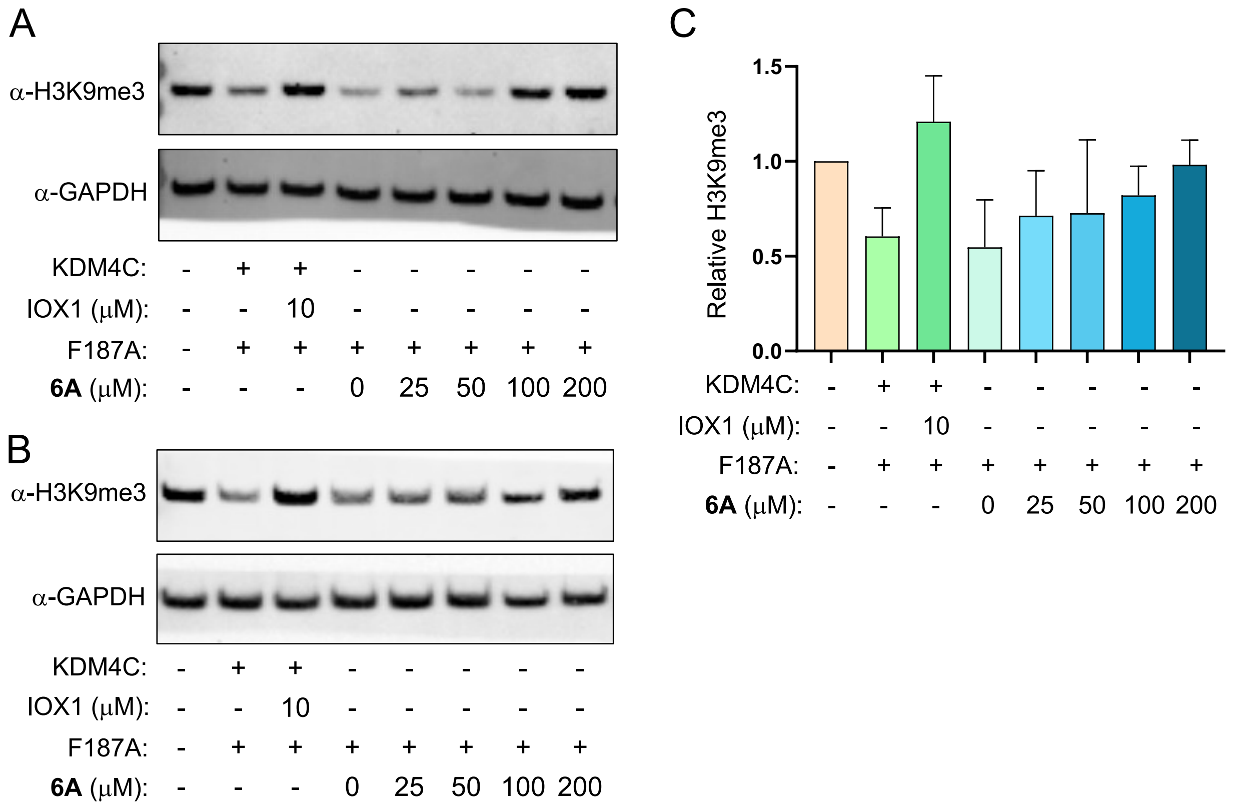

Supplementary Figure S33. Dose-dependent inhibition of wild type KDM4C and its F187A mutant by n-octyl IOX1 and **6A** in HEK293T cells as judged by Western blot analysis. (A-B) are two independent biological replicates. The other replicates are provided in the Figure 4H in the manuscript. (C) Bar diagram representation of inhibition and wild type KDM4C and F187A mutant by IOX1 and **6A**, respectively, based on the Western blot data provided in A-B and Figure 4H.

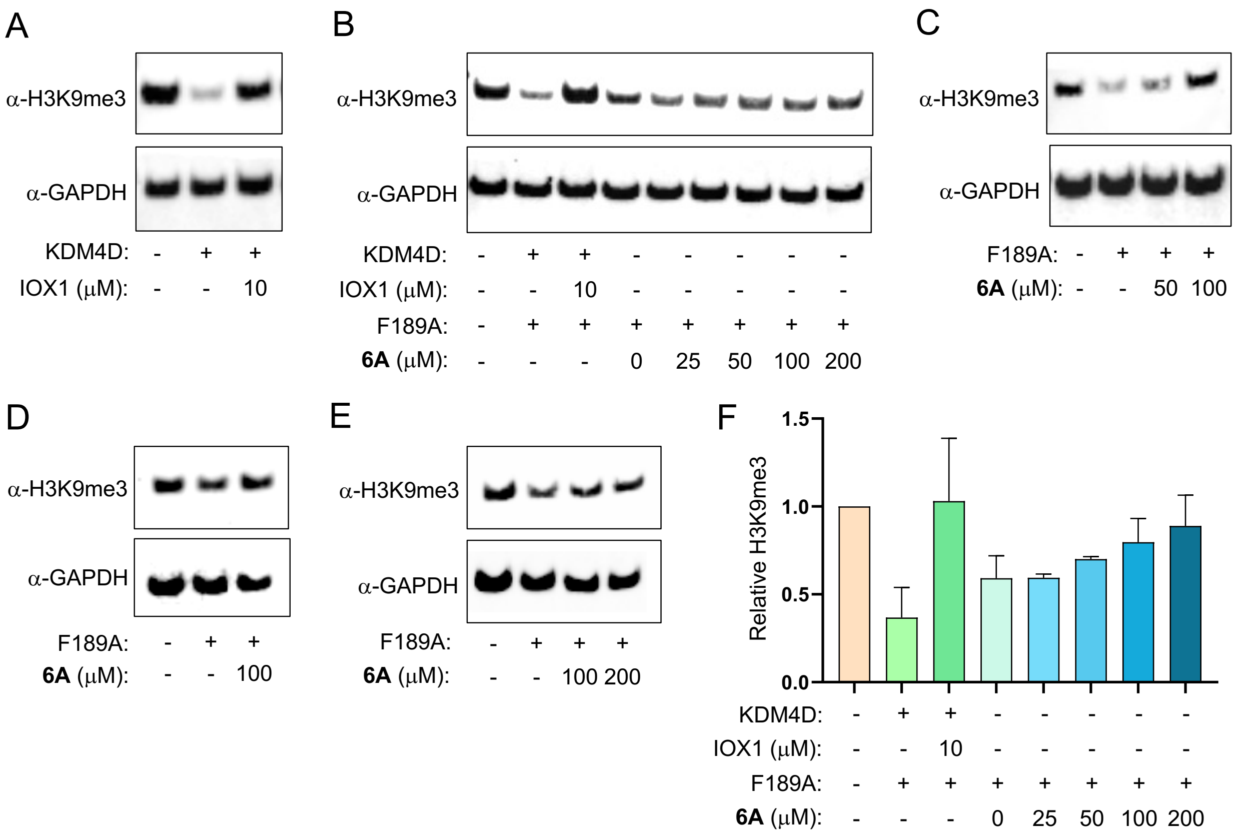

Supplementary Figure S34. Dose-dependent inhibition of wild type KDM4D and its F189A mutant by n-octyl IOX1 and **6A** in HEK293T cells as judged by Western blot analysis. (A-E) are independent biological replicates. The other replicates are provided in the Figure 4J in the manuscript. (F) Bar diagram representation of inhibition and wild type KDM4D and F189A mutant by IOX1 and **6A**, respectively, based on the Western blot data provided in A-E and Figure 4J.

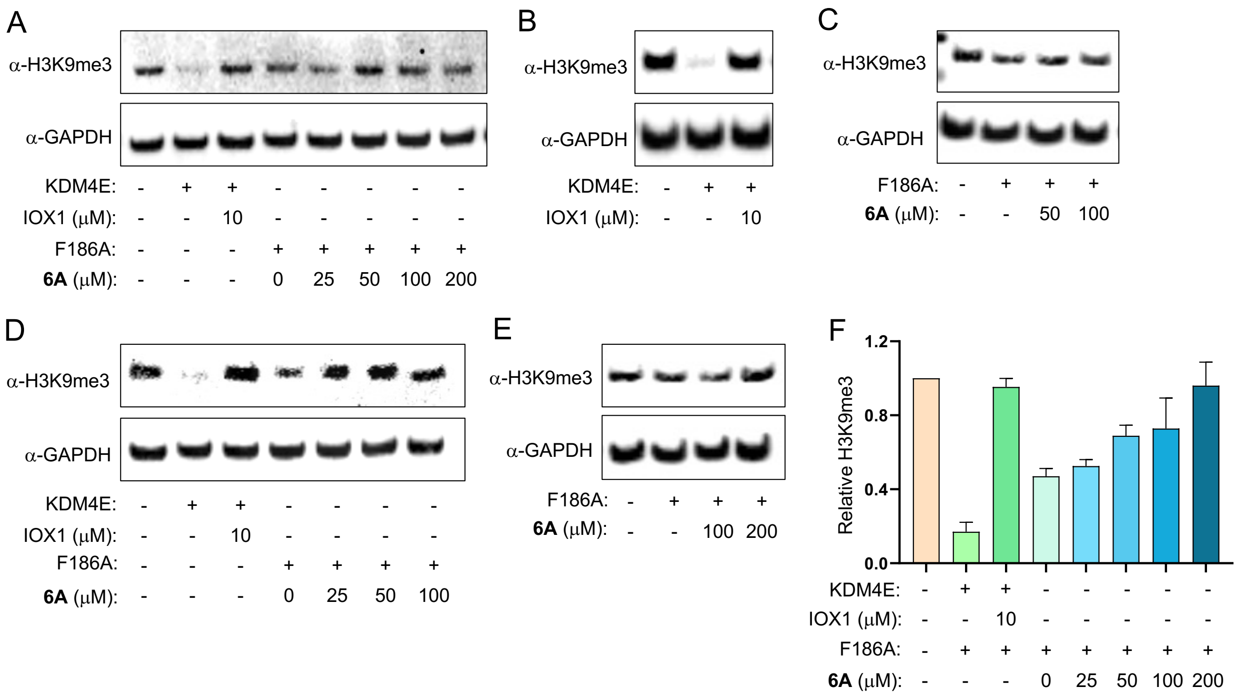

Supplementary Figure S35. Dose-dependent inhibition of wild type KDM4E and its F186A mutant by n-octyl IOX1 and **6A** in HEK293T cells as judged by Western blot analysis. (A-E) are independent biological replicates. The other replicates are provided in the Figure 4L in the manuscript. (F) Bar diagram representation of inhibition and wild type KDM4E and F186A mutant by IOX1 and **6A**, respectively, based on the Western blot data provided in A-E and Figure 4L.

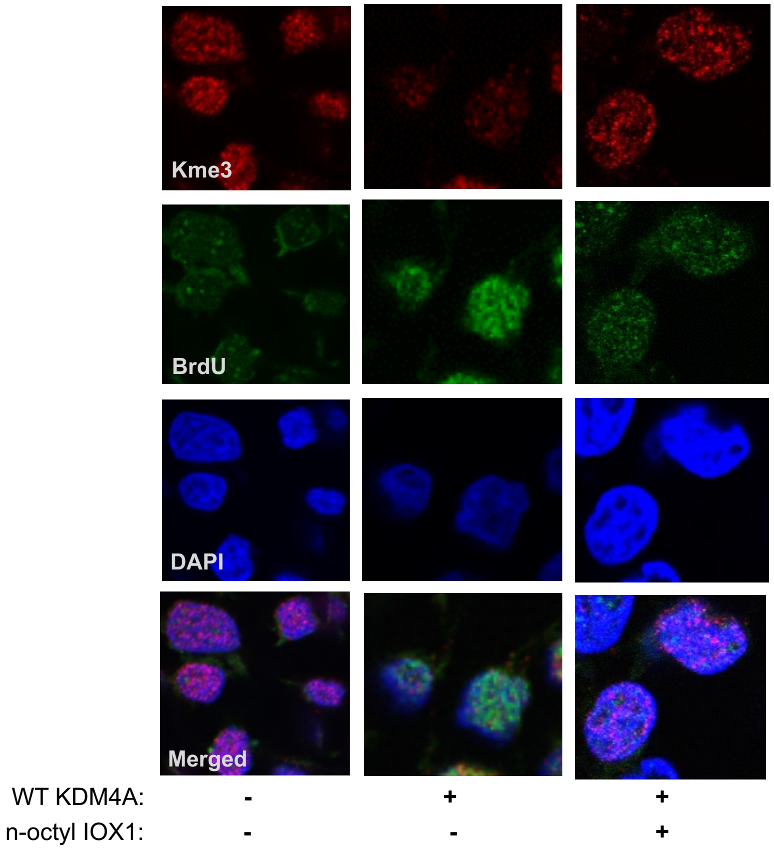

Supplementary Figure S36. Fixed cell immunofluorescence imaging using H3K9me3 and 5-bromouridine (BrdU) antibodies show wild type KDM4A-mediated H3K9me3 demethylation leads to 5FU incorporation into rRNA. Both the processes are inhibited by 10 μM of n-octyl IOX1.

GENE	VECTOR	AFFINITY TAG	RESISTANCE	Bacterial Cells for Expression (Wild type and mutant proteins)
KDM4A	pNIC28-BSA4	N-6x His	Kanamycin	BL21 [DE3] Star
KDM4B	pST4	N-Strep II	Ampicillin	BL21 [DE3] Star
KDM4C	pBh4	N-6x His	Ampicillin	BL21 [DE3] Star
KDM4D	pST4	N-Strep II	Ampicillin	BL21 [DE3] Star
KDM4E	pNIC28-BSA4	N-6x His	Kanamycin	BL21 [DE3] Star
KDM6B	PNH	N-6x His	Kanamycin	BL21 [DE3] Star
FTO (ALKBH9)	pET28a	N-6x His	Kanamycin	BL21 [DE3] Star
ALBH5	PMCSG19	N-6x His	Kanamycin	BL21 [DE3] Star
ALKBH2	pET28a	N-6x His	Kanamycin	BL21 [DE3] Star
EGLN1	pET	N-6x His	Ampicillin	BL21 [DE3] Star
EGLN3	pET	N-6x His	Ampicillin	BL21 [DE3] Star
MINA	pNIC28-Bsa4	N-His-Tev	Kanamycin	BL21 [DE3] Star
C14Orf169 (NO66)	pNIC-CTHF	N-Tev His Flag	Kanamycin	BL21 [DE3] Star
TET2	pET28b	N-6xHis	Kanamycin	BL21 [DE3] Star
FDH	pET28	N-6x His	Kanamycin	BL21 [DE3] Star
hKDM4A	pCMV	N-HA	Ampicillin	N/A
hKDM4B	pCMV	N-HA	Ampicillin	N/A
hKDM4C	pCMV	N-HA	Ampicillin	N/A
hKDM4D	pReceiver-M98	N-3x HA	Ampicillin	N/A
hKDM4E	pRP-EXP	N-HA	Ampicillin	N/A
hTET1	pEF1a	N-HA-Flag	Ampicillin	N/A
hTET3	pEF1a	N-HA-Flag	Ampicillin	N/A

Supplementary Table S2. List of the genes used in the current study. The expression vector, antibiotic resistance and the affinity tag present for protein purification are provided.

Primer Name	Forward Primer Sequence
(h)KDM4A F185A	5’-GGCATGTGGAAGACATCCGCTGCTTGGCACACTGAAGA-3’
(h)KDM4B F186A	5’-CCCTACCTGTACTTCGGCATGTGGAAGACCACCGCGGCCTGGCAC-3’
(h)KDM4C F187A	5’-CTATTTTGGCATGTGGAAGACCACGGCCGCATGGCACACCGAAGACATG**GAC**CTC-3’
(h)KDM4D F189A	5’-GGAAAACCACGGCTGCTTGGCATAC-3’
KDM4E F186A	5’-ATGTGGAAGACCACGGCCGCCTGGCACACAGA-3’
hKDM4E F186A	5’-CTGTACTTTGGCATGTGGAAGACCACGGCCGCCTGGCACACAGAGGACATGGACC-3’

Supplementary Table S3. List of primers designed for site-directed mutagenesis. Reverse primers used are the reverse-complement to the given forward primers.

**Primer Name**	**Sequence**
Actin_Forward (qRTPCR)	5'-GAACGGTGGTGTGTCGTTC-3'
Actin_Reverse (qRTPCR)	5'-GCGTCTCGTCTCGTCTCACT-3'
H2Av_Forward (qRTPCR)	5'-CAAGGCTAAGGCAGTATCTCG-3'
H2Av_Reverse (qRTPCR)	5'-GGTACTCCAGAATCGCAGCAC-3'
KDM4A_Forward (qRTPCR)	5'-AGGAGAGTGAACTGCCTCCA-3'
KDM4A_Reverse (qRTPCR)	5'-GGTCTCCTTCCTCTCCATCC-3'
rRNA_Forward (ChIP-qPCR)	5'-GCCCCGGGGGAGGTAT-3'
rRNA_Reverse (ChIP-qPCR)	5'-GAGGACAGCGTGTCAGCAATAA-3'
FAM-oligo	5'-56-FAM/CGAGAACGCCTGACACGCAC-3'

Supplementary Table S4. List of primers designed for quantitative PCR.

Antibody	Vendor/Catalogue #	Dilution used
H3	CST (9715S)	1:10000
K4me3	Active Motif (61379)	1:2000
K27me3	CST (9733S)	1:1000
H3K9me3	CST (13969S)	1:2000
K36me3	CST (4909S)	1:1000
K79me3	CST (4260S)	1:1000
m6A	Abclonal (A17924)	1:5000
5mC	abcam (214727)	1:2000
5hmC	Active Motif (39769)	1:10000
Hydroxyproline	Cloud-ConeCorp (PAA621Ge01)	1:500
Tubulin	CST (2144)	1:10000
GAPDH	CST (2118S)	1:20000
Actin	CST (3700S)	1:20000
HA	CST (3724S)	1:20000
Anti-rabbit IgG HRP linked antibody	CST (7074S)	1:10000
HRP conjugated Anti mouse IgG	CST (7076S)	1:10000
BrdU	BD Biosciences (BDB555627)	1:500
AlexaFluor-488	Invitrogen (A-11001)	1:1000

Cy3	Invitrogen (A10520)	1:1000

Supplementary Table S5. List of primers designed for quantitative PCR.

24. References

(1) McDonough, M. A.; McNeill, L. A.; Tilliet, M.; Papamicael, C. A.; Chen, Q. Y.; Banerji, B.; Hewitson, K. S.; Schofield, C. J., Selective inhibition of factor inhibiting hypoxia-inducible factor. *J. Am. Chem. Soc.* **2005,** *127* (21), 7680-1.

(2) Woon, E. C.; Demetriades, M.; Bagg, E. A.; Aik, W.; Krylova, S. M.; Ma, J. H.; Chan, M.; Walport, L. J.; Wegman, D. W.; Dack, K. N.; McDonough, M. A.; Krylov, S. N.; Schofield, C. J., Dynamic combinatorial mass spectrometry leads to inhibitors of a 2-oxoglutarate-dependent nucleic acid demethylase. *J. Med. Chem.* **2012,** *55* (5), 2173-84.

(3) Sudhamalla, B.; Wang, S.; Snyder, V.; Kavoosi, S.; Arora, S.; Islam, K., Complementary Steric Engineering at the Protein-Ligand Interface for Analogue-Sensitive TET Oxygenases. *J. Am. Chem. Soc.* **2018,** *140* (32), 10263-10269.

(4) Wagner, S.; Waldman, M.; Arora, S.; Wang, S.; Scott, V.; Islam, K., Allele-Specific Inhibition of Histone Demethylases. *ChemBioChem* **2019,** *20* (9), 1133-1138.

(5) Breski, M.; Dey, D.; Obringer, S.; Sudhamalla, B.; Islam, K., Engineering Biological C–H Functionalization Leads to Allele-Specific Regulation of Histone Demethylases. *J. Am. Chem. Soc.* **2016,** *138* (41), 13505-13508.

(6) Krishnan, S.; Trievel, R. C., Structural and functional analysis of JMJD2D reveals molecular basis for site-specific demethylation among JMJD2 demethylases. *Structure* **2013,** *21* (1), 98-108.

(7) Krishnan, S.; Collazo, E.; Ortiz-Tello, P. A.; Trievel, R. C., Purification and assay protocols for obtaining highly active Jumonji C demethylases. *Anal. Biochem.* **2012,** *420* (1), 48-53.

(8) Pack, L. R.; Yamamoto, K. R.; Fujimori, D. G., Opposing Chromatin Signals Direct and Regulate the Activity of Lysine Demethylase 4C (KDM4C). *J. Biol. Chem.* **2016,** *291* (12), 6060-70.

(9) Scott, V.; Dey, D.; Kuwik, J.; Hinkelman, K.; Waldman, M.; Islam, K., Allele-Specific Chemical Rescue of Histone Demethylases Using Abiotic Cofactors. *ACS Chem. Biol.* **2021**.

(10) Nowak, R. P.; Tumber, A.; Hendrix, E.; Ansari, M. S. Z.; Sabatino, M.; Antonini, L.; Andrijes, R.; Salah, E.; Mautone, N.; Pellegrini, F. R.; Simelis, K.; Kawamura, A.; Johansson, C.; Passeri, D.; Pellicciari, R.; Ciogli, A.; Del Bufalo, D.; Ragno, R.; Coleman, M. L.; Trisciuoglio, D.; Mai, A.; Oppermann, U.; Schofield, C. J.; Rotili, D., First-in-Class Inhibitors of the Ribosomal Oxygenase MINA53. *J. Med. Chem.* **2021,** *64* (23), 17031-17050.

(11) Wang, C.; Zhang, Q.; Hang, T.; Tao, Y.; Ma, X.; Wu, M.; Zhang, X.; Zang, J., Structure of the JmjC domain-containing protein NO66 complexed with ribosomal protein Rpl8. *Acta Crystallogr. D Biol. Crystallogr.* **2015,** *71* (Pt 9), 1955-64.

(12) Wong, S. J.; Ringel, A. E.; Yuan, W.; Paulo, J. A.; Yoon, H.; Currie, M. A.; Haigis, M. C., Development of a colorimetric alpha-ketoglutarate detection assay for prolyl hydroxylase domain (PHD) proteins. *J. Biol. Chem.* **2021,** *296*, 100397.

(13) Jones, S. E.; Olsen, L.; Gajhede, M., Structural Basis of Histone Demethylase KDM6B Histone 3 Lysine 27 Specificity. *Biochem.* **2018,** *57* (5), 585-592.

(14) Chen, F.; Bian, K.; Tang, Q.; Fedeles, B. I.; Singh, V.; Humulock, Z. T.; Essigmann, J. M.; Li, D., Oncometabolites d- and l-2-Hydroxyglutarate Inhibit the AlkB Family DNA Repair Enzymes under Physiological Conditions. *Chem. Res. Toxicol.* **2017,** *30* (4), 1102-1110.

(15) Aik, W.; Demetriades, M.; Hamdan, M. K.; Bagg, E. A.; Yeoh, K. K.; Lejeune, C.; Zhang, Z.; McDonough, M. A.; Schofield, C. J., Structural basis for inhibition of the fat mass and obesity associated protein (FTO). *J. Med. Chem.* **2013,** *56* (9), 3680-8.

(16) Roy, T. W.; Bhagwat, A. S., Kinetic studies of Escherichia coli AlkB using a new fluorescence-based assay for DNA demethylation. *Nucleic Acids Res.* **2007,** *35* (21), e147.

(17) Hopkinson, R. J.; Langley, G. W.; Belle, R.; Walport, L. J.; Dunne, K.; Munzel, M.; Salah, E.; Kawamura, A.; Claridge, T. D. W.; Schofield, C. J., Human histone demethylase KDM6B can catalyse sequential oxidations. *Chem. Commun.* **2018,** *54* (57), 7975-7978.

(18) Toh, J. D. W.; Crossley, S. W. M.; Bruemmer, K. J.; Ge, E. J.; He, D.; Iovan, D. A.; Chang, C. J., Distinct RNA N-demethylation pathways catalyzed by nonheme iron ALKBH5 and FTO enzymes enable regulation of formaldehyde release rates. *Proc. Natl. Acad. Sci. USA* **2020,** *117* (41), 25284-25292.

(19) Hirsila, M.; Koivunen, P.; Gunzler, V.; Kivirikko, K. I.; Myllyharju, J., Characterization of the human prolyl 4-hydroxylases that modify the hypoxia-inducible factor. *J. Biol. Chem.* **2003,** *278* (33), 30772-80.

(20) Shechter, D.; Dormann, H. L.; Allis, C. D.; Hake, S. B., Extraction, purification and analysis of histones. *Nat. Protoc.* **2007,** *2* (6), 1445-57.

(21) Hopkinson, R. J.; Tumber, A.; Yapp, C.; Chowdhury, R.; Aik, W.; Che, K. H.; Li, X. S.; Kristensen, J. B.; King, O. N.; Chan, M. C.; Yeoh, K. K.; Choi, H.; Walport, L. J.; Thinnes, C. C.; Bush, J. T.; Lejeune, C.; Rydzik, A. M.; Rose, N. R.; Bagg, E. A.; McDonough, M. A.; Krojer, T.; Yue, W. W.; Ng, S. S.; Olsen, L.; Brennan, P. E.; Oppermann, U.; Muller-Knapp, S.; Klose, R. J.; Ratcliffe, P. J.; Schofield, C. J.; Kawamura, A., 5-Carboxy-8-hydroxyquinoline is a Broad Spectrum 2-Oxoglutarate Oxygenase Inhibitor which Causes Iron Translocation. *Chem. Sci.* **2013,** *4* (8), 3110-3117.

(22) King, O. N.; Li, X. S.; Sakurai, M.; Kawamura, A.; Rose, N. R.; Ng, S. S.; Quinn, A. M.; Rai, G.; Mott, B. T.; Beswick, P.; Klose, R. J.; Oppermann, U.; Jadhav, A.; Heightman, T. D.; Maloney, D. J.; Schofield, C. J.; Simeonov, A., Quantitative high-throughput screening identifies 8-hydroxyquinolines as cell-active histone demethylase inhibitors. *PloS One* **2010,** *5* (11), e15535.

(23) Neilson, K. A.; Ali, N. A.; Muralidharan, S.; Mirzaei, M.; Mariani, M.; Assadourian, G.; Lee, A.; van Sluyter, S. C.; Haynes, P. A., Less label, more free: Approaches in label-free quantitative mass spectrometry. *Proteomic.* **2011,** *11* (4), 535-553.

(24) Dieterich, D. C.; Link, A. J.; Graumann, J.; Tirrell, D. A.; Schuman, E. M., Selective identification of newly synthesized proteins in mammalian cells using bioorthogonal noncanonical amino acid tagging (BONCAT). *Proc. Natl. Acad. Sci. USA* **2006,** *103* (25), 9482-7.

(25) Sidoli, S.; Bhanu, N. V.; Karch, K. R.; Wang, X.; Garcia, B. A., Complete Workflow for Analysis of Histone Post-translational Modifications Using Bottom-up Mass Spectrometry: From Histone Extraction to Data Analysis. *J. Vis. Exp.* **2016,** (111).

(26) Yuan, Z. F.; Sidoli, S.; Marchione, D. M.; Simithy, J.; Janssen, K. A.; Szurgot, M. R.; Garcia, B. A., EpiProfile 2.0: A Computational Platform for Processing Epi-Proteomics Mass Spectrometry Data. *J. Proteom. Res.* **2018,** *17* (7), 2533-2541.

(27) Fu, Y.; Jia, G.; Pang, X.; Wang, R. N.; Wang, X.; Li, C. J.; Smemo, S.; Dai, Q.; Bailey, K. A.; Nobrega, M. A.; Han, K. L.; Cui, Q.; He, C., FTO-mediated formation of N6-hydroxymethyladenosine and N6-formyladenosine in mammalian RNA. *Nat. Commun.* **2013,** *4*, 1798.

(28) Wilkins, S. E.; Islam, M. S.; Gannon, J. M.; Markolovic, S.; Hopkinson, R. J.; Ge, W.; Schofield, C. J.; Chowdhury, R., JMJD5 is a human arginyl C-3 hydroxylase. *Nat. Commun.* **2018,** *9* (1), 1180.
